# The Impact of Including Costs and Outcomes of Dementia in a Health Economic Model to Evaluate Lifestyle Interventions to Prevent Diabetes and Cardiovascular Disease

**DOI:** 10.1177/0272989X20946758

**Published:** 2020-09-19

**Authors:** Penny Breeze, Chloe Thomas, Praveen Thokala, Louise Lafortune, Carol Brayne, Alan Brennan

**Affiliations:** School of Health and Related Research, University of Sheffield, Sheffield, South Yorkshire, UK; School of Health and Related Research, University of Sheffield, Sheffield, South Yorkshire, UK; School of Health and Related Research, University of Sheffield, Sheffield, South Yorkshire, UK; Cambridge Institute of Public Health, University of Cambridge, Cambridge, Cambridgeshire, UK; Cambridge Institute of Public Health, University of Cambridge, Cambridge, Cambridgeshire, UK; School of Health and Related Research, University of Sheffield, Sheffield, South Yorkshire, UK

**Keywords:** cost-effectiveness analysis, dementia, non-communicable disease, public health, simulation

## Abstract

**Objectives:**

Economic evaluations of lifestyle interventions, which aim to prevent diabetes/cardiovascular disease (CVD), have not included dementia. Lifestyle interventions decrease dementia risk and extend life expectancy, leading to competing effects on health care costs. We aim to demonstrate the feasibility of including dementia in a public health cost-effectiveness analysis and quantify the overall impacts accounting for these competing effects.

**Methods:**

The School for Public Health Research (SPHR) diabetes prevention model describes individuals’ risk of type 2 diabetes, microvascular outcomes, CVD, congestive heart failure, cancer, osteoarthritis, depression, and mortality in England. In version 3.1, we adapted the model to include dementia using published data from primary care databases, health surveys, and trials of dementia to describe dementia incidence, diagnosis, and disease progression. We estimate the impact of dementia on lifetime costs and quality-adjusted life years (QALYs) gained of the National Health Service diabetes prevention program (NHS DPP) from an NHS/personal social services perspective with 3 scenarios: 1) no dementia, 2) dementia only, and 3) reduced dementia risk. Subgroup, parameter, and probabilistic sensitivity analyses were conducted.

**Results:**

The lifetime cost savings of the NHS DPP per patient were £145 in the no-dementia scenario, £121 in the dementia-only scenario, and £167 in the reduced dementia risk scenario. The QALY gains increased by 0.0006 in dementia only and 0.0134 in reduced dementia risk. Dementia did not alter the recommendation that the NHS/DPP is cost-effective.

**Conclusions:**

Including dementia into a model of lifestyle interventions was feasible but did not change policy recommendations or modify health economic outcomes. The impact on health economic outcomes was largest where a direct impact on dementia incidence was assumed, particularly in elderly populations.

Hypertension, hypercholesterolemia, smoking, diabetes, obesity, physical inactivity, and stroke are known to be risk factors for dementia.^1–3^ Improvements in midlife obesity, hypertension, and dyslipidemia are believed to reduce the incidence of dementia.^4^ Delaying dementia onset by 1 year could dramatically affect its prevalence, reducing disease frequency by approximately 10%.^5^ Although recent trials for dementia prevention through vascular risk and lifestyle intervention have not identified a reduction in dementia incidence or cognitive decline in the intent-to-treat (ITT) population, subgroup analyses indicate that lifestyle interventions can have small beneficial effects.^6,7^

Diabetes prevention programs (DPPs) are effective in reducing the incidence of diabetes and lowering the risk of cardiovascular diseases.^8–10^ The School for Public Health Research (SPHR) diabetes prevention model estimated that the National Health Service (NHS) DPP in individuals with HbA1c ≥42 mmol/L would generate life year gains, be cost-effective, and offer good return on investment for the NHS.^11^ However, this analysis, like other economic evaluations for obesity and diabetes prevention,^12^ does not include dementia as a health condition. Given the nature of the NHS DPP, with its focus on dietary advice, physical activity, and weight loss, it is conceivable that these interventions, closely similar to those of dementia risk reduction, could improve brain health.

Including dementia adds additional complexity to diabetes and the cardiovascular disease risk model. However, excluding dementia from economic evaluations of lifestyle interventions to targeting diabetes and cardiovascular disease may not account for health care costs in added years of life gained from the intervention and underestimating the intervention cost savings and quality-adjusted life year (QALY) gains from reduced dementia diagnoses. This could lead to an inefficient allocation of resources. It is not known what impact these competing factors will have on the economic case for implementing the program and whether the net effect will improve, or worsen, the cost-effectiveness of lifestyle interventions.

The aim of this study is 3-fold. First, we describe an adaptation to an existing model to evaluate diabetes and cardiovascular disease (CVD) prevention lifestyle interventions and incorporate dementia as an explicit outcome. We describe the methods and sources of data used to estimate dementia incidence, diagnosis, disease progression, costs, and health state utilities. Second, the analysis seeks to investigate the impact on cost-effectiveness outcomes of including dementia by using the NHS DPP as an illustration of how costs and prevention of dementia affect the cost-effectiveness of the intervention. Third, we identify when the inclusion of dementia in a public health model for lifestyle interventions will have the greatest impact on cost-effectiveness outcomes.

## Methods

We developed 3 primary analyses for this study comparing NHS DPP v. no implementation. First, the NHS DPP intervention and control arms were simulated without dementia as a health outcome, using the original model structure with updated metabolic trajectories, costs, and study population. This analysis is labeled as no dementia. Second, the NHS DPP intervention and control arms were simulated with dementia as a health outcome incurring costs, utility decrements, and mortality impacts. Dementia incidence was reduced because fewer individuals develop diabetes and stroke, but we assumed no direct intervention effect. This analysis is labeled as dementia only. Third, the NHS DPP intervention was simulated with dementia as a health outcome and a relative risk reduction in dementia risk derived from changes in metabolic risk factors and the Cardiovascular Risk Factors, Aging, and Incidence of Dementia (CAIDE) risk modification for lifestyle factors. This analysis is labeled as reduced dementia risk.

### Original Diabetes Prevention Model Structure

The SPHR diabetes prevention model was developed to forecast long-term health and health care costs of interventions targeting diabetes prevention in England.^13,14^ The model is an individual patient simulation model and based on the evolution of personalized trajectories for metabolic factors, including body mass index (BMI), systolic blood pressure (SBP), cholesterol, and measures of blood glucose (including HbA1c) based on analyses of the Whitehall II cohort.^15^

An individual-level simulation enables multiple risk factors to affect a wider range of health outcomes and events occurring at various times and stages of life.

The model runs in annual cycles (see schematic in Suppl. Figure S1). For each person, their BMI, cholesterol, SBP, and HbA1c progress from year to year. Every year in the model, an individual may visit their general practitioner (GP) or undergo a health check and be diagnosed with and treated for hypertension, high cardiovascular risk, diabetes, microvascular complications of diabetes, CVD, congestive heart failure, osteoarthritis, depression, and breast or colon cancer. Mortality can occur in any cycle, and individuals are at increased risk of death with a history of cardiovascular disease, cancer, or diabetes. Baseline health-related quality of life (HRQoL) for each individual in the model accounts for variation in the population. In the simulation, HRQoL deteriorates over time with age and medical complications. Each condition is associated with a utility decrement and a health and social care cost based on published evidence. The analysis includes the NHS costs and the social care costs. The model estimates the lifetime costs of interventions and care, together with the lifetime QALYs, in a 2-arm comparison of NHS DPP v. no implementation. Costs and QALYs were discounted at a rate 3.5% per annum.

A full detailed description of the methodology, evidence, data, and assumptions used in the original model can be found in sections 6 to 10 of the supplementary material to this article. The validation of key dementia outcomes is also described in section 13 of the supplementary material. Validation analyses to test the model’s performance in simulating metabolic trajectories, diabetes incidence, cardiovascular disease incidence, blindness, amputation, foot ulcer, and renal failure are described in the supplementary material published with the original article.^13^ The analysis was conducted in version 3.1 of the SPHR diabetes prevention model using R version 3.6.1.

### Updated Metabolic Trajectories in Older Age

To account for changes to metabolic trajectories in older ages, we developed new analyses of the English Longitudinal Study of Ageing^16^ to predict changes in BMI, SBP, cholesterol, and measures of blood glucose (including HbA1c) using analyses for individuals aged 60 years or over (see section 6 of the supplementary material).

### Study Population

The SPHR diabetes prevention model was updated with baseline population from the Health Survey for England (HSE).^17^ HSE 2014 was chosen to inform the baseline population to describe correlations between demographics, metabolic baseline risk factors, and HRQoL from a representative sample of the English population. Individuals meeting 3 eligibility criteria for the NHS DPP (HbA1c 6%–6.4%, aged ≥16, no existing diabetes diagnosis) were selected from the HSE 2014 population. It was assumed that eligible individuals have already been identified as meeting eligibility criteria, and we do not incorporate any costs or utility change associated with identification or referral to the scheme, in line with previous analyses. The characteristics of the eligible population are shown in Table 1.

**Table 1:** Key Model Inputs and Parameters

	Baseline Characteristics of Eligible Population from HSE^17^		
Characteristic	Number	Percentage	
Male	1042	44.7%	
Nonwhite	249	10.7%	
Current smoker	446	19.1%	
Past smoker	684	29.4%	
Hypertension	615	26.4%	
	Mean	SD	Median
Age (years)	57.1	17.6	59
BMI (kg/m^2^)	28.5	5.4	28
Systolic blood pressure (mm Hg)	129.9	17.7	130
Total cholesterol (mmol/L)	5.2	1.1	5.2
HDL cholesterol (mmol/L)	1.5	0.47	1.5
HbA1c (%)	6.2	0.15	6.2
	Mean Effectiveness Evidence from PHE Evidence Review^18^		
	Mean	SE	Source
BMI (kg/m^2^)	−1.47	0.156	(18)
HbA1c (%)	−0.20	0.043	(18)
Systolic blood pressure (mm Hg)	−6.57	0.923	(18)
Total cholesterol (mmol/L)	−0.28	0.028	(18)
Intervention cost	£270		(11)
Duration of effect	5 years		(11)
	Key Dementia Incidence Parameters		
THIN (60–80 years) hazard ratio BMI	0.940	0.0038	(2)
THIN (60–80 years) hazard ratio BMI^2^	1.003	0.0003	(2)
THIN (60–80 years) hazard ratio antihypertensives	0.876	0.0296	(2)
THIN (60–80 years) hazard ratio stroke	1.781	0.0394	(2)
THIN (60–80 years) hazard ratio diabetes	1.332	0.0417	(2)
CAIDE odds ratio obese	2.296	0.3034	(4)
CAIDE odds ratio hypertension	2.206	0.3238	(4)
CAIDE odds ratio hyperlipidemia	1.879	0.3161	(4)
	Dementia Progression Annual Rate of MMSE Change		
Intercept	−5.4663	0.9836	(21)
PM1 [min(PrevMMSE, 9)]	−0.4299	0.0597	(21)
PM2 [max[0, min(PrevMMSE, –9, 9)]]	−0.0042	0.0410	(21)
PM3 [max[0, min(PrevMMSE, –18, 12)]]	0.1415	0.0487	(21)
Age at baseline	0.0747	0.0127	(21)
Previous rate of MMSE change	−0.0791	0.0317	(21)
	Dementia Mortality Parameters		
Hazard ratio dementia mortality 60–85 years	4.54	0.1276	(26)
Hazard ratio dementia mortality 75+ years	2.77	0.0784	(26)
	Dementia Costs		
Health care cost mild dementia (MMSE 21–26)	£3103	Assumed 10%	(23)
Health care cost moderate dementia (MMSE 10–20)	£8293	Assumed 10%	(23)
Health care cost severe dementia (MMSE 0–9)	£9841	Assumed 10%	(23)
Social care cost mild dementia (MMSE 21–26)	£5674	Assumed 10%	(23)
Social care cost moderate dementia (MMSE 10–20)	£22703	Assumed 10%	(23)
Social care cost severe dementia (MMSE 0–9)	£23466	Assumed 10%	(23)
	Dementia Utilities		
Utility decrement MMSE 21–25	0.93	Assumed 10%	(24)
Utility decrement MMSE 15–20	0.725	Assumed 10%	(24)
Utility decrement MMSE 10–14	0.710	Assumed 10%	(24)
Utility decrement MMSE 0–9	0.478	Assumed 10%	(24)

BMI, body mass index; CAIDE, Cardiovascular Risk Factors, Aging, and Incidence of Dementia; HDL, high-density lipoprotein; HSE, Health Survey for England; MMSE, Mini Mental State Examination; PHE, Public Health England; PM, Previous MMSE; THIN, The Health Improvement Network.

### Intervention Metabolic Effects

The NHS DPP consists of an intensive lifestyle management program aimed at those at high risk of diabetes due to impaired glucose regulation (IGR). IGR is defined as HbA1c 6% to 6.4% (42–47 mmol/mol) or fasting plasma glucose of 5.5 to 6.9 mmol/L. Evidence for the effectiveness of the program came from a Public Health England (PHE) commissioned evidence review and meta-analysis of pragmatic diabetes prevention interventions, which was used in previous work to evaluate the NHS DPP^18^ (see Table 1). The average cost of the DPP intervention from the PHE impact assessment of £270 per participant was used.^11^

The SPHR diabetes prevention model implements intervention effects through instantaneous alterations in metabolic trajectories in year 1 of the simulation, as most of the evidence is based on effects observed at intervals of 6 months/1 year. Beyond that, we assume that the gap between the intervention and control arm narrows over time. It was assumed that individuals return to the BMI/SBP/HbA1c/cholesterol level that they would have been without intervention after 5 years, consistent with the previous modeling assumptions for the National Institute for Care Excellence (NICE) guidelines for diabetes prevention (PH38)^19^ (Table 2).

**Table 2:** Comparison of Results with and without Dementia Included: Lifetime Cost Effectiveness of NHS DPP v. No Implementation of NHS DPP

	No Dementia		Dementia Only		Reduced Dementia Risk	
Absolute Results	No DPP	DPP	No DPP	DPP	No DPP	DPP
Net benefit (£20,000 willingness to pay)	186,525	186,525	180,904	181,771	180,904	182,074
Health and social care cost (per person)	26,870	26,725	30,134	30,013	30,134	29,967
Health care cost (per person)	25,032	24,909	25,604	25,493	25,604	25,511
Social care cost (per person)	1838	1816	4530	4520	4530	4455
Dementia cost (per person)	NA	NA	1172	1177	1172	1146
Cardiovascular cost (per person)	6550	6453	6361	6266	6361	6289
QALYs (per person)	10.67	10.71	10.55	10.59	10.55	10.60
Life years (per 1000 people)	24.17	24.24	23.74	23.80	23.74	23.84
Diabetes diagnoses (per 1000 people)	663	651	657	646	657	647
CVD events (per 1000 people)	456	453	446	443	446	445
Dementia diagnosis (per 1000 people)	231	232	242	243	242	238
			Targeting Strategy (Incremental Results v. Do Nothing)			
Incremental Results for DPP v. No DPP		No Dementia	Dementia Only	Difference from No Dementia	Reduced Dementia Risk	Difference from No Dementia
Incremental net benefit (£20,000 willingness to pay)		£1000	£987	–£13	£1290	£291
Incremental health and social care cost (per person)		–£145	–£121	£24	–£167	–£22
Incremental health care cost (per person)		–£123	–£111	£12	–£93	£30
Incremental social care cost (per person)		–£23	–£10	£13	–£75	–£52
Incremental dementia cost (per person)		0	£5	£5	–£26	–£26
Incremental cardiovascular cost (per person)		–£97	–£95	£2	–£72	£25
Incremental QALYs (per person)		0.0427	0.0433	0.0006	0.0562	0.0134
Incremental life years (per person)		0.0635	0.0665	0.0030	0.1003	0.0368
Incremental diabetes diagnoses (per 1000 people)		−11	−11	0	−10	1
Incremental CVD events (per 1000 people)		−3	−3	0	−1	1
Incremental dementia diagnosis (per 1000 people)		0	1	1	−4	−4

CVD, cardiovascular disease; DPP, diabetes prevention program; NHS, National Health Service; QALY, quality-adjusted life year.

In the control arm of the simulation, the eligible population did not receive any intervention to prevent diabetes and did not incur any additional costs beyond the usual monitoring and care described within the model.

### Adapting the Model to Include Dementia

The simulation was adapted to estimate the incidence of dementia for the population. We do not distinguish between dementia subtypes (e.g., Alzheimer, vascular) because categorizing dementia types was not indicated for an analysis that is aiming to estimate the burden of dementia in the population. An individual’s risk of dementia can be calculated from age 60 onward using 2 published risk scores for dementia diagnosis.^2^ The risk models were estimated from a sample of 930,395 people who were followed for 5 years in The Health Improvement Network (THIN) primary care data set, which characterizes current patterns of routine diagnosis.^2^ In the simulation, the risk scores are used to estimate the probability of dementia diagnosis conditional on the individual’s simulated risk factors such as socioeconomic status, smoking, gender, age, BMI, systolic blood pressure, lipids, antihypertensive, diabetes, depression, and history of stroke. Other risk factors, such as anxiety and nonsteroidal anti-inflammatory drugs (NSAIDs), were added to the simulated individual’s profile information in line with prevalence statistics reported in the THIN data. In each model cycle, the THIN risk scores generated a probability of dementia diagnosis for eligible individuals. The probabilities were sampled at random to determine dementia diagnosis. Severity and progression of dementia are modeled using the Mini Mental State Examination (MMSE). To generate heterogeneity in cognitive function at dementia diagnosis, a Gamma distribution was fitted to match summary data from the population-based Cognitive Function and Ageing Study data.^20^ The data were fitted to summary statistics, so cognitive function is not linked to age, sex, or health status. Cognitive decline is characterized by changes in MMSE score over time from a statistical analysis of the Consortium to Establish a Registry for Alzheimer’s Disease, developed for a pharmacoeconomic model for donepezil.^21,22^

Dementia diagnosis costs, ongoing health care, social care costs associated with dementia, and level of cognitive impairment were taken from a dementia costing study. This study provides a synthesis of best available evidence from trials and observational studies to estimate the current cost of dementia by severity level.^23^ HRQoL scores were attributed to cognitive impairment based on a study of utilities in Alzheimer disease,^24^ which was used in the most recent NICE Health Technology Assessment for Alzheimer disease,^25^ and no more recent studies were identified. The risk of all-cause mortality following dementia is increased based on estimates from 2 cohort studies,^26^ which included 2566 persons over 8 years. The hazard ratios were applied in the model to all-cause mortality to describe mortality at younger ages (60–84) and older ages (85+). Full details of the dementia modeling assumptions and parameters can be found in section 7 of the supplementary material. A summary of key parameters is presented in Table 1.

### Intervention Effects on Dementia Incidence

In the no-dementia scenario, the intervention has no effect on dementia incidence. In the dementia-only scenario, dementia incidence is modified only through indirect reductions in diabetes and stroke incidence. In the reduced dementia risk scenario, changes in BMI, blood pressure, and cholesterol are assumed to reduce the incidence of dementia for 20 years after initiating the intervention based on evidence from the CAIDE Dementia Risk Score.^4^

The CAIDE risk score predicts the risk of dementia based on vascular, behavioral, and demographic risk factors during a 20-year follow-up of 1409 individuals. We estimate a relative risk reduction for the intervention based on the observed changes in BMI, systolic blood pressure, and cholesterol and the odds ratios from the CAIDE risk score. The relative risk is calculated based on the magnitude and duration of changes in metabolic risk factors to reflect the temporary intervention effect on dementia risk. This relative risk is applied to the probability of dementia estimated by the THIN risk scores in the model. Full details of how the relative risk of dementia associated with the intervention has been calculated can be found in section 7 of the supplementary material.

### Estimating Costs and QALYs

Costs were estimated from an NHS and personal social services (PSS) perspective in 2016–2017 UK pounds. Health care and social care costs were assigned to all the health outcomes simulated in the model to estimate an overall cost for each individual in the model. Costs accumulate additively in the model, so there is a risk of double counting some aspects of health care resource use. However, the extent of overlapping costs is likely to be small given that the costs of diabetes describe mainly treatment costs and glucose monitoring. CVD is mostly driven by the cost of acute hospitalization and outpatient visits, while the costs of cancer are driven by treatment costs, renal failure by the cost of dialysis, and osteoarthritis from the costs of replacement surgery. In contrast, the costs of dementia are driven by residential and community services.

At baseline, EQ-5D scores were extracted from the HSE data set to describe an individual’s HRQoL, and a utility decrement for age was applied to the baseline EQ-5D each year.^27^ CVD, cancer, microvascular disease, osteoarthritis, cognitive impairment (MMSE score), and depression were associated with a utility factor decrement that was multiplied by the individual’s utility, adjusted for age. HRQoL decrements were applied multiplicatively to avoid double counting of HRQoL loss due to multimorbidity.^28^ Details of how costs and utilities were estimated and how they were used in the model are detailed in sections 8 to 10 of the supplementary material.

### Outcomes

Lifetime costs and QALYs were discounted at 3.5% per annum. We estimated the overall incremental monetary benefit of the interventions per person by assuming a willingness to pay (λ) of £20,000 per QALY. Net benefit values above zero are cost-effective, with higher values being more cost-effective than lower values.

IncrementalNetBenefit=λ(inc.QALY)−(inc.Cost)

The model also allowed us to estimate the incremental change in diabetes, CVD, and dementia diagnoses.

### Scenario Analysis

The NHS DPP v. no implementation is evaluated in 3 main scenarios. The no-dementia scenario provides a baseline model in which the model has been updated from previous versions but where dementia is not modeled. The dementia-only scenario includes the THIN dementia risk model, dementia diagnosis, MMSE progression, mortality risk, costs, and health-related quality of life decrements as described earlier. The reduced dementia risk model includes all aspects of the dementia-only model, plus a dementia risk reduction associated with modification of lifestyle risk factors using the coefficients from the CAIDE risk score.

Additional stratified analyses investigated the impact of population characteristics on the net benefit across subgroups of the population. This analysis aimed to investigate whether there were particular populations where including dementia would have a greater impact on cost-effectiveness outcomes. For these additional analyses, the cohort was stratified by age and diabetes risk (HbA1c 6.0%–6.1%, HbA1c 6.2%–6.4%).

### Sensitivity Analyses

Several sensitivity analyses were conducted to explore the impact of dementia parameters on the health economic outcomes. A full list of sensitivity analyses conducted can be found in the supplementary material. To investigate parameter uncertainty, 5000 probabilistic sensitivity analysis (PSA) samples were run for individuals in the eligible population.

## Results

The absolute costs and health benefits for all scenarios are reported in Table 2. Including dementia as an outcome increases the total lifetime health and social care costs in the dementia-only and reduced dementia risk scenarios. Incorporating dementia diagnosis and dementia-related mortality leads to an overall reduction in number of diabetes diagnoses and cardiovascular events and fewer lifetime QALYs gained.

The incremental costs and health benefits for the DPP compared with a do-nothing intervention are reported in Table 2. The dementia-only analysis shows that including dementia, with no direct intervention effect, has a modest effect on the incremental analysis. The additional costs of extending life expectancy outweigh the benefits of reduced dementia incidence via stroke and diabetes. The intervention remains cost saving, but with less cost savings across health and social care and fewer QALY gains. In the reduced dementia risk scenario, where the CAIDE risk score is used to estimate a direct intervention effect on the incidence of dementia, the overall net benefit is greater than the no-dementia scenario. Additional cost savings are observed in social care and dementia costs, but these cost savings are mitigated by less cost savings related to cardiovascular events.

**Figure 1:**
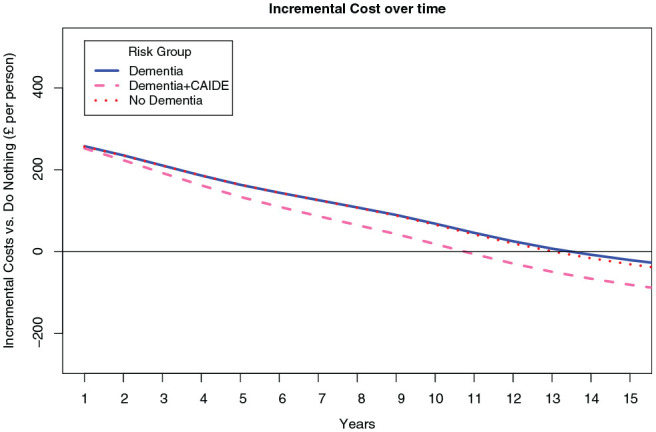
Comparison of results with and without dementia included. Cumulative incremental costs v. do nothing for first 20 years in 3 modeled scenarios. CAIDE, Cardiovascular Risk Factors, Aging, and Incidence of Dementia.

Figure 1 illustrates how incremental costs are recouped in the short term. In the no-dementia scenario, it is estimated to take around 12 years for the NHS DPP to become cost saving. The dementia-only scenario increased the time slightly to deliver cost saving but not above 12 years. In the reduced dementia risk scenario, where the intervention is assumed to have a direct effect on dementia risk using the CAIDE risk score, the time to cost savings is reduced to 10 years.

Stratified analyses explored the impact of population age and diabetes risk on the incremental costs and QALYs (Table 3). In the dementia-only scenario, the net benefit decreased in all subpopulations, but the decrease was larger in a younger cohort and lower diabetes risk. In the reduced dementia risk scenario, there is a clear trend to increasing net benefit in older populations.

**Table 3:** Incremental and Net Benefit Results for Subgroup Analyses

	Incremental Costs			Incremental QALYs			Net Benefit		
Characteristic	No Dementia	Dementia Only	Reduced Dementia Risk	No Dementia	Dementia Only	Reduced Dementia Risk	No Dementia	Dementia Only	Reduced Dementia Risk
Base case analysis	–£145	–£121	–£167	0.0427	0.0433	0.0562	£1000	£987	£1290
Target age 40–50	–£296	–£282	–£286	0.0354	0.0332	0.0370	£1004	£947	£1026
Target age 50–60	–£271	–£267	–£273	0.0414	0.0406	0.0483	£1099	£1079	£1239
Target age 60–70	–£174	£47	–£164	0.0613	0.0612	0.081	£1399	£1354	£1783
Target age 70–80	–£2	£–131	–£67	0.0581	0.0581	0.089	£1165	£1116	£1842
High risk of diabetes (HbA1c 6.2%–6.4%)	–£239	–£214	–£255	0.0516	0.0513	0.0629	£1271	£1240	£1513
Low risk of diabetes (HbA1c 6.0%–6.1%)	–£34	–£39	–£84	0.0411	0.0350	0.0473	£856	£738	£1030

QALY, quality-adjusted life year.

**Figure 2:**
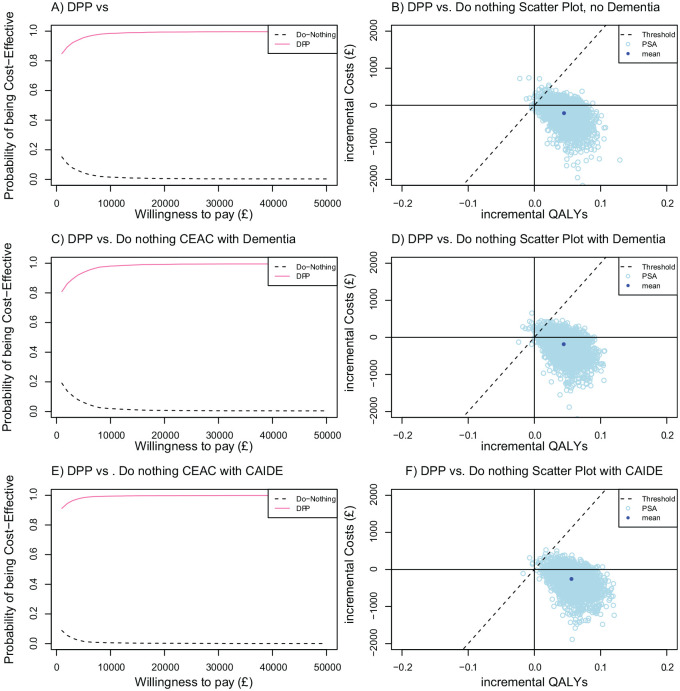
Probabilistic sensitivity analysis for 3 simulated scenarios. CAIDE, Cardiovascular Risk Factors, Aging, and Incidence of Dementia; CEAC, Cost-effectiveness Acceptability Curve; DPP, diabetes prevention program; QALY, quality-adjusted life year.

The sensitivity analysis found a reasonable degree of stability in the model outcomes to dementia-related parameters (Suppl. Table S53). The dementia-related parameters, including dementia incidence, associations between diabetes, stroke and dementia, dementia costs, utilities, mortality risk, and MMSE score progression, did not substantially affect health economic outcomes. The relative risk parameter based on the CAIDE risk score had a notable impact on the net benefit in the reduced dementia risk scenario. The probabilistic sensitivity analysis indicates that there is a 98% probability that the DPP is cost-effective with dementia as a health outcome across all scenarios (Figure 2).

## Discussion

The study shows that explicitly modeling dementia as a health outcome in the dementia-only scenario reduced the net benefit of the intervention compared with equivalent analyses that did not include dementia. While the indirect treatment effects via diabetes and stroke increased cost savings, this effect was canceled out by increased health care costs in additional years gained. Including dementia as an outcome did not substantially affect the incremental QALYs in this population, suggesting that there are marginal QALY gains from delayed onset of dementia and additional life years gained in this context.

Guidelines for the development of cost-effectiveness analyses for public health interventions recommend that disease outcomes that do not affect the cost-effectiveness outcomes can be excluded from model structures.^29^ In this study, including dementia did not alter the recommendations for the intervention or substantially affect the health economic outcomes. As such, these analyses did not provide a compelling argument to routinely include dementia in public health models for lifestyle interventions. One-way sensitivity analyses indicate that the net benefit was not affected by changes to the natural history of dementia, costs, or utilities, so this finding is likely to be similar in other health care settings. Nevertheless, the analyses have highlighted some situations where the inclusion of dementia may be influential.

The model was sensitive to the inclusion of a direct intervention effect on dementia incidence. The reduced dementia risk scenario and parameter sensitivity analyses indicated that there were substantial cost savings and QALY gains from reducing the incidence of dementia. Therefore, inclusion of dementia as an outcome would be justified for interventions with large and long-lasting effects on modifiable risk factors that are included in the CAIDE score, including education and physical activity. These intervention effects were not included in this example. Also, the direct intervention effects had the greatest impact in older populations. Reducing dementia risk in older populations was more valuable in this model, where discount rates reduced cost and QALY gains in younger populations whose major events occur further into the future.

Conversely, in the dementia-only scenario, there is a modest risk that the cost savings and QALY gains would be overstated if dementia were not included in the model. This impact is greatest in younger populations and those at low risk of diabetes. Dementia moderated the health economic outcomes of public health interventions, particularly where individuals were at a lower risk of health complications and the health gains were accrued later in life. This may be because younger populations will benefit less from the indirect effects of reduced dementia incidence through reduced stroke and diabetes. This finding is most likely to affect health economic evaluations of population-wide, upstream interventions rather than interventions targeting at smaller at-risk groups.

Few studies have included the costs of dementia in an economic evaluation of lifestyle interventions targeting diabetes and cardiovascular disease. Two studies focusing on interventions to increase physical activity included dementia as a health outcome.^30,31^ Increased physical activity increased life expectancy and resulted in decreased spending overall on health and social care, even after accounting for additional spending during life years gained. Both studies assumed a direct relationship between physical activity and dementia, indicating health gains/cost savings from reduced dementia incidence.

Public health interventions often affect multiple risk factors and multiple disease outcomes. This is the first economic evaluation to conduct a comprehensive evaluation of the effects of a public health intervention by capturing the health gains and costs across diabetes, cardiovascular disease, cancer, osteoarthritis, dementia, and depression.^32^ We have demonstrated that the additional burden of diseases, such as dementia in later life, does not substantially affect the cost-effectiveness of a large-scale public health intervention like the NHS DPP. This finding will help guide the process of conceptualizing and deciding the model boundary for future noncommunicable disease models.

A key consideration when including dementia in a model for public health interventions is how much detail on dementia progression is necessary. In this model, we allow for variability in disease severity at diagnosis, heterogeneity in MMSE score progression, and variation in costs and HRQoL decrements across disease progression states. This framework is more complex than previous public health dementia models that specify dementia as a single disease state. However, our approach is less complex than cost-effectiveness models developed for Alzheimer disease treatments, where domains for function and behavior are used to capture the broad nature of Alzheimer disease and the benefits from treatment. Including severity scales for behavioral and functional abilities such as the Neuropsychiatric Inventory (NPI), activities of daily living (ADLs), and instrumental ADLs (IADL) would provide a more comprehensive description of dementia and a broader range of outcomes. Getsios et al.^21^ estimated disease progression scores incorporating correlation between these measures. There is no evidence from DPP trials to suggest that the intervention affects behavioral and functional abilities, and there is limited evidence on the impact of these factors on costs and mortality. Excluding these measures is unlikely to affect this evaluation. The complexity of dementia progression must be weighed against the computational burden of a multidisease microsimulation as it is not feasible to model all diseases to the same standard as single-disease models. The MMSE domain progression structure allows the model to capture the most important impacts of the timing of dementia onset through deterioration in QALYs and increases in costs over time. However, this modeling framework is not sufficient to evaluate the benefits of treatment postdiagnosis or preventative interventions that affect patient functioning at baseline.

The study is limited by the data available to describe the effectiveness of the diabetes/CVD intervention in reducing dementia incidence. In these analyses, we assume that the maintenance of changes in metabolic risk factor reduction is 5 years, and in the reduced dementia risk model, risk of dementia is reduced for 20 years. There is considerable uncertainty in whether short-term changes in risk factors can modify the risk of dementia and how this relationship varies by age, duration, and magnitude of change. We have based our assumptions on data from an influential observational study.^4^ However, 3 large multidomain trials (FINGER, MAPT, and PreDIVA) have been completed in recent years. The FINGER trial showed that a multidomain lifestyle intervention can provide a marginal benefit for cognition over a nonactive intervention in elderly people with an elevated risk of dementia.^33^ The primary results from the other trials did not show a statistically significant benefit of preventive interventions,^6,7^ although subgroup analyses of untreated hypertension showed potential beneficial effects of intervention on dementia incidence.^7^ Overall, results from these 3 trials suggest that targeting of preventive interventions to at-risk individuals is an effective strategy, if limited in impact. However, there is a need for more evidence from randomized controlled trials to demonstrate whether modifications to metabolic risk factors result in reductions in dementia incidence.

There is also considerable uncertainty in the relationships between lifestyle interventions and dementia, and careful consideration was given to characterizing treatment benefit in the reduced dementia risk scenario. However, the study was limited by evidence to describe the characteristics of the treatment effect. The dementia-only and reduced dementia risk scenarios demonstrate the impact of dementia risk assumptions, from an indirect effect from stroke and diabetes to an immediate risk reduction for 20 years, on cost-effectiveness outcomes. These scenarios provide an explicit range of cost-effectiveness results, and the sensitivity analysis highlights the importance of the risk reduction parameter to the net benefit. Within this boundary, the true effect may be characterized by time lags, effects conditional on age or deterioration in effect over time. However, modifications to these assumptions without a better understanding of the mechanisms between lifestyle and dementia risk would be arbitrary. There is a paucity of data on the effect of changes in metabolic risk factors on the incidence of dementia, and further research is needed to explore the relationships between lifestyle and dementia risk across the life course.

## Conclusions

The study demonstrates that it is feasible to include dementia as an outcome in public health modeling studies of lifestyle interventions. The study provides a framework to model dementia alongside diabetes and cardiovascular diseases. The inclusion of outcomes for dementia moderates the cost savings associated with the NHS DPP. However, there are gains in cost savings and QALYs if the intervention directly reduces the incidence of dementia. The addition of dementia in our model did not alter the recommendation that the NHS DPP is a cost-effective use of resources.

## Supplemental Material

File_S1_-_Supplementary_Appendix_with_Dementia.rjf_online_supp – Supplemental material for The Impact of Including Costs and Outcomes of Dementia in a Health Economic Model to Evaluate Lifestyle Interventions to Prevent Diabetes and Cardiovascular Disease Click here for additional data file. Supplemental material, File_S1_-_Supplementary_Appendix_with_Dementia.rjf_online_supp for The Impact of Including Costs and Outcomes of Dementia in a Health Economic Model to Evaluate Lifestyle Interventions to Prevent Diabetes and Cardiovascular Disease by Penny Breeze, Chloe Thomas, Praveen Thokala, Louise Lafortune, Carol Brayne and Alan Brennan in Medical Decision Making
