# Supplementary material for: The Impact of Including Costs and Outcomes of Dementia in a Health Economic Model to Evaluate Lifestyle Interventions to Prevent Diabetes and Cardiovascular Disease
Source: Med Decis Making. 2020 Sep 19;40(7):912–23. doi: 10.1177/0272989X20946758 (PMC7583453; doi:10.1177/0272989X20946758)
Supplement: File_S1_-_Supplementary_Appendix_with_Dementia.rjf_online_supp – Supplemental material for The Impact of Including Costs and Outcomes of Dementia in a Health Economic Model to Evaluate Lifestyle Interventions to Prevent Diabetes and Cardiovascular Disease [file File_S1_-_Supplementary_Appendix_with_Dementia.rjf_online_supp.docx]

File S1: Supplementary Methods

Contents

[2. Developing the Conceptual Model 4](#_Toc476661247)

[3. Model Structure 5](#_Toc476661248)

[4. Baseline Population Characteristics: Health Survey for England 9](#_Toc476661249)

[Exclusion Criteria 10](#_Toc476661250)

[HSE 2014 Missing data imputation 14](#_Toc476661251)

[5. GP Attendance in the General Population 16](#_Toc476661252)

[6. Longitudinal Trajectories of Metabolic Risk Factors 18](#_Toc476661253)

[Whitehall II Data Analysis 18](#_Toc476661254)

[ELSA Data Analysis 22](#_Toc476661255)

[BMI Trajectory 23](#_Toc476661256)

[Glycaemic Trajectory in Non-Diabetics/undiagnosed Diabetes 23](#_Toc476661257)

[HbA1c trajectory in type 2 diagnosed diabetics 23](#_Toc476661258)

[Total Cholesterol and HDL Cholesterol Trajectories in Individuals not receiving Statins 25](#_Toc476661259)

[Total Cholesterol and HDL Cholesterol Trajectories in Individuals receiving Statins 25](#_Toc476661260)

[SBP Trajectories in Individuals Not receiving Anti-hypertensive treatment 27](#_Toc476661261)

[SBP Trajectories in Individuals receiving anti-hypertensive treatment 27](#_Toc476661262)

[Metabolic Risk factor screening 28](#_Toc476661263)

[Diagnosis and Treatment Initiation 29](#_Toc476661264)

[7. Comorbid Outcomes and Mortality 29](#_Toc476661265)

[Cardiovascular Disease 29](#_Toc476661266)

[First Cardiovascular event 29](#_Toc476661267)

[Subsequent Cardiovascular events 33](#_Toc476661268)

[Congestive Heart Failure 34](#_Toc476661269)

[Microvascular Complications 36](#_Toc476661270)

[Cancer 40](#_Toc476661271)

[Breast cancer 41](#_Toc476661272)

[Colorectal cancer 41](#_Toc476661273)

[Osteoarthritis 43](#_Toc476661274)

[Depression 43](#_Toc476661275)

[Dementia 45](#_Toc476661276)

[Dementia Diagnosis 47](#_Toc476661277)

[Disease Progression 47](#_Toc476661278)

[Mortality 48](#_Toc476661279)

[Cardiovascular Mortality 48](#_Toc476661280)

[Cancer Mortality 48](#_Toc476661281)

[Other cause Mortality (including diabetes and Dementia risk) 49](#_Toc476661282)

[8. Direct Health Care Costs 51](#_Toc476661283)

[GP attendance 52](#_Toc476661284)

[Diabetes 52](#_Toc476661285)

[Metformin Monotherapy 53](#_Toc476661286)

[Metformin plus Gliptins 54](#_Toc476661287)

[Insulin plus Oral Anti-diabetics 55](#_Toc476661288)

[Statins 56](#_Toc476661289)

[Anti-hypertensives 56](#_Toc476661290)

[Cardiovascular Events 57](#_Toc476661291)

[Renal Failure 58](#_Toc476661292)

[Foot Ulcers 59](#_Toc476661293)

[Amputation 59](#_Toc476661294)

[Blindness 59](#_Toc476661295)

[Cancer 60](#_Toc476661296)

[Osteoarthritis 61](#_Toc476661297)

[Depression 61](#_Toc476661298)

[Dementia 62](#_Toc476661299)

[Cost of Diagnosis 62](#_Toc476661300)

[Ongoing healthcare costs 62](#_Toc476661301)

[9. Social Care costs 63](#_Toc476661302)

[Stroke 63](#_Toc476661303)

[Dementia 63](#_Toc476661304)

[10. Utilities 64](#_Toc476661305)

[Baseline Utility 64](#_Toc476661306)

[Utility Decrements 64](#_Toc476661307)

[11. Intervention Effectiveness 68](#_Toc476661308)

[Intervention Effectiveness 68](#_Toc476661309)

[Intervention Costs 70](#_Toc476661310)

[Duration of Intervention Effect 71](#_Toc476661311)

[The CAIDE risk score for dementia 71](#_Toc476661312)

[Modifying the CAIDE Risk Score 72](#_Toc476661313)

[Estimating the Effect of the Intervention on Dementia 74](#_Toc476661314)

[12. Probabilistic Sensitivity Analysis 76](#_Toc476661315)

[13. Model Validation 77](#_Toc476661316)

[Metabolic trajectories 78](#_Toc476661317)

[Short term Dementia Incidence 79](#_Toc476661318)

[Long-Term Outcomes 81](#_Toc476661319)

[Life expectancy 81](#_Toc476661320)

[14. References 81](#_Toc476661321)

# Developing the Conceptual Model

The conceptual model was developed according to a new conceptual modeling framework for complex public health models (1). In line with this framework the conceptual model was developed in collaboration with a project stakeholder group comprising health economists, public health specialists, research collaborators from other SPHR groups, diabetologists, local commissioners and lay members. The initial broad scope for the conceptual model was based on the structure of previous diabetes prevention models used for National Institute for Health and Care Excellence public health guidance (2;3) and discussions with experts in diabetes prevention modeling. The model was further extended to include Dementia as a possible health outcome for individuals aged over 60 years in the model.

# Model Structure

We developed an individual patient simulation that estimates individuals’ health in yearly cycles until death. The simulation draws baseline demographic and clinical status for individuals sampled from the Health Survey for England (HSE) 2014 (4). The simulation estimates yearly changes in metabolic risk factors based upon the individuals’ baseline characteristics. Within each annual cycle the individuals may be screened for hypertension, dyslipidaemia or diabetes during a visit to the General Practitioner (GP). Opportunistic screening is used to determine diabetes diagnosis or the initiation of anti-hypertensive treatment or statins. Baseline characteristics and metabolic risk factors determine the individuals’ probability of cardiovascular events, diabetes microvascular complications, cancer, osteoarthritis and depression. Individuals within the model may die in any cycle as a result of cardiovascular disease, cancer or from other causes.

Figure 1 illustrates the sequence of updating clinical characteristics and clinical events that are estimated within a cycle of the model. This sequence is repeated for every annual cycle of the model.

The first stage of the sequence updates the age of the individual. The second stage estimates how many times the individual attends the GP. The third stage estimates the change in BMI of the individual from the previous period. In the fourth stage, if the individual has not been diagnosed as diabetic (Diabetes_Dx=0) their change in glycaemia is estimated using the Whitehall II model. If they are diabetic (Diabetes_Dx=1), it is estimated using the UKPDS model. In stages five and six the individual’s blood pressure and cholesterol are updated using the Whitehall II model if the individual is not identified as hypertensive or receiving statins. In stage seven, the individual may undergo assessment for diabetes, hypertension and dyslipidaemia during a GP consultation. From stage eight onwards the individual may experience cardiovascular outcomes, diabetes related complications, cancer, osteoarthritis, depression, dementia, and updated cognitive decline associated with dementia diagnosis. If the individual has a history of cardiovascular disease (CVD history=1), they follow a different pathway in stage eight to those without a history of cardiovascular disease (CVD history=0). Individuals with HbA1c greater than 48 mmol/mol (6.5%) are assumed to be at risk of diabetes related complications. Individuals who do not have a history of cancer (Cancer history=0) are at risk of cancer diagnosis, whereas those with a diagnosis of cancer (Cancer history=1) are at risk of mortality due to cancer. Individuals without a history of osteoarthritis or depression may develop these conditions in stages 12 and 13. In stage 14 individuals with dementia have their cognitive status updated and those individuals aged over 60 without a diagnosis of dementia may receive a diagnosis. Finally, all individuals are at risk of dying due non cardiovascular or cancer mortality. Death from renal disease is included in the estimate of other-cause mortality.

The modeling structure and cycle sequence is explained in more detail using a hypothetical patient below:

Consider a white male aged 53 sampled from the baseline population, referred to hereafter as Mr X. Mr X has a series of baseline demographics informed by the baseline population dataset, or imputation if missing. These characteristics influence his future health outcomes in the model. In the first cycle of the model the age of Mr X is 53. In this cycle Mr X’s attendance at the GP is generated and recorded within the model dependent upon his age and gender. In the first cycle of the model Mr X’s BMI is extracted from his baseline data. The effect of an intervention on BMI in the first 12 months is applied here if required. Similarly baseline values for HbA1c, systolic blood pressure (SBP), total cholesterol, and HDL cholesterol are extracted from his baseline dataset and modified for treatment effect if necessary. If Mr X has attended the GP in this cycle he may receive opportunistic screening for diabetes, hypertension or high cardiovascular risk if he meets certain risk criteria, agreed by the stakeholder group. If he is diagnosed with any of these conditions/risks, treatments are initiated according to current guidelines in the UK for diabetes diagnosis, anti-hypertensive treatment and statin treatment. If Mr X receives any of these treatments his HbA1c, SBP and/or total cholesterol are reduced accordingly.

Having established Mr X’s metabolic risk profile the model determines if Mr X experiences any major health events in this first cycle. If Mr X does not have a history of cardiovascular disease (CVD) the model estimates the probability that he has a fatal or non-fatal cardiovascular event in this cycle. The event is determined using a Bernoulli trial. If Mr X has a history of CVD his probability of a progressing to unstable angina, MI, stroke or a fatal event is determined. If Mr X has HbA1c greater than 48 mmol/mol (6.5%) or a diagnosis of diabetes, the probability of foot ulcer, renal disease, amputation and blindness are calculated and evaluated using a Bernoulli trial. If Mr X does not have diabetes he is not at risk of these complications in this cycle.

In the next stage of the cycle Mr X may develop breast or colon cancer if he has not already got a history of cancer. The probability of these complications is generated and evaluated in a Bernoulli trial. If Mr X has a history of cancer, he is at risk of mortality due to cancer in this cycle. If Mr X does not have osteoarthritis the probability of developing this complication is evaluated in this cycle and a diagnosis is given according to a Bernoulli trial. If Mr X has a diagnosis of osteoarthritis his health status for this complication remains unchanged. Similarly, if Mr X does not have depression the probability of developing it is evaluated in this cycle and a diagnosis is given according to a Bernoulli trial. If Mr X has a diagnosis of depression his health status for this complication remains unchanged. If Mr X is over 60 years old and does not have a diagnosis of dementia, he may receive a new diagnosis according to a Bernoulli trial using his current probability of a dementia diagnosis. If Mr X has an existing diagnosis of dementia his MMSE score will be updated to reflect any deterioration in memory and disease severity.

Finally, assuming Mr X has not experienced a fatal event due to CVD or cancer, the probability of death is calculated and evaluated in a Bernoulli trial based on Office of National Statistics life tables combined with hazard ratios for dementia and diagnosis (5). If Mr X remains alive he proceeds to the next cycle. If Mr X dies his health status, costs and QALYs are stored and he is removed from the model.

In the second and subsequent cycles, the model proceeds through a similar sequence of events. However, Mr X firstly ages by the cycle length of one year. A new number of GP visits within the cycle is generated. BMI will increase or decrease according to a trajectory assigned to Mr X at baseline, and intervention effect maintenance if relevant. Similarly, HbA1c, SBP, total cholesterol, and HDL cholesterol all change in this period on a prespecified trajectory and intervention effect. Mr X may undergo opportunistic screening as specified in year one. The sequence of evaluations to determine health events and complications experienced by Mr X in this cycle is the same as described above, however Mr X’s metabolic risk factors, treatments, and history are updated with the changes described above.

Figure 1: Model Schematic

2. GP visits

.

3.

BMI

.

4.a. Glucose

.

*Diabetes_Dx*

*=1*

*Diabetes_Dx*

*=0*

1

. Age

4.a. HbA1c+treatment

.

5.a. Blood pressure

.

5.b. Blood pressure

.

*Hypertenson*

*=0*

*Hypertenson*

*=1*

6.a. Cholesterol

6.a. Cholesterol

*Statin=0*

*Statin=1*

7. Screening

.

8.a. CVD events

8.b. CVD events

*CVD history=0*

*CVD history=1*

9. CVD

Mortality

10. Renal failure, ulcer,

amputation and blind

*HbA*

*<6.5*

*HBA>6.5*

11.a. Cancer events

11.b. Cancer events

*Cancer history=0*

*Cancer history=1*

11.b. Cancer

Mortality

*Osteo*

*history=1*

12.

Osteo

events

*Osteo*

*history=0*

13. Depression

*Depression=0*

*Depression=1*

15. All cause

mortality

*Dementia=0*

*Dementia=1*

14.b Update MMSE

14.a Dementia

# Baseline Population Characteristics: Health Survey for England

The model required demographic, anthropometric and metabolic characteristics that would be representative of the UK general population. The Heath Survey for England (HSE) was suggested by the stakeholder group because it collects up-to-date cross-sectional data on the characteristics of all ages of the English population. It also benefits from being a reasonably good representation of the socioeconomic profile of England. A major advantage of this dataset is that includes important clinical risk factors such as HbA1c, SBP, and cholesterol. The characteristics of individuals included in the cost-effectiveness model were based sampled from the HSE 2014 dataset (4). The whole dataset was obtained from the UK Data Service.

## Exclusion Criteria

The total sample size of the HSE 2014 was 10,080. Individuals from the HSE dataset who were younger than 16 years (N=2003) were excluded from the sample. This left a final sample size of 8077 individuals.

Summary statistics for the data extracted from the HSE2014 dataset are reported in Table 1.

Table 1: Characteristics of final sample from HSE 2014 (N=8077)

| Variable name (description) | Mean | Median | SD | Missing (N) |
| --- | --- | --- | --- | --- |
| Age | 50.02 | 49.00 | 18.63 | 0 |
| Income | 33810 | 24700 | 29246 | 1567 |
| Weight | 77.61 | 75.80 | 17.26 | 990 |
| Height | 167.7 | 167.3 | 9.74 | 938 |
| BMI | 27.52 | 26.66 | 5.48 | 1132 |
| Waist | 93.10 | 92.55 | 14.39 | 2818 |
| Hip | 105.6 | 104.1 | 15.95 | 2813 |
| Waist-Hip ratio | 0.881 | 0.879 | 0.092 | 2832 |
| Total Cholesterol | 5.194 | 5.1 | 1.104 | 4176 |
| HDL Cholesterol | 1.545 | 1.5 | 0.452 | 4175 |
| HbA1c | 5.615 | 5.4 | 0.785 | 4183 |
| SBP | 126.2 | 124.5 | 17.22 | 3208 |
| DBP | 72.75 | 72.5 | 11.07 | 3408 |
| Total units alcohol | 12.6 | 6.04 | 21.55 | 177 |
| Minute vigorous exercise | 65.45 | 30.0 | 107.66 | 1170 |
| Minutes walking | 89.45 | 60.00 | 114.86 | 1238 |
| EQ-5D | 0.8767 | 1 | 0.189 | 187 |

Table 2: Summary data for categorical (N=8077)

| Variable name (description) | Category | N | % |
| --- | --- | --- | --- |
| Sex | Male | 3588 | 44.4% |
|  | Female | 4489 | 55.6% |
|  | Missing | 0 | 0% |
| Economic Activity | In employment | 4334 | 53.7% |
|  | ILO unemployment | 315 | 3.8% |
|  | Retired | 2140 | 26.5% |
|  | Other Inactive | 1263 | 15.6% |
|  | Missing | 25 | 0.3% |
| Origin | White British | 6653 | 82.4% |
|  | White Irish | 76 | 0.9% |
|  | White other | 421 | 5% |
|  | White and Black Caribbean | 32 | 0.3% |
|  | White and Black African | 11 | 0.1% |
|  | White and Asian | 17 | 0.2% |
|  | Other mixed | 44 | 0.5% |
|  | Indian | 198 | 2% |
|  | Pakistani | 146 | 2% |
|  | Bangladeshi | 47 | 0.6% |
|  | Chinese | 44 | 0.5% |
|  | Other Asian | 91 | 1% |
|  | African | 105 | 1.3% |
|  | Caribbean | 73 | 0.9% |
|  | Other Black | 18 | 0.2% |
|  | Arab | 25 | 0.3% |
|  | Other | 44 | 0.5% |
|  | Missing | 32 | 0.4% |
| Urban | Urban | 3324 | 41.2% |
|  | Town | 273 | 3.4% |
|  | Village | 4480 | 55.5% |
| QIMD | 0.53-8.49 (least deprived) | 1777 | 22% |
|  | 8.49-13.79 | 1611 | 20.0% |
|  | 13.79-21.35 | 1557 | 19.3% |
|  | 21.35-34.17 | 1602 | 19.8% |
|  | 34.17-87.80 (most deprived) | 1530 | 18.9% |
| Smoking group | Current | 1444 | 17.9% |
|  | Ex-smoker | 2033 | 25.2% |
|  | Never smoke | 4535 | 56.1% |
|  | Missing | 65 | 0.8% |
| Smoking level | Low smoker | 534 | 6.6% |
|  | Moderate smoker | 627 | 7.8% |
|  | Heavy smoker | 276 | 3.4% |
|  | Don’t know | 7 | 0.1% |
|  | Non-smoker | 6570 | 81.3% |
|  | Missing | 63 | 0.8% |
| Hypertensive treatment | Yes | 1492 | 18.5% |
|  | No | 478 | 5.9% |
|  | Missing | 6107 | 75.6% |
| Statins | No | 4545 | 56.3% |
|  | Yes | 946 | 11.7% |
|  | Missing | 2586 | 32.0% |
| Long term illness 1 | 15:Stroke | 32 | 0.4% |
|  | 16:Heart attack/angina | 67 | 0.8% |
|  | 17:Other heart | 148 | 2% |
|  | 34:Arthritis/rheumatism | 396 | 4.9% |
|  | Missing | 4683 | 58.0% |
| Long term illness 2 | 15:Stroke | 14 | 0.2% |
|  | 16:Heart attack/angina | 56 | 0.7% |
|  | 17:Other heart | 88 | 1.1% |
|  | 34:Arthritis/rheumatism | 166 | 2% |
|  | Missing | 6275 | 77.7% |
| Long term illness 3 | 15:Stroke | 13 | 0.2% |
|  | 16:Heart attack/angina | 8 | 0.1% |
|  | 17:Other heart | 30 | 0.4% |
|  | 34:Arthritis/rheumatism | 89 | 1.1% |
|  | Missing | 7176 | 88.8% |
| Long term illness 4 | 15:Stroke | 6 | 0.1% |
|  | 16:Heart attack/angina | 8 | 0.1% |
|  | 17:Other heart | 13 | 0.2% |
|  | 34:Arthritis/rheumatism | 42 | 0.5% |
|  | Missing | 7623 | 94.4% |
| Long term illness 5 | 15:Stroke | 8 | 0.1% |
|  | 16:Heart attack/angina | 3 | 0.04% |
|  | 17:Other heart | 13 | 0.2% |
|  | 34:Arthritis/rheumatism | 15 | 0.2% |
|  | Missing | 7860 | 97.3% |
| Long term illness 6 | 15:Stroke | 1 | 0.01% |
|  | 16:Heart attack/angina | 2 | 0.02% |
|  | 17:Other heart | 2 | 0.02% |
|  | 34:Arthritis/rheumatism | 13 | 0.2% |
|  | Missing | 7975 | 98.7% |
| Diabetes | Yes | 548 | 6.7% |
|  | No | 7525 | 93.2% |
|  | Missing | 4 | 0.05% |
| Depression | Yes self reported diagnosis | 1107 | 13.7% |
|  | Yes not self reported diagnosis | 401 | 5.0% |
|  | No | 3977 | 49.2% |
|  | Missing | 2592 | 32.1% |
| Dementia | Yes self reported diagnosis | 11 | 0.1% |
|  | Yes not self reported diagnosis | 7 | 0.1% |
|  | No | 5467 | 67.7% |
|  | Missing | 2592 | 32.1% |
| Alcohol Problem | Yes | 67 | 0.8% |
|  | No | 61 | 0.8 % |
|  | Missing | 7949 | 98% |

A complete dataset was required for all individuals at baseline. However, no measurements for Fasting Plasma Glucose (FPG) or 2 hour glucose were obtained for the HSE 2014 cohort. In addition, the questionnaire did not collect information about individual family history of diabetes or family history of CVD. These variables were imputed from the Whitehall II dataset (see below) (5;6).

Many individuals were lacking responses to some questions but had data for others. One way of dealing with this was to exclude all individuals with incomplete data from the sample. However, this would have reduced the sample size and representativeness dramatically, which would have been detrimental to the analysis. It was decided that it would be better to make use of all the data available to represent a broad range of individuals within the UK population. With this in mind, we decided to use assumptions and imputation models to estimate missing data.

From this population individuals with a HbA1c above 6% were selected according to the criteria for the Diabetes Prevention Programme. The characteristics of the eligible population entering the model are summarized in Table 3.

Table 3 Diabetes Prevention Programme eligible population from Health Survey for England 2014 (N=2,329) with imputation of missing values

|  | Number | Percentage |  |
| --- | --- | --- | --- |
| Male | 1042 | 44.7% |  |
| Non-white | 249 | 10.7% |  |
| IMD 1 (least deprived) | 480 | 20.6% |  |
| IMD 2 | 509 | 21.9% |  |
| IMD 3 | 462 | 19.8% |  |
| IMD 4 | 437 | 18.8% |  |
| IMD 5 (most deprived) | 441 | 18.9% |  |
| Current Smoker | 446 | 19.1% |  |
| Past Smoker | 684 | 29.4% |  |
| Hypertension | 615 | 26.4% |  |
|  | Mean | Standard deviation | Median |
| Age (years) | 57.1 | 17.6 | 59 |
| BMI (kg/m2) | 28.5 | 5.4 | 28 |
| Systolic Blood Pressure (mmHg) | 129.9 | 17.7 | 130 |
| Total Cholesterol (mmol/l) | 5.2 | 1.1 | 5.2 |
| HDL Cholesterol (mmol/l)l | 1.5 | 0.47 | 1.5 |
| HbA1c (mmol/l) | 6.2 | 0.15 | 6.2 |

## HSE 2014 Missing data imputation

#### Ethnicity

Only a small number of individuals had missing data for ethnicity. In the QRISK2 algorithm the indicator for white included individuals for whom ethnicity is not recorded. In order to be consistent with the QRISK2 algorithm we assumed that individuals with missing ethnicity data were white.

#### Anthropometric data

Data were imputed using linear regression models to describe patterns observed within the dataset. Simple ordinary least squares (OLS) regression models were used to predict missing data. Missing data were sampled stochastically from the conditional distributions to allow variability in imputed values.

Summary data for each measure confirmed that the data were approximately normally distributed. Covariate selection was made by selecting the anthropometric measure that maximised the Adjusted R-squared statistic, and age and sex were included if the coefficients were statistically significant (P<0.1). For height and weight, waist circumference was found to improve model fit.

#### Metabolic data

Imputation models for metabolic data were developed utilising observations from other measures to help improve their accuracy.

Two imputation models were generated for each of the following metabolic measures: total cholesterol, HDL cholesterol, HbA1c, and SBP. The first imputation method included an alternative metabolic measure to improve precision. The second included only age and/or sex, to be used if the alternative measure was also missing. Simple ordinary least squares (OLS) regression models were used to predict missing data. Summary data for each measure confirmed that the data were approximately normally distributed. Covariate selection was made by selecting the metabolic measure that maximised the Adjusted R-squared statistic, and age and sex were included if the coefficients were statistically significant (P<0.1).

#### Treatment for Hypertension and Statins

A large proportion of individuals had missing data for questions relating to whether they received treatment for hypertension or high cholesterol. The majority of non-responses to these questions were coded to suggest that the question was not applicable to the individual. As a consequence it was assumed that individuals with missing treatment data were not taking these medications.

#### Anxiety/Depression

Most individuals who had missing data for anxiety and depression did so because the question was not applicable. A small sample N=69 refused to answer the question. We assumed that individuals with missing data for anxiety and depression did not have severe anxiety/depression.

#### Smoking

Individuals with missing data for smoking status were assumed to be non-smokers, without a history of smoking.

#### Rheumatoid Arthritis

Indiivduals reporting existing arthritis/rheumatism were assigned to a history rheumatoid arthritis.

**Atrial Fibrillation**

Individuals reporting “other heart conditions” in response to questions about long-standing illnesses were assumed to have a history of Atrial Fibrillation.

#### Family history of diabetes

No questions in the HSE referred to the individual having a family history of diabetes, so this data had to be imputed. It was important that data was correlated with other risk factors for diabetes, such as HbA1c and ethnicity. We analysed a cross-section of the Whitehall II dataset to generate a logistic regression to describe the probability that an individual has a history of diabetes conditional on their HbA1c and ethnic origin. The model is described in Table 3.

**Table 4: Imputation model for history of diabetes**

|  | Coefficient | Standard error |
| --- | --- | --- |
| Intercept | -3.29077 | 0.4430 |
| HbA1c | 0.28960 | 0.0840 |
| HDL Cholesterol | 0.81940 | 0.1388 |

**History of Cardiovascular disease**

Individuals with a history of cardiovascular disease were assigned to a health status of either stable angina, unstable angina, myocardial infarction, or stroke based on responses to health survey for England responses to long standing conditions. Individuals reporting stroke were assigned to stroke, heart attak/angina to unstable angina and MI at random using distributions estimated in the statins HTA (7).

# GP Attendance in the General Population

GP visit frequency was simulated in the dataset for two reasons; firstly to estimate healthcare utilisation for the general population; secondly to predict the likelihood that individuals participate in opportunistic screening for diabetes and vascular risks. It was useful to develop a model of GP attendance to be conditional on characteristics in the cost-effectiveness model that are known to be associated with GP attendance, such as age and comorbidities. A negative binomial model was used to generate count data and a skewed distribution as observed in the dataset.

|  | $\mu_{i}=exp(x_{i}\beta)$ |  |
| --- | --- | --- |

The dispersion parameter of the Negative Binomial distribution $v_{i}$ was sampled from a gamma distribution with mean 1 and variance $\alpha$ based on estimates reported in Table 5. The dose was estimated from the Poisson function.

|  | $p\left( Y=y \vert y>0,x \right)=\frac{\left( v_{i}\mu_{i} \right)^{y}e^{-\left( v_{i}\mu_{i} \right)}}{y!}$ |  |
| --- | --- | --- |

The HSE 2014 did not collect data on GP attendance frequency, therefore an alternative UK survey was sought. The South Yorkshire cohort collected data about the frequency of GP attendance in the past 3 months from a representative cross-section of individuals in South Yorkshire (8). All individuals in the cohort were included in the analysis, including those with diabetes. The characteristics of the study population are reported in Table 4.

Table 5: Characteristics of the first wave of the South Yorkshire Cohort (N=27,806)

|  | Number | Percentage |  |
| --- | --- | --- | --- |
| Male | 12,155 | 43.7 |  |
| White | 26,419 | 95.0 |  |
| Non-smoker | 23,158 | 83.3 |  |
| Employed (inc. self-employed) | 18,502 | 66.5 |  |
| Long-standing illness (any) | 16,664 | 60.0 |  |
| Diabetes | 2,000 | 7.2 |  |
| Cardiovascular disease | 2,438 | 8.8 |  |
| Hypertension | 5,653 | 20.3 |  |
|  | Mean | Standard deviation | Median |
| Age | 54.45 | 17.25 | 57.00 |
| BMI | 26.46 | 5.05 | 25.68 |
| EQ-5D (TTO) | 0.803 | 0.253 | 0.848 |
| GP attendances in past 3 months | 2.03 | 1.83 | 1.00 |
| BMI Body Mass Index; EQ-5D 5 dimensions Euroqol (health related quality of life index) | | | |

The coefficients of the Negative Binomial model described in Table 5 were used to estimate the first parameter of the Negative Binomial distribution$\mu_{i}$. Analysis of the South Yorkshire cohort (Table 5) was used to describe GP attendance conditional on age, sex, BMI, ethnicity, and health outcomes. The estimated number of GP visits was multiplied by 4 to reflect the annual number of visits per year. In the probabilistic sensitivity analysis the parameters of the South Yorkshire negative binomial model are sampled from a multivariate normal distribution, using the mean estimates described in Table 5 and covariance matrix in Table 6.

Table 6: GP attendance reported in the South Yorkshire Cohort (N= 18,437)

|  | Mean | Standard error | Uncertainty Distribution |
| --- | --- | --- | --- |
| Age | 0.0076 | 0.0005 | MULTIVARIATE NORMAL |
| Male | -0.1495 | 0.0159 | MULTIVARIATE NORMAL |
| BMI | 0.0110 | 0.0015 | MULTIVARIATE NORMAL |
| Ethnicity (Non-white) | 0.2620 | 0.0375 | MULTIVARIATE NORMAL |
| Heart Disease | 0.2533 | 0.0289 | MULTIVARIATE NORMAL |
| Depression | 0.6127 | 0.0224 | MULTIVARIATE NORMAL |
| Osteoarthritis | 0.2641 | 0.0238 | MULTIVARIATE NORMAL |
| Diabetes | 0.2702 | 0.0278 | MULTIVARIATE NORMAL |
| Stroke | 0.1659 | 0.0474 | MULTIVARIATE NORMAL |
| Cancer | 0.2672 | 0.0414 | MULTIVARIATE NORMAL |
| Intercept | -0.5014 | 0.0468 | MULTIVARIATE NORMAL |
| Alpha | 0.3423 | 0.0108 | MULTIVARIATE NORMAL |

Table 7: Variance-covariance matrix for GP attendance regression

|  | Age | Male | BMI | Ethnicity (Non-white) | Heart Disease | Depression | Osteoarthritis | Diabetes | Stroke | Cancer | Intercept | Alpha |
| --- | --- | --- | --- | --- | --- | --- | --- | --- | --- | --- | --- | --- |
| Age | 0.0000 |  |  |  |  |  |  |  |  |  |  |  |
| Male | 0.0000 | 0.0003 |  |  |  |  |  |  |  |  |  |  |
| BMI | 0.0000 | 0.0000 | 0.0000 |  |  |  |  |  |  |  |  |  |
| Ethnicity (Non-white) | 0.0000 | 0.0000 | 0.0000 | 0.0014 |  |  |  |  |  |  |  |  |
| Heart Disease | 0.0000 | 0.0000 | 0.0000 | 0.0000 | 0.0008 |  |  |  |  |  |  |  |
| Depression | 0.0000 | 0.0000 | 0.0000 | 0.0000 | 0.0000 | 0.0005 |  |  |  |  |  |  |
| Osteoarthritis | 0.0000 | 0.0000 | 0.0000 | 0.0000 | 0.0000 | 0.0000 | 0.0006 |  |  |  |  |  |
| Diabetes | 0.0000 | 0.0000 | 0.0000 | 0.0000 | -0.0001 | 0.0000 | 0.0000 | 0.0008 |  |  |  |  |
| Stroke | 0.0000 | 0.0000 | 0.0000 | 0.0000 | -0.0002 | -0.0001 | 0.0000 | -0.0001 | 0.0022 |  |  |  |
| Cancer | 0.0000 | 0.0000 | 0.0000 | 0.0000 | 0.0000 | 0.0000 | 0.0000 | 0.0000 | -0.0001 | 0.0017 |  |  |
| Intercept | 0.0000 | 0.0000 | -0.0001 | -0.0002 | 0.0002 | 0.0000 | 0.0002 | 0.0003 | 0.0000 | 0.0001 | 0.0022 |  |
| Alpha | 0.0000 | 0.0000 | 0.0000 | 0.0000 | 0.0000 | 0.0000 | 0.0000 | 0.0000 | 0.0000 | 0.0000 | 0.0000 | 0.0010 |

# Longitudinal Trajectories of Metabolic Risk Factors

Two separate sets of statistical analyses of longitudinal cohort studies were used to describe metabolic trajectories for individuals in the model. An analysis of the Whitehall II cohort study (6) was developed to describe correlated longitudinal changes in metabolic risk factors for individuals aged 60 years and younger. An analysis of the Englished Longitudinal Study of Ageing was used to describe trajectories for individuals aged 61 and over. The transition point of 61 years was found to be the age at which there were more data observations for participants in ELSA compared with Whitehall. A summary for each set of metabolic trajectory models are provided below.

## Whitehall II Data Analysis

Changes in BMI, latent blood glucose, total cholesterol, HDL cholesterol and SBP were estimated from statistical analysis of the Whitehall II cohort. The growth factors for all 5 risk factors were estimated using parallel latent growth modelling. This enabled the growth factors for BMI to be implemented as covariates for the growth processes of glycaemia, systolic blood pressure, and total cholesterol^[[1]](#footnote-1)^. The structural assumptions of the analysis are described in more detail below.

In the Whitehall II data analysis it was assumed that individuals have an underlying level of glycaemia, which cannot be observed but can be measured by HbA1c, FPG, and 2-hour glucose. This underlying propensity for diabetes is referred to as latent glycaemia. The statistical model estimated the unobservable latent glycaemia, and from this identified associations with test results for HbA1c, FPG, and 2-hour glucose. The longitudinal changes in BMI, glycaemia, SBP, total cholesterol and HDL cholesterol could then be estimated through statistical analysis.

These growth factors are conditional on several individual characteristics including age, sex, ethnicity, smoking, family history of CVD, and family history of type 2 diabetes. We related the effect of changes in BMI to changes in glycaemia, SBP and total cholesterol. However, if an intervention is known to be effective in reducing BMI and the other metabolic risk factors, the Whitehall II model is adjusted to temporarily remove the indirect effect of the intervention through BMI. This ensures that the effectiveness of the intervention is not over-estimated. Unobservable heterogeneity between individual growth factors not explained by patient characteristics was incorporated into the growth models as random error terms. Correlation between the random error terms for glycaemia, total cholesterol, HDL cholesterol and systolic blood pressure was estimated from the Whitehall II cohort. This means that in the simulation, an individual with a higher growth rate for glycaemia was more likely to have a higher growth rate of total cholesterol and SBP.

An advantage of this parallel growth analysis is that it was able to estimate the effect of growth in BMI on the other metabolic risk factors. The statistical analysis also described the correlation between changes in glycaemia, SBP, total cholesterol and HDL cholesterol. As a consequence, the growth factor random error terms were not assumed to be independent and were sampled from a multivariate normal distribution$\boldsymbol{\upsilon}\sim N(0,\Omega)$. Estimates for the covariance matrix are derived from the covariance estimates reported in the statistical analysis.

The baseline observations for BMI, HbA1c, SBP, cholesterol and HDL cholesterol were extracted from the Health Survey for England 2014 in order to simulate a representative sample of the UK population. The predicted intercept for these metabolic risk factors was estimated using the Whitehall II analysis to give population estimates of the individuals’ starting values, conditional on their characteristics. The difference between the simulated and observed baseline risk factors was taken to estimate the individuals’ random deviation from the population expectation. The individual random error in the slope trajectory was sampled from a conditional multivariate normal distribution to allow correlation between the intercept and slope random errors.

Figure 2: Path analysis of final statistical analysis of the Whitehall II cohort

## ELSA Data Analysis

Changes in BMI, HbA1c, systolic blood pressure, total cholesterol and HDL cholesterol were estimated from a statistical analysis of the ELSA cohort. The changes with age were estimated using independently estimate random coefficient growth models in Stata 13. The growth trajectory models for the metabolic risk factor were estimated under the statistical framework of growth curve modelling (GCM) (9). GCM is an approach to using longitudinal data to estimate shape and rate of change over time. GCM was chosen because it can allow modelling of variability in participants fixed and slope parameters. The growth factors for the metabolic risk factors were assumed to vary between individuals to allow unobservable random effects to describe the heterogeneity in intercept and slope parameters. Assessment of the data indicated that there was significant variance in the intercept (risk factor starting value) and slope (change in risk factor over time) for all metabolic risks. The growth factor models without covariates were specified as.

$$Y_{ij}=\left( \beta_{1}+\zeta_{1j} \right)+\left( \beta_{2}+\zeta_{2j} \right)t_{ij}+\epsilon_{ij}$$

Where Y describes the observed metabolic risk factor for individual i at time j, $\beta_{1}$ is the population mean intercept, and $\beta_{2}$ population mean slope. The random factors $\zeta_{1j}$ and $\zeta_{2j}$ describe the random variability across individuals in the intercept and slopes respectively. The statistical models were weighted for selection bias using nurse visit weights for BMI and systolic blood pressure and blood sample weights for Total Cholesterol, HDL Cholesterol and HbA1c supplied by the ELSA dataset to improve the representativeness of the analysis for an English population.

The model assumed that BMI growth was quadratic with age, due to trends observed in the data and in other cohorts (10). HbA1c, systolic blood pressure, total cholesterol and HDL cholesterol were assumed to be linear with time. All model intercepts and slopes were adjusted for sex, smoking status, deprivation and ethnicity. Anti-hypertensive treatment was included as an additional covariate in the systolic blood pressure model. Unfortunately data on statin were not available for any wave except wave 6, therefore we did not include this as a covariate in the analysis. Covariates were included in the final model if the variable was statistical significant with a p-value less than 0.1.

A full list of model parameters estimated from the statistical analysis of the Whitehall II data and ELSA data are presented in Supplementary file 2.

## BMI Trajectory

At baseline, BMI estimates from the HSE determine an individual’s BMI. If the individual is aged 60 years or less annual changes in BMI are calculated from the Whitehall II study based on population average changes for the individual and a sampled random coefficient factor. From ages 61 and over the ELSA BMI statistical model is used estimate their older age trajectory in BMI. New random coefficient growth factors are estimated based on the covariance structure of the ELSA random intercept and slope. As a consequence, current BMI status is informative in determining the future trajectory of BMI.

## Glycaemic Trajectory in Non-Diabetics/undiagnosed Diabetes

At baseline, HbA1c estimates for HbA1c are used to determine an individual’s HbA1c and glycaemic status. For individuals aged 60 years or less the Whitehall II study is used to estimate annual changes in HbA1c, and through latent glycaemia FPG, and 2-hr glucose observations. In the Whitehall II analysis we assume that changes in latent glycaemia have a quadratic relationship with time. The Whitehall II models allow random coefficient factors for growth in glycaemia for an individual and measurement error in test results according to estimated parameters from the Whitehall II analysis. For individuals aged 61 and over the ELSA statistical model is used to estimate an individuals linear changes in HbA1c evry year. Random coefficient growth factors are re-estimated using the bivariate covariance structure from the ELSA HbA1c growth model. It is not possible to estimate FPG and 2-hr glucose using the ELSA statistical models.

## HbA1c trajectory in type 2 diagnosed diabetics

Following a diagnosis of diabetes in the simulation all individuals experience an initial fall in HbA1c due to changes in diet and lifestyle as observed in the UKPDS trial (11). We have estimated the expected change in HbA1c conditional on HbA1c at diagnosis by fitting a simple linear regression to three aggregate outcomes reported in the study. These showed that the change in HbA1c increases for higher HbA1c scores at diagnosis. The regression parameters to estimate change in HbA1c are reported in Table 7.

Table 8: Estimated change in HbA1c following diabetes diagnosis

|  | Mean | Standard error |
| --- | --- | --- |
| Change in HbA1c Intercept | -2.9465 | 0.0444513 |
| HbA1c at baseline | 0.5184 | 0.4521958 |

After this initial reduction in HbA1c the longitudinal trajectory of HbA1c is estimated using the UKPDS outcomes model (11) rather than the Whitehall II statistical analysis. The UKPDS dataset is made up of a newly diagnosed diabetic population. As part of the UKPDS Outcomes model, longitudinal trial data were analysed using a random effects model. The coefficients of the model are reported in Table 8.

**Table 9: Coefficient estimates for HbA1c estimated from UKPDS data**

|  | Mean Coefficient | Coefficient standard error |
| --- | --- | --- |
| Intercept | -0.024 | 0.017 |
| Log transformation of year since diagnosis | 0.144 | 0.009 |
| Binary variable for year after diagnosis | -0.333 | 0.05 |
| HbA1c score in last period | 0.759 | 0.004 |
| HbA1c score at diagnosis | 0.085 | 0.004 |

The model can be used to predict HbA1c over time from the point of diagnosis. The model suggests that HbA1c increases with time. A graph illustrating change in HbA1c over time from two different HbA1c levels at diagnosis is illustrated in Figure 3.

Figure 3: Trajectory of HbA1c estimated from UKPDS longitudinal model

##

## Total Cholesterol and HDL Cholesterol Trajectories in Individuals not receiving Statins

At baseline, an individual’s total and HDL cholesterol is determined from the HSE 2014 data. In the simulation, individuals aged 60 years and younger have annual changes in total and HDL cholesterol according to the estimates from the statistical analysis of the Whitehall II cohort. The slope of total and HDL cholesterol are assumed to be linear with time. These growth factors are estimated in the model to be conditional on cholesterol at baseline, age at baseline, sex, and an error parameter to reflect unobservable variability in growth trajectories between individuals. As with latent glycaemia, changes in total cholesterol are also influenced by the trajectory of BMI. For individuals aged 61 and over the ELSA statistical models for Total and HDL cholesterol are used to estimate annual change in cholesterol. As individuals transition between the trajectory models the random coefficient factors are updated allowing current observations to inform the trajectories in Total and HDL cholesterol.

## Total Cholesterol and HDL Cholesterol Trajectories in Individuals receiving Statins

During the simulation process, individuals are prescribed statins to reduce their risk of cardiovascular disease. It is assumed within the model that the statins are effective in reducing an individual’s total cholesterol, and an average effect is applied to all patients receiving statins. A recent HTA reviewed the literature on the effectiveness and cost-effectiveness of statins in individuals with acute coronary syndrome (12). This report estimated the change in LDL cholesterol for four statin treatments and doses compared with placebo from a Bayesian meta-analysis. The analysis estimated a reduction in LDL cholesterol of -1.45 for simvastatin. This estimate was used to describe the effect of statins in reducing total cholesterol. It was assumed that the effect was instantaneous upon receiving statins and maintained as long as the individual receives statins. It was also assumed that individuals receiving statins no longer experienced annual changes in total cholesterol. HDL cholesterol was assumed constant over time if patients receive statins.

Non-adherence to statin treatment is a common problem. Two recent HTAs reviewed the literature on continuation and compliance with statin treatment. They both concluded that there was a lack of adequate reporting, but that the proportion of patients fully compliant with treatment appears to decrease with time, particularly in the first 12 months after initiating treatment, and can fall below 60% after five years (7;12). Although a certain amount of non-compliance is included within trial data, clinical trials are not considered to be representative of continuation and compliance in general practice. A yearly reduction in statin compliance used in the HTA analysis is reported in Table 9. It is based on the published estimate of compliance for the first five years of statin treatment for primary prevention in general clinical practice (12). Compliance declines to a minimum of 65% after five years of treatment. It is assumed that there is no further drop after five years.

Table 10: Proportion of patients assumed to be compliant with statin treatment, derived from Table 62 in (12)

| Year after statin initiation | 1 | 2 | 3 | 4 | 5 |
| --- | --- | --- | --- | --- | --- |
| Proportion compliant | 0.8 | 0.7 | 0.68 | 0.65 | 0.65 |

In the simulation, we assume in the base case that only 65% of individuals initiate statins when they are deemed eligible. However those that initiate statins remain on statins for their lifetime. Those who refuse statins may be prescribed them again at a later date.

## SBP Trajectories in Individuals Not receiving Anti-hypertensive treatment

At baseline an individual’s SBP is determined from the HSE 2014 data. In the simulation, individuals’ aged 60 and younger experience SBP changes every year according to the estimates from the statistical analysis of the Whitehall II cohort. The annual change in SBP is assumed to be linear with time. The growth factors are estimated in the model to be conditional on SBP at baseline, age at baseline, sex, ethnicity, family history of cardiovascular disease, smoking and an error parameter to reflect unobservable variability in growth trajectories between individuals. From ages 61 onwards the ELSA statistical model for systolic blood pressure is used to estimate annual changes. The random coefficient factors are updated using the bivariate covariance matrix for intercept and slope factors.

## SBP Trajectories in Individuals receiving anti-hypertensive treatment

During the simulation process, if individuals are identified as having SBP higher than 160mm Hg, or SBP higher than 140mm Hg with comorbid diabetes, cardiovascular disease, or 10 year risk of cardiovascular disease greater than 20%, they will be prescribed anti-hypertensive treatment in line with the National Institute for Health and Care Excellence (NICE) guidelines (13). The change in SBP following initiation of calcium channel blockers was estimated in a meta-analysis of anti-hypertensive treatments (14). This study identified an average change in SBP of -8.4 for monotherapy with calcium channel blockers. In the simulation model it is assumed that this reduction in SBP is maintained for as long as the individual receives anti-hypertensive treatment. Once an individual is receiving anti-hypertensive treatment it is assumed that their SBP is stable and does not change over time, which implicitly assumes that patients continue to be well managed for their hypertension. For simplicity we do not explicitly simulate treatment switching. The assumed zero flat trajectory in systolic blood pressure whilst receiving anti-hypertensives is supported by the analysis of the ELSA dataset in which self-reported use of anti-hypertensives was included as a covariate for age-related change in systolic blood pressure. The analysis found that most of the observed changes in systolic blood pressure were removed if individuals were taking anti-hypertensives.

## Metabolic Risk factor screening

We assume that individuals eligible for anti-hypertensive treatment or statins will be identified through opportunistic screening if they meet certain criteria and attend the GP for at least one visit in the simulation period.

1. Individuals with a history of cardiovascular disease;
2. Individuals with a major microvascular event (foot ulcer, blindness, renal failure or amputation);
3. Individuals with diagnosed diabetes;
4. Individuals identified with Impaired Glucose Regulation;
5. Individuals with systolic blood pressure greater than 160mmHg.

Individuals may also be detected for diabetes through opportunistic screening if the following criteria are met.

1. Individuals with a history of cardiovascular disease;
2. Individuals with a major microvascular event (foot ulcer, blindness, renal failure or amputation);
3. Individuals identified with impaired glucose regulation;
4. At baseline individuals are assigned an HbA1c threshold above which diabetes is detected opportunistically, individuals with an HbA1c above their individual threshold will attend the GP to be diagnosed with diabetes. The threshold is sampled from the distribution of HbA1c tests in a cohort of recently diagnosed patients in clinical practice (15).

The base case has been designed to represent a health system with moderate levels of screening for hypertension, diabetes, and dyslipidaemia. Alternative assumptions for more or less intensive opportunistic screening can be assumed.

## Diagnosis and Treatment Initiation

It is assumed that there are three, non-mutually exclusive outcomes from the vascular checks or opportunistic screening. Firstly, that the patient receives statins to reduce cardiovascular risk. Secondly, that the patient has high blood pressure and should be treated with anti-hypertensive medication. Thirdly, the model evaluates whether the blood glucose test indicates a type 2 diabetes diagnosis. The following threshold estimates were used to determine these outcomes.

1. Statins are initiated if the individual has greater than or equal to 20% 10 year CVD risk estimated from the QRISK2 2012 algorithm (16).
2. Anti-hypertensive treatment is initiated if systolic blood pressure is greater than 160. If the individual has a history of CVD, diabetes or a CVD risk >20%, the threshold for systolic blood pressure is 140 (13).
3. Type 2 diabetes is diagnosed if the individual has two HbA1c tests greater than 6.5. In the base case it is assumed that FPG and 2-hr glucose are not used for diabetes diagnosis. However, future adaptations of the model could use these tests for diagnosis.

# Comorbid Outcomes and Mortality

In every model cycle individuals within the model are evaluated to determine whether they have a clinical event, including mortality, within the cycle period. In each case the simulation estimates the probability that an individual has the event and uses a random number draw to determine whether the event occurred.

## Cardiovascular Disease

### First Cardiovascular event

Several statistical models for cardiovascular events were identified in a review of economic evaluations for diabetes prevention (17). The UKPDS outcomes model (11;18), Framingham risk equation (19) and QRISK2 (20) have all been used in previous models to estimate cardiovascular events. The Framingham risk equation was not adopted because, unlike the QRISK2 model, it is not estimated from a UK population. The UKPDS outcomes model would be ideally suited to estimate the risk of cardiovascular disease in a population diagnosed with type 2 diabetes. Whilst this is an important outcome of the cost-effectiveness model, there was concern that it would not be representative of individuals with normal glucose tolerance or impaired glucose regulation. Recent analyses show that the UKPDS over-predicts cardiovascular outcomes in newly diagnosed diabetes patients (21). It was important that reductions in cardiovascular disease risk in these populations were represented to capture the population-wide benefits of public health interventions. The QRISK2 model was selected for use in the cost-effectiveness model because it is a validated model of cardiovascular risk in a up to date UK population and could be used to generate probabilities for diabetic and non-diabetic populations. We considered using the UKPDS outcomes model specifically to estimate cardiovascular risk in patients with type 2 diabetes. However, it would not be possible to control for shifts in absolute risk generated by the different risk scores due to different baselines and covariates. This would lead to some individuals experiencing counterintuitive and favourable shifts in risk after onset of type 2 diabetes. Therefore, we decided to use diabetes as a covariate adjustment to the QRISK2 model to ensure that the change in individual status was consistent across individuals.

The probability of the first cardiovascular event is estimated from the QRISK2 predicted model of cardiovascular disease (20). The QRISK2 is a validated risk prediction algorithm to identify individuals at high risk of cardiovascular disease. The algorithm was developed from UK data and incorporates social deprivation and ethnicity. We accessed the 2012 version from the online QRISK website (22). The QRISK2 equation estimates the probability of a cardiovascular event in the next year conditional on ethnicity, smoking status, age, BMI, ratio of total/HDL cholesterol, Townsend score, atrial fibrillation, rheumatoid arthritis, renal disease, hypertension, diabetes, and family history of cardiovascular disease. Data on all these variables was available from the HSE 2014. Table 10 reports the coefficient estimates for the QRISK2 algorithm. The standard errors were not reported within the open source code. Where possible, standard errors were imputed from a previous publication of the risk equation (23). Coefficients that were not reported in this publication were assumed to have standard errors of 20%.

Table 11: Coefficients from the 2012 QRISK2 risk equation and estimate standard errors

|  | Estimated coefficients adjusting for individual characteristics | | | | | | | | |
| --- | --- | --- | --- | --- | --- | --- | --- | --- | --- |
|  | Women | | Men | |  | Women | | Men | |
| Covariates | Mean | Standard error | Mean | Mean | Interaction terms | Mean | Standard error | Mean | Standard error |
| White | 0.0000 | 0.0000 | 0.0000 | 0.0000 | Age1*former smoker | 0.1774 | 0.035 | -3.881 | 0.776 |
| Indian | 0.2163 | 0.0537 | 0.3163 | 0.0425 | Age1*light smoker | -0.3277 | 0.066 | -16.703 | 3.341 |
| Pakistani | 0.6905 | 0.0698 | 0.6092 | 0.0547 | Age1*moderate smoker | -1.1533 | 0.231 | -15.374 | 3.075 |
| Bangladeshi | 0.3423 | 0.1073 | 0.5958 | 0.0727 | Age1*Heavy smoker | -1.5397 | 0.308 | -17.645 | 3.529 |
| Other Asian | 0.0731 | 0.1071 | 0.1142 | 0.0845 | Age1*AF | -4.6084 | 0.922 | -7.028 | 1.406 |
| Caribbean | -0.0989 | 0.0619 | -0.3489 | 0.0641 | Age1*renal disease | -2.6401 | 0.528 | -17.015 | 3.403 |
| Black African | -0.2352 | 0.1275 | -0.3604 | 0.1094 | Age1*hypertension | -2.2480 | 0.450 | 33.963 | 6.793 |
| Chinese | -0.2956 | 0.1721 | -0.2666 | 0.1538 | Age1*Diabetes | -1.8452 | 0.369 | 12.789 | 2.558 |
| Other | -0.1010 | 0.0793 | -0.1208 | 0.0734 | Age1*BMI | -3.0851 | 0.617 | 3.268 | 0.654 |
| Non-smoker | 0.0000 | 0.0000 | 0.0000 | 0.0000 | Age1*family history CVD | -0.2481 | 0.050 | -17.922 | 3.584 |
| Former smoker | 0.2033 | 0.0152 | 0.2684 | 0.0108 | Age1*SBP | -0.0132 | 0.003 | -0.151 | 0.030 |
| Light smoker | 0.4820 | 0.0220 | 0.5005 | 0.0166 | Age1*Townsend | -0.0369 | 0.007 | -2.550 | 0.510 |
| Moderate smoker | 0.6126 | 0.0178 | 0.6375 | 0.0148 | Age2*former smoker | -0.0051 | 0.001 | 7.971 | 1.594 |
| Heavy smoker | 0.7481 | 0.0194 | 0.7424 | 0.0143 | Age2*light smoker | -0.0005 | 0.000 | 23.686 | 4.737 |
| Age 1* | 5.0327 |  | 47.3164 |  | Age2*moderate smoker | 0.0105 | 0.002 | 23.137 | 4.627 |
| Age 2* | -0.0108 |  | -101.2362 |  | Age2*Heavy smoker | 0.0155 | 0.003 | 26.867 | 5.373 |
| BMI* | -0.4724 | 0.0423 | 0.5425 | 0.0299 | Age2*AF | 0.0507 | 0.010 | 14.452 | 2.890 |
| Ratio Total / HDL chol | 0.1326 | 0.0044 | 0.1443 | 0.0022 | Age2*renal disease | 0.0343 | 0.007 | 28.270 | 5.654 |
| SBP | 0.0106 | 0.0045 | 0.0081 | 0.0046 | Age2*hypertension | 0.0258 | 0.005 | -18.817 | 3.763 |
| Townsend | 0.0597 | 0.0068 | 0.0365 | 0.0048 | Age2*Diabetes | 0.0180 | 0.004 | 0.963 | 0.193 |
| AF | 1.3261 | 0.0310 | 0.7547 | 0.1018 | Age2*BMI | 0.0345 | 0.007 | 10.551 | 2.110 |
| Rheumatoid arthritis | 0.3626 | 0.0319 | 0.3089 | 0.0445 | Age2*family history CVD | -0.0062 | 0.001 | 26.605 | 5.321 |
| Renal disease | 0.7636 | 0.0639 | 0.7441 | 0.0702 | Age2*SBP | 0.0000 | 0.000 | 0.291 | 0.058 |
| Hypertension | 0.5421 | 0.0115 | 0.4978 | 0.0112 | Age2*Townsend | -0.0011 | 0.000 | 3.007 | 0.601 |
| Diabetes | 0.8940 | 0.0199 | 0.7776 | 0.0175 |  |  |  |  |  |
| Family history of CVD | 0.5997 | 0.0122 | 0.6965 | 0.0111 |  |  |  |  |  |
| AF Atrial Fibrillation CVD Cardiovascular disease SBP systolic blood pressure * covariates transformed with fractional polynomials | | | | | | | | | |

The QRISK2 risk equation can be used to calculate the probability of a cardiovascular event including: coronary heart disease (angina or myocardial infarction), stroke, or transient ischaemic attacks, fatality due to cardiovascular disease. The equation estimates the probability of a cardiovascular event in the next period conditional on the coefficients listed in Table 10. The equation for the probability of an event in the next period is calculated as

$$p\left( Y=1 \right)=1-{S(1)}^{\theta}$$

$$\theta=\sum\beta X$$

The probability of an event is calculated from the survival function at 1 year raised to the power of $\theta$, where $\theta$ is the sum product of the coefficients reported in Table 10 multiplied by the individual’s characteristics. Underlying survival curves for men and women were extracted from the QRISK2 open source file. Mean estimates for the continuous variables were also reported in the open source files.

We modified the QRISK2 assumptions regarding the relationship between IGR, diabetes and cardiovascular disease. Firstly, we assumed that individuals with HbA1c>6.5 have an increased risk of cardiovascular disease even if they have not received a formal diagnosis. Secondly, risk of cardiovascular disease was assumed to increase with HbA1c for test results greater than 6.5 to reflect observations from the UKPDS that HbA1c increases the risk of MI and Stroke (18) . Thirdly, prior to type 2 diabetes (HbA1c>6.5) HbA1c is linearly associated with cardiovascular disease. A study from the EPIC Cohort has found that a unit increase in HbA1c increases the risk of coronary heart disease by a hazard ratio of 1.25, after adjustment for other risk factors (24). We apply this risk ratio to linearly increase risk above the mean HBA1c observed in the HSE 2011 cohort. A linear risk reduction was applied at HbA1c levels below the HSE mean.

The QRISK2 algorithm identifies which individuals experience a cardiovascular event but does not specify the nature of the event. The nature of the cardiovascular event was determined independently. A targeted search of recent Health Technology appraisals of cardiovascular disease was performed to identify a model for the progression of cardiovascular disease following a first event. A Health Technology Assessment (HTA) assessing statins gives age and sex specific distributions of CVD, which were used to assign all QRISK2 events (7). Table 11 reports the probability of cardiovascular outcomes by age and gender. Stakeholders suggested that there may be different relationships between the risk factors and the different types of CVD (e.g. hypertension is more of a risk factor for stroke). However, we decided not to incorporate these differential factors in evaluating the risk of cardiovascular event types into the model due to a lack of evidence.

Table 12: The probability distribution of cardiovascular events by age and gender

|  | Age | Stable angina | Unstable angina | MI rate | Fatal CHD | TIA | Stroke | Fatal CVD |
| --- | --- | --- | --- | --- | --- | --- | --- | --- |
| Men | 45-54 | 0.307 | 0.107 | 0.295 | 0.071 | 0.060 | 0.129 | 0.030 |
|  | 55-64 | 0.328 | 0.071 | 0.172 | 0.086 | 0.089 | 0.206 | 0.048 |
|  | 65-74 | 0.214 | 0.083 | 0.173 | 0.097 | 0.100 | 0.270 | 0.063 |
|  | 75-84 | 0.191 | 0.081 | 0.161 | 0.063 | 0.080 | 0.343 | 0.080 |
|  | 85+ | 0.214 | 0.096 | 0.186 | 0.055 | 0.016 | 0.351 | 0.082 |
| Women | 45-54 | 0.325 | 0.117 | 0.080 | 0.037 | 0.160 | 0.229 | 0.054 |
|  | 55-64 | 0.346 | 0.073 | 0.092 | 0.039 | 0.095 | 0.288 | 0.067 |
|  | 65-74 | 0.202 | 0.052 | 0.121 | 0.081 | 0.073 | 0.382 | 0.090 |
|  | 75-84 | 0.149 | 0.034 | 0.102 | 0.043 | 0.098 | 0.464 | 0.109 |
|  | 85+ | 0.136 | 0.029 | 0.100 | 0.030 | 0.087 | 0.501 | 0.117 |

### Subsequent Cardiovascular events

After an individual has experienced a cardiovascular event, it is not possible to predict the transition to subsequent cardiovascular events using QRISK2. As with assigning first CVD events, the probability of subsequent events was estimated from the HTA evaluating statins (7). This study reported the probability of future events conditional on the nature of the previous event. Table 12 reports an example of the probabilities within a year of transitioning from stable angina, unstable angina, myocardial infarction (MI), transient ischemic attack (TIA) or stroke for individuals by age group.

Table 13: Probability of cardiovascular event conditional on age and status of previous event (column1)

|  | Stable angina | Unstable angina 1 | Unstable angina 2 | MI 1 | MI 2 | TIA | Stroke 1 | Stroke 2 | CHD death | CVD death |
| --- | --- | --- | --- | --- | --- | --- | --- | --- | --- | --- |
| Age 45 |  |  |  |  |  |  |  |  |  |  |
| Stable angina | 0.9946 | 0.0013 | 0 | 0.0032 | 0 | 0 | 0 | 0 | 0.0009 | 0 |
| Unstable angina (1^st^ yr) | 0 | 0 | 0.9127 | 0.0495 | 0 | 0 | 0 | 0 | 0.0362 | 0.0016 |
| Unstable angina (subsequent) | 0 | 0 | 0.9729 | 0.0186 | 0 | 0 | 0 | 0 | 0.0081 | 0.0004 |
| MI (1^st^ yr) | 0 | 0 | 0 | 0.128 | 0.8531 | 0 | 0.0015 | 0 | 0.0167 | 0.0007 |
| MI (subsequent) | 0 | 0 | 0 | 0.0162 | 0.978 | 0 | 0.0004 | 0 | 0.0052 | 0.0002 |
| TIA | 0 | 0 | 0 | 0.0016 | 0 | 0.9912 | 0.0035 | 0 | 0.0024 | 0.0013 |
| Stroke (1^st^ yr) | 0 | 0 | 0 | 0.0016 | 0 | 0 | 0.0431 | 0.9461 | 0.0046 | 0.0046 |
| Stroke (subsequent) | 0 | 0 | 0 | 0.0016 | 0 | 0 | 0.0144 | 0.9798 | 0.0021 | 0.0021 |
| Age 55 |  |  |  |  |  |  |  |  |  |  |
| Stable angina | 0.9874 | 0.0029 | 0 | 0.0062 | 0 | 0 | 0 | 0 | 0.0035 | 0 |
| Unstable angina (1^st^ yr) | 0 | 0 | 0.8859 | 0.0497 | 0 | 0 | 0 | 0 | 0.0617 | 0.0027 |
| Unstable angina (subsequent) | 0 | 0 | 0.9548 | 0.0348 | 0 | 0 | 0 | 0 | 0.01 | 0.0004 |
| MI (1^st^ yr) | 0 | 0 | 0 | 0.1152 | 0.8483 | 0 | 0.0032 | 0 | 0.0319 | 0.0014 |
| MI (subsequent) | 0 | 0 | 0 | 0.0179 | 0.9716 | 0 | 0.001 | 0 | 0.0091 | 0.0004 |
| TIA | 0 | 0 | 0 | 0.0031 | 0 | 0.9626 | 0.0181 | 0 | 0.0092 | 0.007 |
| Stroke (1^st^ yr) | 0 | 0 | 0 | 0.0031 | 0 | 0 | 0.0459 | 0.9288 | 0.0111 | 0.0111 |
| Stroke (subsequent) | 0 | 0 | 0 | 0.0031 | 0 | 0 | 0.0186 | 0.9685 | 0.0049 | 0.0049 |
| Age 65 |  |  |  |  |  |  |  |  |  |  |
| Stable angina | 0.976 | 0.006 | 0 | 0.011 | 0 | 0 | 0 | 0 | 0.007 | 0 |
| Unstable angina (1^st^ yr) | 0 | 0 | 0.8435 | 0.0488 | 0 | 0 | 0 | 0 | 0.1031 | 0.0046 |
| Unstable angina (subsequent) | 0 | 0 | 0.9244 | 0.0632 | 0 | 0 | 0 | 0 | 0.0119 | 0.0005 |
| MI (1^st^ yr) | 0 | 0 | 0 | 0.1019 | 0.8287 | 0 | 0.0068 | 0 | 0.0599 | 0.0027 |
| MI (subsequent) | 0 | 0 | 0 | 0.0185 | 0.9634 | 0 | 0.0022 | 0 | 0.0152 | 0.0007 |
| TIA | 0 | 0 | 0 | 0.0055 | 0 | 0.9174 | 0.0423 | 0 | 0.0185 | 0.0163 |
| Stroke (1^st^ yr) | 0 | 0 | 0 | 0.0055 | 0 | 0 | 0.0481 | 0.8944 | 0.026 | 0.026 |
| Stroke (subsequent) | 0 | 0 | 0 | 0.0055 | 0 | 0 | 0.0223 | 0.9514 | 0.0104 | 0.0104 |
| Age 75 |  |  |  |  |  |  |  |  |  |  |
| Stable angina | 0.9681 | 0.0091 | 0 | 0.0158 | 0 | 0 | 0 | 0 | 0.007 | 0 |
| Unstable angina (1^st^ yr) | 0 | 0 | 0.7789 | 0.0466 | 0 | 0 | 0 | 0 | 0.1671 | 0.0074 |
| Unstable angina (subsequent) | 0 | 0 | 0.8733 | 0.1122 | 0 | 0 | 0 | 0 | 0.0139 | 0.0006 |
| MI (1^st^ yr) | 0 | 0 | 0 | 0.0874 | 0.7849 | 0 | 0.0141 | 0 | 0.1088 | 0.0048 |
| MI (subsequent) | 0 | 0 | 0 | 0.0178 | 0.953 | 0 | 0.0047 | 0 | 0.0235 | 0.001 |
| TIA | 0 | 0 | 0 | 0.008 | 0 | 0.8588 | 0.0828 | 0 | 0.0185 | 0.0319 |
| Stroke (1^st^ yr) | 0 | 0 | 0 | 0.008 | 0 | 0 | 0.0446 | 0.8302 | 0.0586 | 0.0586 |
| Stroke (subsequent) | 0 | 0 | 0 | 0.008 | 0 | 0 | 0.0246 | 0.9262 | 0.0206 | 0.0206 |
| Age 85 |  |  |  |  |  |  |  |  |  |  |
| Stable angina | 0.9601 | 0.0122 | 0 | 0.0207 | 0 | 0 | 0 | 0 | 0.007 | 0 |
| Unstable angina (1^st^ yr) | 0 | 0 | 0.6873 | 0.0425 | 0 | 0 | 0 | 0 | 0.2587 | 0.0115 |
| Unstable angina (subsequent) | 0 | 0 | 0.7878 | 0.1955 | 0 | 0 | 0 | 0 | 0.016 | 0.0007 |
| MI (1^st^ yr) | 0 | 0 | 0 | 0.0711 | 0.7053 | 0 | 0.0278 | 0 | 0.1875 | 0.0083 |
| MI (subsequent) | 0 | 0 | 0 | 0.016 | 0.9394 | 0 | 0.0091 | 0 | 0.034 | 0.0015 |
| TIA | 0 | 0 | 0 | 0.0104 | 0 | 0.838 | 0.0961 | 0 | 0.0185 | 0.037 |
| Stroke (1^st^ yr) | 0 | 0 | 0 | 0.0104 | 0 | 0 | 0.0446 | 0.702 | 0.1215 | 0.1215 |
| Stroke (subsequent) | 0 | 0 | 0 | 0.0104 | 0 | 0 | 0.0252 | 0.8894 | 0.0375 | 0.0375 |

### Congestive Heart Failure

The review of previous economic evaluations of diabetes prevention cost-effectiveness studies found that only a small number of models had included congestive heart failure as a separate outcome. Discussion with the stakeholder group identified that the UKPDS Outcomes model would be an appropriate risk model for congestive heart failure in type 2 diabetes patients. However, it was suggested that this would not be an appropriate risk equation for individuals with normal glucose tolerance or impaired glucose tolerance. The Framingham risk equation was suggested as an alternative. As described above, switching from the framgingam risk score to the UKPDS was not possible due to differences in covariate selection. The main limitations of this equation is that it is quite old, based on a non-UK population, and include diabetes as a discrete health state rather than on a continuous scale. However, a citation search of this article did not identify a more recent or UK based alternative.

Congestive heart failure was included as a separate cardiovascular event because it was not included as an outcome of the QRISK2. The Framingham Heart Study has reported logistic regressions to estimate the 4 year probability of congestive heart failure for men and women (25). The equations included age, diabetes diagnosis, BMI and systolic blood pressure to adjust risk based on individual characteristics. We used this risk equation to estimate the probability of congestive heart failure in the SPHR diabetes prevention model. Table 13 describes the covariates for the logit models to estimate the probability of congestive heart failure in men and women.

Table 14: Logistic regression coefficients to estimate the 4-year probability of congestive heart failure from the Framingham study

| Variables | Units | Regression  Coefficient | OR (95% CI) | P |
| --- | --- | --- | --- | --- |
| Men | | | | |
| Intercept |  | -9.2087 |  |  |
| Age | 10 y | 0.0412 | 1.51 (1.31-1.74) | <.001 |
| Left ventricular hypertrophy | Yes/no | 0.9026 | 2.47 (1.31-3.77) | <.001 |
| Heart rate | 10 bpm | 0.0166 | 1.18 (1.08-1.29) | <.001 |
| Systolic blood pressure | 20 mm Hg | 0.00804 | 1.17 (1.04-1.32) | 0.007 |
| Congenital heart disease | Yes/no | 1.6079 | 4.99 (3.80-6.55) | <.001 |
| Valve disease | Yes/no | 0.9714 | 2.64 (1.89-3.69) | <.001 |
| Diabetes | Yes/no | 0.2244 | 1.25 (0.89-1.76) | 0.2 |
| Women | | | | |
| Intercept |  | -10.7988 |  |  |
| Age | 10 y | 0.0503 | 1.65 (1.42-1.93) | <.001 |
| left ventricular hypertrophy | Yes/no | 1.3402 | 3.82 (2.50-5.83) | <.001 |
| Heart rate | 100 cL | 0.0105 | 1.11 (1.01-1.23) | 0.03 |
| Systolic blood pressure | 10 bpm | 0.00337 | 1.07 (0.96-1.20) | 0.24 |
| congenital heart disease | 20 mm Hg | 1.5549 | 4.74 (3.49-6.42) | <.001 |
| Valve disease | Yes/no | 1.3929 | 4.03 (2.86-5.67) | <.001 |
| Diabetes | Yes/no | 1.3857 | 4.00 (2.78-5.74) | <.001 |
| BMI | kg/m2 | 0.0578 | 1.06 (1.03-1.09) | <.001 |
| Valve disease and diabetes | Yes/no | -0.986 | 0.37 (0.18-0.78) | 0.009 |
| *OR indicates odds ratio; CI, confidence interval; LVH, left ventricular hypertrophy; CHD, congenital heart disease; and BMI, body mass index. Predicted probability of heart failure can be calculated as: p = 1/(1+exp(-xbeta)), where xbeta = Intercept + Sum (of regression coefficient*value of risk factor) | | | | |

Many of the risk factors included in this risk equation were not simulated in the diabetes model, therefore they could not be included in the model to predict CHD. We adjusted the baseline odds of CHD to reflect the expected prevalence of these symptoms in a UK population.

The proportion of the UK population with left ventricular hypertrophy was assumed to be 5% in line with previous analyses of the Whitehall II cohort (26). The heart rate for men was assumed to be 63.0bpm and for women 65.6bpm based on data from previous Whitehall II cohort analyses (27). The prevalence of congenital heart disease was estimated from an epidemiology study in the North of England. The study reports the prevalence of congenital heart disease among live births which was used to estimate the adult prevalence (28). This may over-estimate the prevalence, because the life expectancy of births with congenital heart disease is reduced compared with the general population. However, given the low prevalence it is unlikely to impact on the results. The prevalence of valve disease was estimated from the Echocardiographic Heart of England Screening study (29).

Using the estimated population values we adjusted the intercept values to account for the population risk in men and women. This resulted in a risk equation with age, systolic blood pressure, diabetes (diabetes diagnosis or HbA1c>6.5), and BMI in women to describe the risk of congestive heart failure for the policy analysis model.

## Microvascular Complications

The review of previous economic evaluations identified that the UKPDS data was commonly used to estimate the incidence of microvascular complications (17). This data has the advantage of being estimated from a UK diabetic population. Given that the events described in the UKPDS outcomes model are indicative of late stage microvascular complications, we did not believe it was necessary to seek an alternative model that would be representative of an impaired glucose tolerance population.

We adopted a simple approach to modelling microvascular complications. We used both versions of the UKPDS Outcomes model to estimate the occurrence of major events relating to these complications, including renal failure, amputation, foot ulcer, and blindness (11;18). These have the greatest cost and utility impact compared with earlier stages of microvascular complications, so are more likely to have an impact on the SPHR diabetes prevention outcomes.

As a consequence, we assumed that microvascular complications only occur in individuals with HbA1c>48 mmol/mol (6.5%). Whilst some individuals with hyperglycaemia (HbA1c>42 mmol/mol [6.0%]) may be at risk of developing microvascular complications, it is unlikely that they will progress to renal failure, amputation or blindness before a diagnosis of diabetes. Importantly, we did not assume that only individuals who have a formal diagnosis of diabetes are at risk of these complications. This allows us to incorporate the costs of undetected diabetes into the simulation.

The UKPDS includes four statistical models to predict foot ulcers, amputation with no prior ulcer, amputation with prior ulcer and a second amputation (18). In order to simplify the simulation of neuropathy outcomes we consolidated the models for first amputation with and without prior ulcer into a single equation. The parametric survival models were used to generate estimates of the cumulative hazard in the current and previous period. From which the probability of organ damage being diagnosed was estimated.

|  | $p\left( Death \right)=1-exp(H\left( t \right)-H\left( t-1 \right))$ |  |
| --- | --- | --- |

The functional form for the microvascular models included exponential and Weibull.

#### Retinopathy

We used the UKPDS outcomes model v2 to estimate the incidence of blindness in individuals with HbA1c>48 mmol/mol (6.5%) (18). The exponential model assumes a baseline hazard $\lambda$, which can be calculated from the model coefficients reported in Table 14 and the individual characteristics for $\boldsymbol{X}$.

$$\lambda=exp\left( \beta_{0}\boldsymbol{+X}\boldsymbol{\beta}_{\boldsymbol{k}} \right)$$

Table 15: Parameters of the UKPDS2 Exponential Blindness survival model

|  | Mean coefficient | Standard error | Modified mean coefficient |
| --- | --- | --- | --- |
| Lambda | -11.607 | 0.759 | -10.967 |
| Age at diagnosis | 0.047 | 0.009 | 0.047 |
| HbA1c | 0.171 | 0.032 | 0.171 |
| Heart rate | 0.080 | 0.039 |  |
| SBP | 0.068 | 0.032 | 0.068 |
| White Blood Count | 0.052 | 0.019 |  |
| CHF History | 0.841 | 0.287 | 0.841 |
| IHD History | 0.0610 | 0.208 | 0.061 |
| SBP Systolic Blood Pressure; CHF Congestive Heart Failure; IHD Ischaemic Heart Disease | | | |

The age at diagnosis coefficient was multiplied by age in the current year if the individual had not been diagnosed with diabetes, and by the age at diagnosis if the individual had received a diagnosis.

The expected values for the risk factors not included in the SPHR model (heart rate and white blood count) were taken from Figure 3 of the UKPDS publication in which these are described (18). Assuming these mean values, it was possible to modify the baseline risk without simulating heart rate and white blood cell count.

#### Neuropathy

We used the UKPDS outcomes model v2 to estimate the incidence of ulcer and amputation in individuals with HbA1c>48 mmol/mol (6.5%) (18). The parameters of the ulcer and first amputation models are reported in Table 15.

Table 16: Parameters of the UKPDS2 Exponential model for Ulcer, Weibull model for first amputation with no prior ulcer and exponential model for 1^st^ amputation with prior ulcer

|  | Ulcer | | 1^st^ Amputation no prior ulcer | | 1^st^ Amputation prior ulcer | | 2^nd^ Amputation | |
| --- | --- | --- | --- | --- | --- | --- | --- | --- |
|  | Logistic | | Weibull | | Exponential | | Exponential | |
|  | Mean | Standard error | Mean | Standard error | Mean | Standard error | Mean | Standard error |
| Lambda | -11.295 | 1.130 | -14.844 | 1.205 | -0.881 | 1.39 | -3.455 | 0.565 |
| Rho |  |  | 2.067 | 0.193 |  |  |  |  |
| Age at diagnosis | 0.043 | 0.014 | 0.023 | 0.011 | -0.065 | 0.027 |  |  |
| Female | -0.962 | 0.255 | -0.0445 | 0.189 |  |  |  |  |
| Atrial fibrillation |  |  | 1.088 | 0.398 |  |  |  |  |
| BMI | 0.053 | 0.019 |  |  |  |  |  |  |
| HbA1c | 0.160 | 0.056 | 0.248 | 0.042 |  |  | 0.127 | 0.06 |
| HDL |  |  | -0.059 | 0.032 |  |  |  |  |
| Heart rate |  |  | 0.098 | 0.050 |  |  |  |  |
| MMALB |  |  | 0.602 | 0.180 |  |  |  |  |
| PVD | 0.968 | 0.258 | 1.010 | 0.189 | 1.769 | 0.449 |  |  |
| SBP |  |  | 0.086 | 0.043 |  |  |  |  |
| WBC |  |  | 0.040 | 0.017 |  |  |  |  |
| Stroke History |  |  | 1.299 | 0.245 |  |  |  |  |

The exponential model assumes a baseline hazard $\lambda$, which can be calculated from the model coefficients reported in Table 15 and the individual characteristics for $\boldsymbol{X}$.

$$\lambda=exp\left( \beta_{0}\boldsymbol{+X\beta} \right)$$

The Weibull model for amputation assumes a baseline hazard:

$$h\left( t \right)=\rho t^{\rho-1}exp(\lambda)$$

where $\lambda$is also conditional on the coefficients and individual characteristics at time t.

The logistic model for ulcer is described below.

$$\Pr\left( y=1 | \mathbf{X} \right)=\frac{\exp(\mathbf{X}\boldsymbol{\beta})}{1+exp(\mathbf{X}\boldsymbol{\beta}))}$$

The ulcer and amputation models include a number of covariates that were not included in the simulation. As such it was necessary to adjust the statistical models to account for these measures. We estimated a value for the missing covariates and added the value multiplied by the coefficient to the baseline hazard.

The expected values for the risk factors not included in the SPHR model (heart rate, white blood count, micro-/macroalbuminurea, peripheral vascular disease and atrial fibrillation) were taken from Figure 3 of the UKPDS publication in which these are described (18). In the ulcer model we assumed that 2% of the population had peripheral vascular disease.

The amputation risk model with a history of ulcer was not included in the simulation, but was used to estimate an additional log hazard ratio to append onto the amputation model without a history of ulcer. The log hazard was estimated for each model assuming the same values for other covariates. The difference in the log hazard between the two models was used to approximate the log hazard ratio for a history of ulcer in the amputation model (10.241). The final model specifications are reported in Table 16.

Table 17: Coefficients estimates for Ulcer and 1^st^ Amputation

|  | Ulcer | | 1^st^ Amputation | | 2^nd^ Amputation | |
| --- | --- | --- | --- | --- | --- | --- |
|  | Logistic | | Weibull | | Exponential | |
|  | Mean | Standard error | Mean | Standard error | Mean | Standard error |
| Lambda | -11.276 | 1.13 | -13.954 | 1.205 | -3.455 | 0.565 |
| Rho |  |  | 2.067 | 0.193 |  |  |
| Age at Diagnosis | 0.043 | 0.014 | 0.023 | 0.011 |  |  |
| Female | -0.962 | 0.255 | -0.445 | 0.189 |  |  |
| BMI | 0.053 | 0.019 |  |  |  |  |
| HbA1c | 0.160 | 0056 | 0.248 | 0.042 | 0.127 | 0.06 |
| HDL |  |  | -0.059 | 0.032 |  |  |
| Stroke |  |  | 1.299 | 0.245 |  |  |
| Foot Ulcer |  |  | 10.241 |  |  |  |

#### Nephropathy

We used the UKPDS outcomes model v1 to estimate the incidence of renal failure in individuals with HbA1c>48 mmol/mol (6.5%) (11). Early validation analyses identified that the UKPDS v2 model substantially overestimated the incidence of renal failure in the SPHR model. The Weibull model for renal failure assumes a baseline hazard:

$$h\left( t \right)=\rho t^{\rho-1}exp(\lambda)$$

where $\lambda$is also conditional on the coefficients and individual characteristics at time t. The parameters of the renal failure risk model are reported in Table 17.

Table 18: Parameters of the UKPDS2 Weibull renal failure survival model

|  | Mean | Standard error |
| --- | --- | --- |
| Lambda | -10.016 | 0.939 |
| Shape parameter | 1.865 | 0.387 |
| SBP | 0.404 | 0.106 |
| BLIND History | 2.082 | 0.551 |

##

## Cancer

The conceptual model identified breast cancer and colorectal cancer risk as being related to BMI. However, these outcomes were not frequently included in previous cost-effectiveness models for diabetes prevention. Discussion with stakeholders identified the EPIC Norfolk epidemiology cohort study as a key source of information about cancer risk in a UK population. Therefore, we searched publications from this cohort to identify studies reporting the incidence of these risks. In order to obtain the best quality evidence for the relationship between BMI and cancer risk we searched for a recent systematic review and meta-analysis using key terms ‘Body Mass Index’ and ‘Cancer’, filtering for meta-analysis studies.

### Breast cancer

Incidence rates for breast cancer in the UK were estimated from the European Prospective Investigation of Cancer (EPIC) cohort. This is a large multi-centre cohort study looking at diet and cancer. In 2004 the UK incidence of breast cancer by menopausal status was reported in a paper from this study investigating the relationship between body size and breast cancer (30). The estimates of the breast cancer incidence in the UK are reported in Table 18.

Table 19: UK breast cancer incidence

|  | Number of Cases | Person Years | Mean BMI | Incidence Rate of per person-year | Standard error | Reference |
| --- | --- | --- | --- | --- | --- | --- |
| UK pre-menopause | 102 | 103114.6 | 24 | 0.00099 | 0.00009 | (30) |
| UK post-menopause | 238 | 84214.6 | 24 | 0.00283 | 0.00004 | (30) |

A large meta-analysis that included 221 prospective observational studies has reported relative risks of cancers per unit increase in BMI, including breast cancer by menopausal status (31). We included a risk adjustment in the model so that individuals with higher BMI have a higher probability of pre-and post-menopausal breast cancer (31). In the simulation we adjusted the probability of breast cancer according to the difference in the individual’s BMI and the average BMI reported in the EPIC cohort. The relative risk and confidence intervals per 5mg/m^2^ increase in BMI are reported in Table 19.

Table 20: Relative risk of Breast cancer by BMI

|  | Mean Relative risk | 2.5^th^ Confidence Interval | 97.5^th^ Confidence Interval | Reference |
| --- | --- | --- | --- | --- |
| UK pre-menopause | 0.89 | 0.84 | 0.94 | (31) |
| UK post-menopause | 1.09 | 1.04 | 1.14 | (31) |

### Colorectal cancer

Incidence rates for colorectal cancer in the UK were reported from the European Prospective Investigation of Cancer (EPIC) cohort. The UK incidence of colorectal cancer is reported by gender in a paper from this study investigating the relationship between body size and colon and rectal cancer (32). The estimates of the colorectal cancer incidence are reported in Table 20.

Table 21: UK colorectal cancer incidence

|  | Number of Cases | Person Years | Mean Age | Mean BMI | Incidence Rate of per person-year | Standard error | Reference |
| --- | --- | --- | --- | --- | --- | --- | --- |
| Male | 125 | 118468 | 53.1 | 25.4 | 0.00106 | 0.0001 | (32) |
| Female | 145 | 277133 | 47.7 | 24.5 | 0.00052 | 0.0002 | (32) |

The risk of colorectal cancer has been linked to obesity. We included a risk adjustment in the model to reflect observations that the incidence of breast cancer is increased in individuals with higher BMI. A large meta-analysis that included 221 prospective observational studies has reported relative risks of BMI and cancers, including colon cancer by gender (31). We selected linear relative risk estimates estimated from pooled European and Australian populations. In the simulation we adjusted the incidence of colorectal cancer by adjusting the probability of colorectal cancer by the difference in the individual’s BMI and the average BMI reported in the EPIC cohort. The relative risk and confidence intervals per 5mg/m^2^ increase in BMI are reported in Table 21.

Table 22: Relative risk of colon cancer by BMI

|  | Mean Relative risk | 2.5^th^ Confidence Interval | 97.5^th^ Confidence Interval | Reference |
| --- | --- | --- | --- | --- |
| UK pre-menopause | 1.21 | 1.18 | 1.24 | (31) |
| UK post-menopause | 1.04 | 1.00 | 1.07 | (31) |

##

## Osteoarthritis

Stakeholders suggested that diabetes and BMI should be included as independent risk factors for osteoarthritis. Osteoarthritis had not been included as a health state in previous cost-effectiveness models. The stakeholder group requested that BMI and diabetes be included as risk factors for osteoarthritis based on recent evidence (33). A search for studies using key words ‘Diabetes’, ‘Osteoarthritis’ and ‘Cohort Studies’ did not identify a UK based study with diabetes and body mass index included as independent covariates in the risk model. Therefore, the Italian study was used in the model.

A study from the Bruneck cohort, a longitudinal study of inhabitants of a town in Italy reported diabetes and BMI as independent risk factors for osteoarthritis (33).

The cohort may not be representative of a UK cohort. However, the individuals are from a European country, the study has a large sample size and has estimated the independent effects of BMI and diabetes on the risk of osteoarthritis. No UK based studies identified in our searches met these requirements. The data used to estimate the incidence of osteoarthritis is reported in Table 22. We did not identify any studies that described diabetes risk on a continuous scale.

Table 23: Incidence of osteoarthritis and estimated risk factors

|  | No cases | Person years | Mean BMI | Incidence rate | Standard error | Reference |
| --- | --- | --- | --- | --- | --- | --- |
| No diabetes | 73 | 13835 | 24.8 | 0.0053 | 0.0006 | (33) |
|  | Hazard ratio | 2.5th | 97.5th |  |  | Reference |
| HR Diabetes | 2.06 | 1.11 | 3.84 |  |  | (33) |
| HR BMI | 1.076 | 1.023 | 1.133 |  |  | (33)Personal communication |

## Depression

Depression was not included as a health state in previous cost-effectiveness models for diabetes prevention. However, a member of the stakeholder group identified that a relationship between diabetes and depression was included in the CORE diabetes treatment model (34). Therefore, the references used in this model were used.

Depression was included as a health state in the model. However, the severity of depression was not modelled. Some individuals enter the simulation with depression at baseline according to individual responses in the Health Survey for England 2014 questionnaire. Depression is described in the simulation as a chronic state from which individuals do not completely remit. We did not estimate the effect of depression on the longitudinal changes for BMI, glycaemia, SBP and cholesterol. As a consequence, it was not possible to relate the impact of depression to the incidence of diabetes and cardiovascular risk.

In the simulation, individuals can develop depression in any cycle of the model. The baseline incidence of depression among all individuals without a history of depression was estimated from a study examining the bidirectional association between depressive symptoms and type 2 diabetes (35). Although the study was not from a UK population, the US cohort included ethnically diverse men and women aged 45 to 84 years. We assumed that diagnosis of diabetes and/or CVD increased the incidence of depression in individuals who do not have depression at baseline. We identified a method for inflating risk of depression for individuals with diabetes from the US cohort study described above (35). The risk of depression in individuals who have had a stroke was also inflated according to a US cohort study (36). Odds of depression and odds ratios for inflated risk of depression due to diabetes or stroke are presented in Table 23.

Table 24: Baseline incidence of depression

| Baseline Risk of depression | | | |
| --- | --- | --- | --- |
|  | Mean | Standard error |  |
| Depression cases in NGT | 336 |  |  |
| Person years | 9139 |  |  |
| Odds of depression | 0.0382 | 0.002 |  |
| Log odds of depression | -3.266 |  |  |
| Inflated risk for Diabetes | | | |
|  | Mean | 2.5th CI | 97.5th CI |
| Odds ratio of diabetes | 1.52 | 1.09 | 2.12 |
| Log odds ratio of diabetes | 0.419 |  |  |
| Inflate risk of stroke | | | |
| Odds ratio of stroke | 6.3 | 1.7 | 23.2 |
| Log odds ratio stroke | 1.8406 |  |  |
| NGT Normal Glucose Tolerance | | | |

## Dementia

The risk dementia diagnosis is estimated from risk models estimated from the THIN database (37). The THIN dementia risk score uses data from The Health Improvement Network (THIN) database from across the UK. Routinely collected data was used to predict 5-year risk of recorded diagnosis of Dementia for those aged 60-79 and 80+. The sample size is large and the risk scores are representative of the United Kingdom and diagnosis practices between 2000-2011. The disadvantage of these risk scores are the relatively short follow-up of patients, the low predictive power of the older risk score, and narrow scope to predict dementia diagnosis but not dementia onset.

The parameters for the THIN 60-79 year old and 80-99 risk models are reported in Table 24.

Table 25: THIN dementia risk models

| THIN 60-79 Risk Score | | | THIN 80-99 Risk Score | | |
| --- | --- | --- | --- | --- | --- |
| Parameter label | mean | Standard error | Parameter label | mean | Standard error |
| Baseline hazard | 0.9969 |  | Baseline hazard | -0.9277 |  |
| Age | 0.2092 | 0.0047 | Age | 0.055 | 0.0041 |
| Age^2^ | -0.0034 | 0.0003 | Age^2^ | -0.005 | 0.0010 |
| Female | 0.1285 | 0.0278 | Female | 0.16 | 0.0286 |
| Calendar Year | 0.0448 | 0.0050 | Calendar Year | 0.074 | 0.0056 |
| Townsend quintile 2 | 0.0134 | 0.0390 | BMI | -0.05 | 0.0066 |
| Townsend quintile 3 | 0.1179 | 0.0392 | Anti-hypertensives | -0.249 | 0.0265 |
| Townsend quintile 4 | 0.2018 | 0.0402 | Systolic Blood Pressure | -0.006 | 0.0010 |
| Townsend quintile 5 | 0.2255 | 0.0447 | Lipid ratio | 0.042 | 0.0495 |
| BMI | -0.0616 | 0.0038 | Past Smoker | -0.178 | 0.0281 |
| BMI^2^ | 0.0025 | 0.0003 | Smoker | -0.134 | 0.0485 |
| Anti-hypertensives | -0.1320 | 0.0296 | Alcohol Porblems | 0.256 | 0.1352 |
| Past Smoker | -0.0679 | 0.0301 | Diabetes | 0.183 | 0.0413 |
| Smoker | -0.0866 | 0.0415 | Stroke | 0.242 | 0.0332 |
| Alcohol problems | 0.4435 | 0.0799 | Atrial Fibrillation | 0.057 | 0.0383 |
| Diabetes | 0.2867 | 0.0417 | Depression | 0.4 | 0.0332 |
| Depression | 0.8336 | 0.0325 | Anxiety | 0.136 | 0.0520 |
| Stroke | 0.5772 | 0.0394 | NSAIDs use | -0.157 | 0.0408 |
| Atrial Fibrillation | 0.2207 | 0.0514 | Aspirin use | 0.092 | 0.0281 |
| Aspirin use | 0.2528 | 0.0326 |  |  |  |

For the SPHR prevention model the 5-year Dementia risk was transformed into 1 year individual probabilities. The Dementia risk scores include fixed and time-varying patient characteristics. As a consequence, it is not possible to use standard methods of transforming probabilities over different time-horizons (38). We used a simple calibration technique to modify the baseline hazard to reflect simulated changes in the populations risk profile over 5 years. We calibrate the simulated 5 year incidence of dementia against the predicted incidence for each age group in the THIN database (37).

For each risk model we simulated 20,000 randomly sampled patients aged 60-79 and 80-95 in 50 model runs. For each sample we repeated simulations multiple times, in each simulation the baseline hazard was adjusted until the incidence of Dementia equalled the THIN risk score prediction based on baseline characteristics. The baseline hazard adjustment was estimated by averaging the adjustments needed for each of the 20 simulation runs to match the THIN prediction. The calibration was designed to calibrate to the predicted incidence, rather than the reported incidence from the THIN dataset to account for any differences in the baseline characteristics of the THIN data and HSE sample. For example, the mean age for the development cohort of the 60-79 model were 65.6, whereas the mean simulated ages was 70. Age is an important predictor of Dementia incidence so it is important to adjust for differences in baseline age between the observed and simulated data.

The THIN database reports a crude incidence of 1.88 per 1,000 persons years for 60-79 and 16.53 per 1,000 person years for ages 80-99 (column 2 Table 25). The 5 year risk score for individuals sampled from the Health Survey for England reports a 5 year incidence of X (column 3 Table 25). Using the adjustment factor identified by calibration (column 5 Table 25), we simulated a 5 year incidence of X and Y (column 4 Table 25). The adjustment factor is applied to the baseline hazards of the THIN dementia risk scores.

Table 26: Dementia incidence rates used to derive the adjustment factor

|  | 5 year crude incidence from QResearch | 5 year risk score for Health Survey for England population | 5 year simulated incidence with adjustment factor | Adjustment factor (SE) |
| --- | --- | --- | --- | --- |
| THIN risk model 60-79 | 0.00188 | 0.00255 | 0.00255 | 7.628 (0.104) |
| THIN risk model 80+ | 0.01653 | 0.01523 | 0.01510 | 4.557 (0.020) |

### Dementia Diagnosis

A Swedish registry reporting MMSE scores at dementia diagnosis was identified (39). This study reported a mean MMSE score at diagnosis of 21.2 (SD 5.2). This data was used to generate MMSE scores at diagnosis in the model because it reflects cognitive function in a cohort diagnosed in routine care. In order to generate heterogeneity in cognitive function at diagnosis a Gamma distribution was fitted to this mean and standard distribution and patients MMSE score at diagnosed was sampled from this distribution. Sampled value outside the limits of the MMSE score, assuming a maximum score at diagnosis of 25, were re-sampled from a uniform distribution within these limits. The resulting distribution was compared against summary data from CFAS for an incident cohort to validate the simulated MMSE scores against this dataset (40).


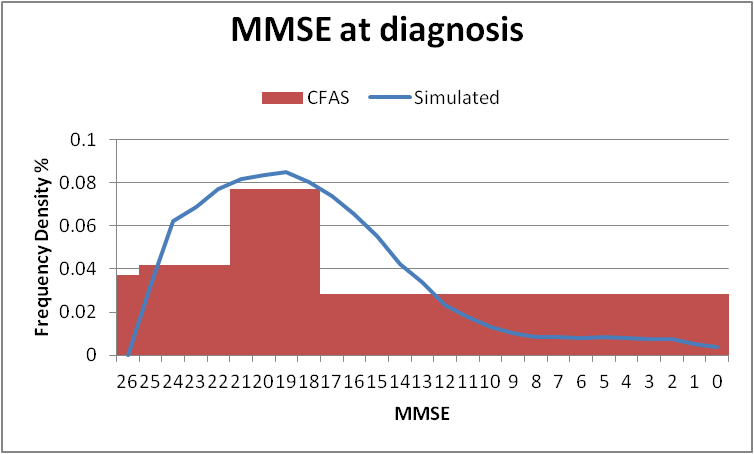


### Disease Progression

Dementia disease progression was characterized by a deterioration in MMSE score. This relatively simple characterization of the disease is sufficient to capture major cost escalations and quality of life deterioration. A more complex structure that explicity models insitutionalisation was considered (41). However, it was concluded that the data on insitutionalisation was out of date. Therefore, the modelling structure was aligned to the most up to date cost estimates for dementia (42).

Changes in MMSE score over time were estimated using data from a recent cost-effectiveness model for Donepezil (43). Although the data are from Canada and are relatively old, the sample size is large and model specification allows for detailed characterization of MMSE decline. The regression allows rate of change in MMSE to be conditional on age at baseline and includes splines to describe a different rate of change at different levels of MMSE. For example, the decline in MMSE slows as the score declines below 9.

## Mortality

### Cardiovascular Mortality

Cardiovascular mortality is included as an event within the QRISK2 (20) and the probability of subsequent cardiovascular events obtained from an HTA assessing statins (7), as described in the Cardiovascular disease section above.

### Cancer Mortality

Cancer mortality rates were obtained from the Office of National statistics (7;44). The ONS report one and five year net survival rates for various cancer types, by age group and gender. Net survival was an estimate of the probability of survival from the cancer alone. It can be interpreted as the survival of cancer patients after taking into account the background mortality that the patients would have experienced if they had not had cancer.

The age-adjusted 5-year survival rate for breast cancer and colorectal cancer were used to estimate an annual risk of mortality assuming a constant rate of mortality. We assume that the mortality rate does not increase due to cancer beyond 5 years after cancer diagnosis. The five year survival rate for breast cancer is 84.3%, which translated into a 3.37% annual probability of death from breast cancer. The five year survival rate for persons with colorectal cancer is 55.3%, which translated into a 11.16% annual probability of death from colorectal cancer.

### Other cause Mortality (including diabetes and Dementia risk)

Other cause mortality describes the risk of death from any cause except CVD, and cancer. All-cause mortality rates by age and sex were extracted from the 2014 Office of National Statistics life tables (5;7). The mortality statistics report the number of deaths by ICD codes for 5-year age groups. We subtracted the number of cardiovascular disease,diabetes, dementia, breast and colorectal cancer related deaths from the all-cause mortality total to estimate other cause mortality rates by age and sex (Table 21).

Table 27: All cause and derived other cause mortality from the Office of National statistics

|  | All cause | All cause | Other cause | Other cause |  | All cause | All cause | Other cause | Other cause |
| --- | --- | --- | --- | --- | --- | --- | --- | --- | --- |
|  | Men | Women | Men | Women |  | Men | Women | Men | Women |
| 1 | 0.0003 | 0.0003 | 0.0003 | 0.0003 | 51 | 0.0030 | 0.0021 | 0.0022 | 0.0015 |
| 2 | 0.0002 | 0.0001 | 0.0002 | 0.0001 | 52 | 0.0030 | 0.0021 | 0.0022 | 0.0015 |
| 3 | 0.0001 | 0.0001 | 0.0001 | 0.0001 | 53 | 0.0030 | 0.0021 | 0.0022 | 0.0015 |
| 4 | 0.0001 | 0.0001 | 0.0001 | 0.0001 | 54 | 0.0030 | 0.0021 | 0.0022 | 0.0015 |
| 5 | 0.0001 | 0.0001 | 0.0001 | 0.0001 | 55 | 0.0030 | 0.0021 | 0.0022 | 0.0015 |
| 6 | 0.0001 | 0.0001 | 0.0001 | 0.0001 | 56 | 0.0030 | 0.0021 | 0.0022 | 0.0015 |
| 7 | 0.0001 | 0.0001 | 0.0001 | 0.0001 | 57 | 0.0030 | 0.0021 | 0.0022 | 0.0015 |
| 8 | 0.0001 | 0.0001 | 0.0001 | 0.0001 | 58 | 0.0030 | 0.0021 | 0.0022 | 0.0015 |
| 9 | 0.0001 | 0.0001 | 0.0001 | 0.0001 | 59 | 0.0030 | 0.0021 | 0.0022 | 0.0015 |
| 10 | 0.0001 | 0.0001 | 0.0001 | 0.0001 | 60 | 0.0030 | 0.0021 | 0.0022 | 0.0015 |
| 11 | 0.0001 | 0.0001 | 0.0001 | 0.0001 | 61 | 0.0030 | 0.0021 | 0.0022 | 0.0015 |
| 12 | 0.0001 | 0.0001 | 0.0001 | 0.0001 | 62 | 0.0030 | 0.0021 | 0.0022 | 0.0015 |
| 13 | 0.0001 | 0.0001 | 0.0001 | 0.0001 | 63 | 0.0030 | 0.0021 | 0.0022 | 0.0015 |
| 14 | 0.0001 | 0.0001 | 0.0001 | 0.0001 | 64 | 0.0030 | 0.0021 | 0.0022 | 0.0015 |
| 15 | 0.0001 | 0.0001 | 0.0001 | 0.0001 | 65 | 0.0030 | 0.0021 | 0.0022 | 0.0015 |
| 16 | 0.0002 | 0.0001 | 0.0002 | 0.0001 | 66 | 0.0030 | 0.0021 | 0.0022 | 0.0015 |
| 17 | 0.0003 | 0.0001 | 0.0003 | 0.0001 | 67 | 0.0030 | 0.0021 | 0.0022 | 0.0015 |
| 18 | 0.0004 | 0.0002 | 0.0004 | 0.0002 | 68 | 0.0030 | 0.0021 | 0.0022 | 0.0015 |
| 19 | 0.0005 | 0.0002 | 0.0004 | 0.0002 | 69 | 0.0030 | 0.0021 | 0.0022 | 0.0015 |
| 20 | 0.0004 | 0.0002 | 0.0004 | 0.0002 | 70 | 0.0030 | 0.0021 | 0.0022 | 0.0015 |
| 21 | 0.0004 | 0.0002 | 0.0004 | 0.0002 | 71 | 0.0030 | 0.0021 | 0.0022 | 0.0015 |
| 22 | 0.0004 | 0.0002 | 0.0004 | 0.0002 | 72 | 0.0030 | 0.0021 | 0.0022 | 0.0015 |
| 23 | 0.0005 | 0.0002 | 0.0005 | 0.0002 | 73 | 0.0030 | 0.0021 | 0.0022 | 0.0015 |
| 24 | 0.0005 | 0.0002 | 0.0005 | 0.0002 | 74 | 0.0030 | 0.0021 | 0.0022 | 0.0015 |
| 25 | 0.0005 | 0.0002 | 0.0005 | 0.0002 | 75 | 0.0030 | 0.0021 | 0.0022 | 0.0015 |
| 26 | 0.0006 | 0.0002 | 0.0006 | 0.0002 | 76 | 0.0030 | 0.0021 | 0.0022 | 0.0015 |
| 27 | 0.0006 | 0.0003 | 0.0006 | 0.0002 | 77 | 0.0030 | 0.0021 | 0.0022 | 0.0015 |
| 28 | 0.0006 | 0.0003 | 0.0006 | 0.0003 | 78 | 0.0030 | 0.0021 | 0.0022 | 0.0015 |
| 29 | 0.0006 | 0.0003 | 0.0006 | 0.0003 | 79 | 0.0030 | 0.0021 | 0.0022 | 0.0015 |
| 30 | 0.0007 | 0.0003 | 0.0006 | 0.0003 | 80 | 0.0030 | 0.0021 | 0.0022 | 0.0015 |
| 31 | 0.0007 | 0.0004 | 0.0007 | 0.0003 | 81 | 0.0030 | 0.0021 | 0.0022 | 0.0015 |
| 32 | 0.0007 | 0.0004 | 0.0007 | 0.0003 | 82 | 0.0030 | 0.0021 | 0.0022 | 0.0015 |
| 33 | 0.0008 | 0.0005 | 0.0007 | 0.0004 | 83 | 0.0030 | 0.0021 | 0.0022 | 0.0015 |
| 34 | 0.0008 | 0.0005 | 0.0008 | 0.0004 | 84 | 0.0030 | 0.0021 | 0.0022 | 0.0015 |
| 35 | 0.0010 | 0.0005 | 0.0009 | 0.0004 | 85 | 0.0030 | 0.0021 | 0.0022 | 0.0015 |
| 36 | 0.0010 | 0.0006 | 0.0009 | 0.0005 | 86 | 0.0030 | 0.0021 | 0.0022 | 0.0015 |
| 37 | 0.0011 | 0.0006 | 0.0010 | 0.0005 | 87 | 0.0030 | 0.0021 | 0.0022 | 0.0015 |
| 38 | 0.0012 | 0.0007 | 0.0011 | 0.0006 | 88 | 0.0030 | 0.0021 | 0.0022 | 0.0015 |
| 39 | 0.0013 | 0.0008 | 0.0012 | 0.0006 | 89 | 0.0030 | 0.0021 | 0.0022 | 0.0015 |
| 40 | 0.0015 | 0.0008 | 0.0012 | 0.0006 | 90 | 0.0030 | 0.0021 | 0.0022 | 0.0015 |
| 41 | 0.0016 | 0.0009 | 0.0013 | 0.0007 | 91 | 0.0030 | 0.0021 | 0.0022 | 0.0015 |
| 42 | 0.0016 | 0.0010 | 0.0013 | 0.0008 | 92 | 0.0030 | 0.0021 | 0.0022 | 0.0015 |
| 43 | 0.0018 | 0.0011 | 0.0015 | 0.0008 | 93 | 0.0030 | 0.0021 | 0.0022 | 0.0015 |
| 44 | 0.0019 | 0.0012 | 0.0016 | 0.0009 | 94 | 0.0030 | 0.0021 | 0.0022 | 0.0015 |
| 45 | 0.0022 | 0.0013 | 0.0017 | 0.0010 | 95 | 0.0030 | 0.0021 | 0.0022 | 0.0015 |
| 46 | 0.0022 | 0.0014 | 0.0018 | 0.0010 | 96 | 0.0030 | 0.0021 | 0.0022 | 0.0015 |
| 47 | 0.0024 | 0.0016 | 0.0019 | 0.0011 | 97 | 0.0030 | 0.0021 | 0.0022 | 0.0015 |
| 48 | 0.0025 | 0.0017 | 0.0020 | 0.0012 | 98 | 0.0030 | 0.0021 | 0.0022 | 0.0015 |
| 49 | 0.0028 | 0.0018 | 0.0023 | 0.0013 | 99 | 0.0030 | 0.0021 | 0.0022 | 0.0015 |
| 50 | 0.0030 | 0.0021 | 0.0022 | 0.0015 | 100 | 0.0030 | 0.0021 | 0.0022 | 0.0015 |

The rate of other cause mortality by age and sex was treated as the baseline hazard. Following input from stakeholders, an increased risk of mortality was assigned to individuals with diabetes using data from a published meta-analysis (45). This study used data from 820,900 people from 97 prospective studies to calculate hazard ratios for cause-specific death, according to baseline diabetes status (45). Cause of death was separated into vascular disease, cancer and other cause mortality. From this study we estimated that individuals with a diagnosis of diabetes have a fixed increased risk of other cause mortality (Hazard ratio 1.8 (95% CI 1.71-1.9)). The estimates reported in the meta-analysis include increased risk of death from renal disease, therefore mortality from renal disease was not simulated separately to avoid double counting of benefits.

Mortality risk increases with the onset of dementia. As a consequence, all cause mortality was inflated after diagnosis of dementia. The hazard ratio of mortality was estimated from analysis of two United States cohorts (46). Participants were recruited to the studies without known dementia at baseline and received annual clinical evaluation and brain donation at death. The analysis included 2566 persons over 8 years and found a hazard ratio of death with all dementia of 4.54 (CI 3.54-5.83) for ages 75-84 and 2.77 (CI 2.37-3.23) for ages 85 and older . These hazard ratios were applied in the model to all cause mortality to describe mortality at younger ages 60-84 and older 85+ ages.

Mortality rates for individuals with diabetes and undiagnosed diabetes are also at increased risk of mortality, which is applied to all cause mortality. Given the correlation between risk factors of diabetes and dementia, and the high prevalence of multi-comorbidities in later life, it is necessary to adjust mortality risk for individuals with both dementia and diabetes. We believe that applying both mortality hazard ratios would over-estimate the mortality burden in these patients. In the model the higher hazard ratio for Dementia is applied.

# Direct Health Care Costs

At any given time period of the model individuals can have multiple health complications that incur direct healthcare costs. Some of the health states are mutually exclusive; however an individual can accrue multiple complications within the model. Each health state is associated with an average cost, which is accrued by all individuals for every time period for which the state is indicated. Resource use for each comorbidity is added together and no savings are assumed to be made from the use of the same resources for two or more comorbidities for an individual.

In some instances we have adopted costs and prices from old studies. We have inflated all prices and costs to 2014/15 prices using inflation indices reported in the Personal Social Services Research Unit (PSSRU) (47). This documents health related inflation up to 2013/14 prices. The retail price index was used to inflate costs to 2014/15 prices.

Primary care and community care costs were sought from the Personal Social Services Research Unit (PSSRU) (47), and secondary care costs from UK reference costs (48). Drug costs were obtained from the British National Formulary (49). In most instances costs for long term health outcomes were sought from recent Health Technology Appraisals as this was thought to be the best source of evidence for costs and resource use by disease area in the UK. If an HTA appraisal was not identified, searches for good quality cost-effectiveness analyses for the relevant disease area were conducted to identify the appropriate UK costs.

## GP attendance

The costs of each visit to a General Practitioner were estimated at £46.95 from the Personal Social Services Research Unit (PSSRU) (47).

Diabetes diagnosis incurred a cost of £14 in line with costs used for a previous evaluation of a Diabetes Prevention Programme (3).

Recent guidelines for hypertension have recommended that hypertension be confirmed with ambulatory blood pressure monitoring (ABPM) (13). The cost of ABPM assessment is included in the cost of diagnosis (£53.40) (50), however, we assume that the test does not alter the initial diagnosis.

The cost of identifying individuals to receive statins is assumed to be negligible because cases are detected using existing cardiovascular risk programmes used by the GP.

## Diabetes

We were advised by stakeholders to model a simplified diabetes treatment pathway. It was recommended that a single annual cost of prescriptions be applied to all patients diagnosed with diabetes. Initially we explored this as an option but concluded that the timing of more costly treatments for type 2 diabetes is important because treatment costs will be discounted. The model assesses interventions that lower HbA1c and so have the potential to impact on the level of treatment required.

We decided to implement a three stage treatment regimen as a trade-off between model simplicity and capturing key cost differences between the interventions. At diagnosis all patients are prescribed low cost treatments, such as Metformin and Sulfonylurea. We chose Metformin, 500mg/day to describe the average cost of these medications. If HbA1c increases above a threshold the individual is prescribed the more expensive Gliptins in addition to Metformin. The individual continues to receive Metformin plus Gliptins for a period of time until they require insulin. A summary of unit costs used for diabetes maintenance is detailed in Table 27.

Table 28: Unit costs used for diabetes maintenance

| **Resource** | Unit cost | Standard error | Source |
| --- | --- | --- | --- |
| Nurse at GP | £25.52 | 2.5 | (47) |
| Health care assistant | £3.40 | 0.34 | (47) |
| Urine sample | £1 | 0.1 | (48) |
| Eye screening | £24.31 | 5.86 | (51) |
| HbA1c | £3 | 0.3 | (48) |
| Lipids | £1 | 0.1 | (48) |
| Liver function | £1 | 0.1 | (48) |
| B12 | £1 | 0.1 | (48) |

### Metformin Monotherapy

Cost estimates from the British National Formulary indicate that the cost of Metformin is approximately £19 per annum, using a combination of standard and modified release tablets (49). The use of blood glucose self-monitoring strips was described in a recent UK based study in which 36% of patients used monitoring strips at a mean weekly consumption of 3.1 (52) for individuals prescribed Metformin only, at a cost of 20p per strip as reported in the BNF.

Other resource use costs and resource utilisation assumptions for diabetics receiving Metformin monotherapy are detailed in Table 28.

Table 29: Drug costs and resource utilisation costs for low cost diabetes monotherapy

| Resource | Assumption for costs | Unit cost | Source | Inflation | Annual utilisation | Source | Cost per year |
| --- | --- | --- | --- | --- | --- | --- | --- |
| Metformin | 500mg *bid* standard (85% of patients) or modified release (15%) tablets | £18.83 per annum | (49) | 1 | 1 | Assumption | £18.83 |
| Nurse at GP | Nurse advanced per surgery consultation with qualifications | £25.52 | (47) | 1 | 1 | Stakeholder workshop | £25.52 |
| Health care assistant | Clinical support worker patient work 10 mins | £3.40 | (47) | 1 | 1 | Stakeholder workshop | £3.40 |
| Urine sample | Biochemistry | £1 | (48) | 1 | 3 | Stakeholder workshop | £1 |
| Eye screening | Optometrist test 2006 price | £18.39 | (51) | 1.322 | 1 | Stakeholder workshop | £24.31 |
| HbA1c | Haematology | £3 | (48) | 1 | 1 | Stakeholder workshop | £3.00 |
| Lipids | Chemistry | £1 | (48) | 1 | 1 | Stakeholder workshop | £1.00 |
| Liver function | Chemistry | £1 | (48) | 1 | 1 | Stakeholder workshop | £1.00 |
| B12 | Chemistry | £1 | (48) | 1 | 1 | Stakeholder workshop | £1.00 |
|  | | | | | | | £79.06 |

The cost of diabetes in the year after diagnosis is assumed to be greater than subsequent years because the individual will receive more contact time whilst their diabetes is being controlled. The additional costs of diabetes in the year after diagnosis are reported in Table 29.

Table 30: Drug costs and resource utilisation costs for the first year after diabetes diagnosis

| Resource | Assumption for costs | Unit cost | Source | Inflation | Annual utilisation | Source | Cost per year |
| --- | --- | --- | --- | --- | --- | --- | --- |
| Nurse at GP | Nurse advanced per surgery consultation with qualifications | £25 | (47) | 1 | 1 | Stakeholder workshop | £51.03 |
| Health care assistant | Clinical support worker patient work 10 mins | £3.40 | (47) | 1 | 2 | Stakeholder workshop | £6.80 |
| Urine sample | Biochemistry | £1 | (48) | 1 | 2 | Stakeholder workshop | £2 |
| HbA1c | Haematology | £3 | (48) | 1 | 2 | Stakeholder workshop | £6.00 |
| Lipids | Chemistry | £1 | (48) | 1 | 2 | Stakeholder workshop | £2.00 |
| Liver function | Chemistry | £1 | (48) | 1 | 2 | Stakeholder workshop | £2.00 |
| B12 | Chemistry | £1 | (48) | 1 | 2 | Stakeholder workshop | £2.00 |
| Smoking Cessation | Nicotine replacement therapy | £103 | (47) | 1 | 0.3* | Stakeholder workshop | £30.90 |
|  | | | | | | | £103 |
| * Assumed 20% smoking prevalence and 50% uptake of smoking cessation services | | | | | | |  |

### Metformin plus Gliptins

Simulated individuals experience an annual increase in HbA1c. Gillett et al. (2012) assume that individuals switch to dual treatment if HbA1c increases above 7.4% (53). Within the model, the individual is switched to a dual treatment in the first annual cycle in which HbA1c exceeds 7.4%. For costing purposes the second drug to be added to Metformin was Sitagliptin, which is reported in the British National Formulary to cost £1.21 per day (49). Belsey et al. (2009) report that 48% of patients used monitoring strips at a mean weekly consumption of 3.3 (52). Table 30 reports the other resource use costs and utilisation assumptions for diabetics receiving Metformin plus Gliptins.

Table 31: Drug costs and resource utilisation costs for Metformin and Gliptins

| Resource | Assumption for costs | Unit cost | Source | Inflation | Annual utilisation | Source | Cost per year |
| --- | --- | --- | --- | --- | --- | --- | --- |
| Sitagliptin | 100mg per day | £1.21 | (49) | 1 | 360 | Assumption | £434 |
| Metformin | 500mg *bid* standard (85% of patients) or modified release (15%) tablets | £18.83 per annum | (49) | 1 | 1 | Assumption | £18.83 |
| Self-monitoring strips | 50 strip pack Active® | £0.20 | (49) | 1 | 82.20 | (52) | £16.36 |
| Nurse at GP | Nurse advanced per surgery consultation with qualifications | £25.52 | (47) | 1 | 1 | Stakeholder workshop | £25.52 |
| Health care assistant | Clinical support worker patient work 10 mins | £3.40 | (47) | 1 | 1 | Stakeholder workshop | £3.40 |
| Urine sample | Biochemistry | £1 | (48) | 1 | 1 | Stakeholder workshop | £1 |
| Eye screening | Optometrist test 2006 price | £18.39 | (51) | 1.322 | 1 | Stakeholder workshop | £24.31 |
| HbA1c | Haematology | £3 | (48) | 1 | 1 | Stakeholder workshop | £3.00 |
| Lipids | Chemistry | £1 | (48) | 1 | 1 | Stakeholder workshop | £1.00 |
| Liver function | Chemistry | £1 | (48) | 1 | 1 | Stakeholder workshop | £1.00 |
| B12 | Chemistry | £1 | (48) | 1 | 1 | Stakeholder workshop | £1.00 |
|  | | | | | | | £529 |

### Insulin plus Oral Anti-diabetics

The second major treatment change is assumed to be initiation of insulin. Gillett et al. (2012) assumed that individuals switch to insulin if HbA1c increases above 8.5% (53). Within the model the individual is switched to insulin in the first annual cycle at which HbA1c exceeds 8.5%. The insulin Glargine was chosen to represent insulin treatment in the UK and is consistent with Gillett et al. (2012) (53). The total resource use and costs of this health state are reported in Table 31 &

Table 32.

Table 32: Costs of insulin treatment

|  | Price | Source |
| --- | --- | --- |
| Glargine | £628.44 | (54)(2006 prices) |
| Oral anti-diabetics | £43.68 | (54) (2006 prices) |
| Reagent test strips | £221.43 | (54) (2006 prices) |
| Hypoglycaemic rescue | £23.43 | (54) (2006 prices) |
| Pen delivery devices | £54.79 | (54) (2006 prices) |
| Sharps | £68.82 | (54) (2006 prices) |
| Total cost per year | £1,013.51 |  |

Table 33: Drug costs and resource utilisation costs for insulin and oral anti-diabetics

| Resource | Assumption for costs | Unit cost | Source | Inflation (2013) | Annual utilisation | Source | Cost per year |
| --- | --- | --- | --- | --- | --- | --- | --- |
| Insulin treatment costs | Total annual cost | £1,013.51 | (54) | 1.322 | NA | N/A | £1376 |
| Nurse at GP | Nurse advanced per surgery consultation with qualifications | £25.52 | (47) | 1 | 3 | Stakeholder workshop | £76.55 |
| Health care assistant | Clinical support worker patient work 10 mins | £3.40 | (47) | 1 | 3 | Stakeholder workshop | £10.21 |
| Urine sample | Biochemistry | £1 | (48) | 1 | 3 | Stakeholder workshop | £3.00 |
| Eye screening | Optometrist test 2006 price | £18.39 | (47) | 1.322 | 1 | Stakeholder workshop | £24.31 |
| HbA1c | Haematology | £3 | (48) | 1 | 3 | Stakeholder workshop | £9.00 |
| Lipids | Chemistry | £1 | (48) | 1 | 3 | Stakeholder workshop | £3.00 |
| Liver function | Chemistry | £1 | (48) | 1 | 3 | Stakeholder workshop | £3.00 |
| B12 | Chemistry | £1 | (48) | 1 | 3 | Stakeholder workshop | £3.00 |
|  | | | | | | | £1503 |

## Statins

We assumed that individuals who are prescribed statins receive a daily dose of 40mg of generic Simvastatin. The British National Formulary reports a cost of approximately 7p per day (49). The individual remains on statins for the rest of their life. Table 33 reports the derived annual costs for statins. We assumed that individual’s cholesterol is monitored whilst on statins and patients receive two lipid tests per year. The cost of GP attendance was not included in the cost of statins to avoid double counting of GP attendance.

Table 34: Annual treatment costs of statins

|  | Assumption for costs | Unit cost | Source | Inflation | Annual utilisation | Cost per year |
| --- | --- | --- | --- | --- | --- | --- |
| Statins | Simvastatin 20mg | £0.0728 | (49) | 1 | 360 | £26.59 |
| Statins | Lipid tests | £1 | (48) | 1 | 2 | £2 |
|  |  |  |  |  |  | £28.59 |

## Anti-hypertensives

A search of the literature did not identify any recent publications of anti-hypertensive prescriptions in the UK. As a consequence the best estimates of cost of anti-hypertensive treatment dated from 2004. These were inflated to current prices (47). Due to the number of different anti-hypertensive treatments available and possibilities for combination therapies, using the cost from this study of prescriptions was preferred to using costs directly from the BNF.

Table 35: Annual cost of anti-hypertensive prescription expenditure per patient

|  | Price | Inflation | Cost per year | Standard error | Source |
| --- | --- | --- | --- | --- | --- |
| Anti-hypertensive prescriptions | £144 | 1.322 | £195.94 | 19.59 | (55) |

## Cardiovascular Events

Costs for coronary heart disease disease were obtained from a 2009 HTA for high dose lipid-lowering therapy unless otherwise stated (12). The costs of stroke were obtained from a study estimating costs from the Oxford vascular cohort (56). Table 35 describes the costs and resource use assumptions that were used for this study. It also reports the health states to which we have applied each cost in the model. The costs of congestive heart failure were estimated from the UKPDS costing study for complications related to diabetes (57). The unit costs for cardiovascular events are detailed in Table 36.

Table 36: Resources use assumptions and costs for cardiovascular outcomes

|  | **Resource assumptions** | **Cost (2009)** | **Cost (2014/15)** | **Health States applied** |
| --- | --- | --- | --- | --- |
| Unstable Angina year 1 | Secondary care costs: 100% hospitalisation, 50% revascularisation procedure, three outpatient appointments).  Primary care costs (three GP visits) and medications. | £3880 | £4,674 | UANG1 |
| MI year 1 | Secondary care costs: 100% hospitalisation,  50% revascularisation procedure, three outpatient appointments).  Primary care costs (three GP visits) and medications. | £3996 | £4,813 | MI1 |
| Subsequent ACS care costs | Secondary care costs (one outpatient appointment).  Primary care costs (three GP visits) and medications. | £340 | £410 | SANG, UANG, MI |
| Stroke year 1 | Costs of first year post stroke (56) | £10,524 | £12,677 | STRO1 |
| Stroke subsequent costs | Average costs in years 2-5 following stroke (56). | £1,444 | £1,740 | STRO2 |
| Transient Ischemic Attack | Hopsital costs from 5 year study | £2,260 | £2,723 | TIA |
| Fatal CHD | Palmer et al. (58). Assumed that 50% of fatalities incurred cost. | £592 | £713 |  |
| Fatal non cardio- vascular event | Youman et al. (59). Assumed 50% fatalities incurred cost. | £3688 | £4,443 |  |
|  | **Source** | **Cost (2012)** | **Cost (2014/15)** |  |
| Congestive heart failure year 1 | UKPDS (57) | £3,191 | £3,091 |  |
| Congestive heart failure subsequent years | UKPDS (57) | £1,473 | £1,818 |  |

Table 37: Unit costs for Cardiovascular cost estimates taken from HTA report (12)

| Unit Cost | Mean | Inflation | Mean (2014/15) | Standard error | Distribution |
| --- | --- | --- | --- | --- | --- |
| Unstable Angina hospital: EB05SZ | £1059 | 1.2045 | £1275 | 127.6 | GAMMA |
| Revasc. Hospital mixture of HRG codes | £5011.81 | 1.2045 | £6037 | 604 | GAMMA |
| MI Hospital: EB107 | £1290.88 | 1.2045 | £1555 | 156 | GAMMA |
| First Outpatient | £137.28 | 1.2045 | £165 | 16.5 | GAMMA |
| Subsequent appointment | £91.37 | 1.2045 | £110 | 11.0 | GAMMA |
| GP visit year1 | £102 | 1.2045 | £123 |  | CONSTANT |
| GP visit year 2 | £91.37 | 1.2045 | £110 |  | CONSTANT |
| Fatal CHD (Palmer (58) Inflated) | £591.52 | 1.2045 | £713 | 71 | GAMMA |
| Fatal stroke (Youman (59) inflated) | £3688.23 | 1.2045 | £4443 | 444.3 | GAMMA |
| Glytrin Spray | £10.47 | 1.2045 | £12.61 |  | CONSTANT |
| Isosorbide mononitrate | £11.24 | 1.2045 | £13.54 |  | CONSTANT |
| Verapamil | £41.98 | 1.2045 | £50.57 |  | CONSTANT |
| Atenolol | £30.24 | 1.2045 | £36.42 |  | CONSTANT |
| Aspirin | £6.65 | 1.2045 | £8.01 |  | CONSTANT |
| Ramipril | £75.09 | 1.2045 | £90.45 |  | CONSTANT |
| ARB | £210.27 | 1.2045 | £253 |  | CONSTANT |
| Clopidogrel | £460.27 | 1.2045 | £554 |  | CONSTANT |

## Renal Failure

The cost of renal failure was estimated for the UK using relevant published studies. A recent costing study reported the costs of dialysis types (60). The prevalence of dialysis and transplants were taken from a second study reporting the prevalence of renal failure in the UK in 2008 (61). The cost of renal transplantation was taken from a costing study investigating the cost-effectiveness of renal transplantation (62). The overall cost was estimated as a weighted average of the treatment outcomes. All costs were inflated to 2014/15 prices.

Table 38: Unit costs for renal failure

|  | Cost (£) | Source | Inflation | Cost (2014/15) | Standard error | Proportion |
| --- | --- | --- | --- | --- | --- | --- |
| Haemodialysis with overheads | 34,236 | (60) | 1.2282 | £42,049 | 4204.9 | 0.469 |
| Automated peritoneal dialysis (APD) | 22,160 | (60) | 1. 2282 | £27,217 | 2721.7 | 0.045* |
| Continuous ambulatory peritoneal dialysis (CAPD) | 16,074 | (60) | 1. 2282 | £19,742 | 1974.2 | 0.045* |
| Transplant | 17,000 | (62) | 1.3918 | £23,660 | 2366 | 0.442 |
| Immunosuppressants annual cost | 5000 | (62) | 1. 39184 | £6,959 | 695.9 |  |
| * Assumed 50% split of peritoneal dialysis types | | | | | | |

## Foot Ulcers

A search of the literature did not identify any studies for foot ulcer for the UK or a health system comparable to the UK. The cost of foot ulcers was estimated from a US Cost of Illness study (63). We acknowledge that this is a limitation of the analysis, because US costs may not be representative of care in the UK. The costs were converted from dollars to pounds using Purchasing Power Parities reported by the OECD (64). The costs were also inflated to UK 2014/15 prices.

Table 39: Estimated cost of foot ulcers

| Resource component | Not Infected | With Cellulitis | With Osteomyelitis |
| --- | --- | --- | --- |
| Prevalence | 0.874 | 0.09 | 0.036 |
| Mean cost per patient | $178.97 | $472.73 | $876.52 |
| Mean cost per patient (2014/15 £) | £168 | £443 | £822 |
| Standard error | 16.8 | 44.3 | 82.2 |
| Total Cost PPP (2014/15 £) | | | £216 |

## Amputation

The cost of amputation in the first year of surgery and subsequent years has been reported in a UKPDS costing study(65). The costs were extracted and inflated to 2014/15 prices. The cost of amputation in the first year was £12,254 (standard error £3130) and in subsequent years was £3,403 (standard error £732).

## Blindness

The cost of blindness in the first year of surgery and subsequent years has been reported In a UKPDS costing study (65). The costs were extracted and inflated to 2014/15 prices. The cost of blindness in the first year was £2,067 (standard error £940) and in subsequent years was £1,260 (standard error £138).

## Cancer

The cost of breast and colorectal cancer is estimated as a one-off fixed cost at diagnosis in the model. This simplifying assumption means that the cost of cancer treatment is independent of survival. We acknowlegde that this assumption will affect the timing of costs because all costs are imposed in the first year and subject to less discounting. However, we anticipate that the impact on overall outcomes will not be substantial. A large proportion of costs are will be incurred in the first year of treatment (surgery, chemotherapy, radiotherapy). Costs in subsequent years will be lower for patients who achieve remission and survival will be short in patients who relapse. Therefore, the costs are likely to be skewed to the early years post diagnosis.

A recent appraisal for cancer screening estimated the overall cost of breast cancer as a weighted average depending on the prognosis at diagnosis to be £10,452 in 2006/7 prices and £13,818 when inflated to 2014/15 prices (66).

The cost of colorectal cancer was taken from a screening appraisal which reported the lifetime costs of colorectal cancer according to the Dukes stage of the tumour (67). The appraisal also reported the proportion of cancers identified at each stage, which allowed us to estimate the weighted average cost of colorectal cancer. Table 39 reports the overall cost of colorectal cancer by stage of disease at diagnosis.

Table 40: Estimated cost of colorectal cancer

| Resource component | Dukes’ Stage A | Dukes’ Stage B | Dukes’ Stage C | Stage D |
| --- | --- | --- | --- | --- |
| Number of patients | 3241.92 | 9,431.04 | 7,662.72 | 8,841.60 |
| Prevalence | 0.111 | 0.323 | 0.263 | 0.303 |
| Mean cost per patient | £7,250.84 | £12,441.41 | £19,076.90 | £11,945.78 |
| Price Inflation | | | | 1.296 |
| Mean cost per patient (2014/15) | £10,091 | £17,315 | £26,550 | £16,626 |
| Standard error (2014/15) | £1,009 | £1,732 | £2,655 | £1,663 |
| Total Cost (2014/15) | | | | £18,729 |

## Osteoarthritis

The annual cost of osteoarthritis were estimated in a report in 2010 (68). In this report the authors estimated the expected cost of osteoarthritis from three previous costing studies. The costs include GP attendance, nurse consultations, replacement surgery, help at home and prescription medications. The estimated annual cost of osteoarthritis was £783 in 2008. In the study 93% of the costs were attributable to direct medical costs and 7% to social care. Therefore, cost of direct medical care in 2014/15 prices at £896.

## Depression

Depression is modelled as a chronically recurrent disorder, with patients experiencing further depressive episodes after remission. In the model it is assumed that patients continue to incur costs of depression following an initial diagnosis. These costs reflect ongoing resource use to deal with relapse and prevention of relapse.

A recent trial to prevent secondary depressive episodes collected comprehensive cost data from a sample of individuals with depression (69). The resource uses identified in the control arm were extracted to estimate the costs of depression. The costs from this data (inflated to 2014/15 prices) were not implemented directly into the SPHR diabetes prevention model as this would have over-estimated the number of GP visits. The model already accounts for GP attendance due to depression. Therefore, a revised estimate of the cost of depression, excluding GP consultation was estimated using updated unit costs. The resource use estimates and revised unit cost estimates used to generate a cost of depression excluding GP utilisation are reported in Table 40.

Table 41: Depression utilisation of services and total estimated cost

|  | Assumption for costs | Unit cost | Source | Inflation | Annual utilisation | Source | Cost per year |
| --- | --- | --- | --- | --- | --- | --- | --- |
| Practice nurse at surgery | GP nurse face to face assume 10 mins | £8.83 | (70) | 1.0206 | 1.52 | (69) | £13.70 |
| Practice nurse at home visit | GP nurse face to face assume 30 mins | £26.50 | (70) | 1.0206 | 0.02 | (69) | £0.54 |
| Practice nurse telephone | GP nurse face to face assume 10 mins | £8.83 | (70) | 1.0206 | 0.11 | (69) | £0.99 |
| Health visitor | Health visitor per hour visit 30 mins | £35.50 | (70) | 1.0206 | 0.05 | (69) | £1.94 |
| District nurse | Community nurse 30 mins | £24.50 | (70) | 1.0206 | 0.01 | (69) | £0.38 |
| Other nurse | GP nurse face to face assume 10 mins | £8.83 | (70) | 1.0206 | 0.13 | (69) | £1.17 |
| HCA phlebotomist | Clinical support worker 10 mins | £4.17 | (70) | 1.0206 | 0.31 | (69) | £1.05 |
| Other primary care | Advanced nurse with qualifications | £25.00 | (70) | 1.0206 | 0.19 | (69) | £4.85 |
| Out of hours | Inflated of trial costs | £25.39 | (69) | 1.2045 | 0.23 | (69) | £6.18 |
| NHS direct | Inflated of trial costs | £23.90 | (69) | 1.2045 | 0.09 | (69) | £2.28 |
| Walk-in centre | Inflated of trial costs | £36.70 | (69) | 1.2045 | 0.21 | (69) | £8.15 |
| Prescribed medications | Inflated of trial costs | £9.09 | (69) | 1.2045 | 7.74 | (69) | £74.42 |
| Secondary care | Emergency Medicine, Any Investigation | £109.00 | (71) | 1 | 0.26 | (69) | £21.06 |
|  | | | | | | | £136.71 |

## Dementia

### Cost of Diagnosis

A one off cost of diagnosis is incurred in the first year of the disease to account for the costs associated with assessing and diagnosing patients. The most recent cost study of Dementia for the UK estimated the cost of Dementia diagnosis at £650 in 2012/13 prices (42) inflated to £687.82.

### Ongoing healthcare costs

The direct health care costs of dementia to the NHS were estimated in an Alzheimers UK report in 2014. The costs were estimated from a modelling study based on PSSRU aggregeate long term care model and PSSRU dementia care model. The report describes costs of care for patients with dementia in 2013 £. Full details of the costing model are reported elsewhere. Table 41 reports the costs of dementia for individuals in community or residential care according to MMSE cognitive score. In the model it is assumed that healthcare costs are met entirely by the NHS. These costs are applied in the model to patients with a dementia diagnosis on an annual basis.

Table 42: Average annual direct healthcare dementia costs

|  | Healthcare costs | | Proportion of patients residential care | Total cost | Total Costs 2014/15 £ |
| --- | --- | --- | --- | --- | --- |
|  | Community | Residential |  |  |  |
| Mild (MMSE 21-26) | 2,751 | 4,504 | 10.4% | 2932 | 3103 |
| Moderate (MMSE 10-20) | 2,695 | 9,438 | 76.2% | 7837 | 8293 |
| Severe (MMSE 0-9) | 11,258 | 8,689 | 76.2% | 9300 | 9841 |

# Social Care costs

In this analysis the social care costs refer to the public and private costs incurred with social care as a consequence of a diagnosis with either stroke or dementia. Social care costs associated with the other health outcomes of the model are not included in this estimate. This is likely to under-estimate the overall cost of social care in the population. However, reliable social care costs for other conditions are very hard to obtain because they are less commonly incurred in the prevalent patient population and more likely to be attributed to other factors or ageing more generally.

**Osteoarthritis**

The annual cost of osteoarthritis were estimated in a report in 2010 (68). The estimated annual cost of osteoarthritis was £783 in 2008. In the study 93% of the costs were attributable to direct medical costs and 7% to social care. Therefore, cost of social care costs in 2014/15 prices at £65.

## Stroke

The community costs in the first year following stroke were estimated from the South London Stroke Register (72). The average number of days at day centres, nursing homes, residential home, sheltered accommodation and were used to estimate the social care costs.

|  | Mean number of days | Source | Unit cost per day | Source | Total cost |
| --- | --- | --- | --- | --- | --- |
| Day Centre | 3.9 | (72) | £59 | (47) | 254.38 |
| Nursing Home | 16.9 | (72) | £75 | (47) | 1,265.09 |
| Residential Home | 8.5 | (72) | £101 | (47) | 857.29 |
| Sheltered Home | 8.1 | (72) | £65 | (47) | 526.50 |
| Total cost | | | | | 2878.97 |

## Dementia

The social care costs of dementia were estimated in an Alzheimers UK report in 2014 (42). The costs were estimated from a modelling study based on PSSRU aggregeate long term care model and PSSRU dementia care model. The report describes costs of care for patients with dementia in 2013 £. Table 41 reports the costs of dementia for individuals in community or residential care according to MMSE cognitive score. We used estimates from the Alzheimers UK report to estimate the pubic and private social care costs of dementia in line with the methods used in this report. We do not include the productivity costs of informal carers or other public costs in the model. These costs are applied in the model to patients with a dementia diagnosis on an annual basis.

Table 43: Average annual dementia costs

|  | Healthcare costs | | Proportion of patients residential care | Total cost | Total Costs 2014/15 £ |
| --- | --- | --- | --- | --- | --- |
|  | Community | Residential |  |  |  |
| Mild (MMSE 21-26) | 3,121 | 24,737 | 10.4% | 5362 | 5674 |
| Moderate (MMSE 10-20) | 7,772 | 25,715 | 76.2% | 21455 | 22703 |
| Severe (MMSE 0-9) | 10,321 | 25,874 | 76.2% | 22176 | 23466 |

# Utilities

## Baseline Utility

Baseline utilities for all individuals in the cohort were extracted from the HSE 2011. The tariffs for the responses to the 3 level EQ-5D were derived from a UK population study (73). Utility was assumed to decline due to ageing independent of health status. In the simulation, utility declines by an absolute decrement of 0.004 per year. This estimate is based on previous HTA modelling in cardiovascular disease (7).

## Utility Decrements

The utility decrements for long term chronic conditions were applied to the age adjusted EQ-5D score. In consultation with stakeholders, we assumed that a diagnosis of diabetes was not associated with a reduction in EQ-5D independent of the utility decrements associated with complications, comorbidities or depression. Cardiovascular disease, renal failure, amputation, foot ulcers, blindness, cancer, osteoarthritis and depression were all assumed to result in utility decrements. The utility decrements are measured as a factor which is applied to the individual’s age adjusted baseline. If individuals have multiple chronic conditions the utility decrements are multiplied together to give the individual’s overall utility decrement from comorbidities and complications, in line with current NICE guidelines for combining comorbidities (74).

Due to the number of health states it was not practical to conduct a systematic review to identify utility decrements for all health states. A pragmatic approach was taken to search for health states within existing health technology assessments for the relevant disease area or by considering studies used in previous economic models for diabetes prevention. Discussions with experts in health economic modeling were also used to identify prominent sources of data for health state utilities.

Two sources of data were identified for diabetes related complications. A recent study from the UKPDS estimated the impact of changes in health states from a longitudinal cohort (75). They estimated the impact of myocardial infarction, ischaemic heart disease, stroke, heart failure, amputation and blindness on quality of life using seven rounds of EQ-5D questionnaires administered between 1997 and 2007. This data was used to estimate the utility decrement for amputation and congestive heart failure. The absolute decrement for amputation was converted into utility decrement factors that could be multiplied by the individuals’ current EQ-5D to estimate the relative effect of the complication. Blindness was included in the statistical model used for this analysis however the UKPDS analysis reported an increase in health state utility following a diagnosis with blindness. Discussions with the authors highlighted that this was due to treatment following formal classification with blindness and it was decided that this increase in health state utility should not be included in the cost-effectiveness model.

Utility decrements for renal failure and foot ulcers were not available from the UKPDS study described above. A study by Coffey et al. (2000) was used to estimate utility decrements for renal failure and foot ulcers (76). In this study, 2,048 subjects with type 1 and type 2 diabetes were recruited from specialty clinics. The Self-Administered Quality of Well Being index (QWB-SA) was used to calculate a health utility score.

A meta-analysis of utility values for diabetes and diabetes related complications estimated utility decrements for amputation, ulcer, end stage renal failure and blindness (77). The study pooled utility measures using different health state valuation measures in a meta-analysis. Pooling health state utility values is problematic because of the fact that different valuation methods and different preference-based measures (PBMs) can generate different values on exactly the same clinical health state (78).There were not sufficient studies in the meta-analysis to adjust for the effects of health state valuation measure on the result. This is a limitation of the analysis and we decided that it was preferable to use estimates from single studies.

Utility decrements for cardiovascular events were taken from an HTA assessing statins to reflect the utility decrements in all patients (7) rather than using the UKPDS, which is only representative of a diabetic population. The study conducted a literature review to identify appropriate utility multipliers for stable angina, unstable angina, myocardial infarction and stoke. We used these estimates in the model and assume that transient ischaemic attack is not associated with a utility decrement in line with this HTA.

We identified a systematic review of breast cancer utility studies following consultation with colleagues with experience in this area. The review highlighted a single burden of illness study with a broad utility decrement for cancer (79), rather than utilities by cancer type or disease status. This study was most compatible with the structure of the cost-effectiveness structure. Within this study 1823 cancer survivors and 5469 age-, sex-, and educational attainment-matched control subjects completed EQ-5D questionnaires to estimate utility with and without cancer.

The utility decrement for osteoarthritis was taken from a Health Technology Assessment that assessed the clinical effectiveness and cost-effectiveness of glucosamine sulphate/hydrochloride and chondroitin sulphate in modifying the progression of osteoarthritis of the knee (80).

A review of cost-effectiveness studies highlights the scarcity of studies of health-related quality of life in depression (81). The utility studies identified in the review described depression states by severity and did not adjust for comorbid conditions. Furthermore, the valuations were variable between studies suggesting poor consistency in the estimations. Therefore, it was difficult to apply these in the model. We decided to use a study which had used the EQ-5D in an RCT, for consistency with our utility measure (82). They report an average post treatment utility of 0.67, from which we estimated the utility decrement compared with the average utility reported in the HSE dataset. The decrement was then converted into a relative utility reduction.

The quality of life impact of dementia is estimated from a study by Jonsson and colleagues (83). These utility values were idenfied and used in the most recent NICE HTA for Alzheimer disease (41). A systematic review of health state utilities for alzheimers disease discusses differences in health related quality of life in different settings (84). It is often assumed that patients in institutional settings will be more disabled and have poorer quality of life. However, the studies that compared utility between settings did not identify a statistically significant difference. Therefore, we only related utility to MMSE.

Table 43 reports the multiplicative utility factors that are used in the model to describe health utility decrements from comorbid complications. The mean absolute decrement estimated in each study is reported alongside the baseline utility for each study. The utility factor was estimated by dividing the implied health utility with the comorbidity by the baseline utility.

**Table 44: Utility decrement factors**

|  | Mean Absolute decrement | St. error absolute decrement | Baseline Utility | Multiplicative Utility Factor | Source |
| --- | --- | --- | --- | --- | --- |
| Foot ulcer | -0.099 | 0.013 | 0.689 | 0.856 | Coffey (76) |
| Amputation | -0.172 | 0.045 | 0.807 | 0.787 | UKPDS (75) |
| Blind |  |  |  | 1.00 | Assumption |
| Renal failure | -0.078 | 0.026 | 0.689 | 0.887 | Coffey (76) |
| Stable Angina |  |  |  | 0.801 | Ward HTA (7) |
| Unstable Angina y1 |  |  |  | 0.770 | Ward HTA (7) |
| Unstable Angina y2 |  |  |  | 0.770 | Ward HTA (7) |
| Myocardial Infarction y1 |  |  |  | 0.760 | Ward HTA (7) |
| Myocardial Infarction y2 |  |  |  | 0.760 | Ward HTA (7) |
| Transient Ischaemic Attack |  |  |  | 1.000 | Ward HTA (7) |
| Stroke y1 |  |  |  | 0.629 | Ward HTA (7) |
| Stroke y2 |  |  |  | 0.629 | Ward HTA (7) |
| Breast Cancer | -0.060 | 0.008 | 0.791 | 0.913 | Yabroff (79) |
| Colorectal Cancer | -0.060 | 0.008 | 0.791 | 0.913 | Yabroff (79) |
| Osteoarthritis | -0.101 | 0.069 | 0.791 |  | Black HTA (80) |
| Depression | -0.116 |  | 0.791 | 0.875 | Benedict (82) |
| Congestive Heart Failure | -0.101 | 0.032 |  | 0.875 | UKPDS (75) |
| MMSE 26-30 |  |  | 0.690 |  | Jonsson (83) |
| MMSE 21-25 | -0.05 |  | 0.690 | 0.93 | Jonsson (83) |
| MMSE 15-20 | -0.19 |  | 0.690 | 0.725 | Jonsson (83) |
| MMSE 10-14 | -0.20 |  | 0.690 | 0.710 | Jonsson (83) |
| MMSE 0-9 | -0.36 |  | 0.690 | 0.478 | Jonsson (83) |
| UKPDS baseline utility 0.807; HSE baseline 0.7905 | | | | | |

# Intervention Effectiveness

## Intervention Effectiveness

The effectiveness data used in the model comes from a PHE evidence review of pragmatic lifestyle interventions for prevention of type 2 diabetes (85). This updates a previous review by Dunkley and others (86). Both reviews incorporate meta-analyses of a wide range of different lifestyle interventions aimed at reducing type-2 diabetes, and report a variety of outcomes including type-2 diabetes incidence rate and weight loss. The PHE evidence review also includes some analysis of differential effectiveness in population subgroups and for different intervention characteristics.

PHE, NHS England and Diabetes UK have specified that they wish the commissioned DPP intervention to fulfil 9-12 NICE guidelines as recommended in PH38 (3). NICE guidelines include using particular strategies that are associated with increased effectiveness, specifying the minimum amount of contact time and follow-up sessions, and delivering the programme through qualified practitioners. Both the PHE evidence review and the Dunkley meta-analysis indicate that interventions have increased effectiveness if they fulfil a greater number of NICE guidelines (85;86). In line with this, the model uses the results from the subgroup analysis of interventions fulfilling 9-12 NICE guidelines as the mean effectiveness (weight loss of 3.24kg – Table 12 in the PHE Evidence Review (85)).

Unlike the Dunkley meta-analysis, the PHE evidence review does not report differences in HbA1c, systolic blood pressure (SBP) or cholesterol for this subgroup of interventions. However, it is clear from the Dunkley analysis that there will be concurrent reductions in these other metabolic factors, and that the effectiveness of the intervention would be underestimated in the model if they were not included. To incorporate these changes, the differences in HbA1c, SBP and cholesterol were extrapolated from the Dunkley analysis to reflect the updated weight loss used from the PHE evidence review. This assumes that relationships between changes in metabolic factors are linear. The intervention effectiveness for each metabolic factor used in the model is reported in Table 40.

Table 45: Mean intervention effectiveness used in the model

|  | Mean values from Dunkley et al supplementary Table 7 (76) | **Used in the DPP analysis**: **Default**  Mean weight loss from Table 12 of PHE evidence review for 9-12 NICE guidelines (75) |
| --- | --- | --- |
| Weight (kg) | -2.12 | -3.24 |
| BMI (kg/m^2^) | -0.96 | -1.47 |
| HbA1c (%) | -0.13 | -0.20 |
| Systolic Blood Pressure (mmHg) | -4.3 | -6.57 |
| Total Cholesterol (mmol/l) | -0.18 | -0.28 |

There is good evidence from the PHE evidence review and other studies that intervention effectiveness is unlikely to be uniform across the population, and in particular varies according to the baseline BMI of individuals, those with higher baseline BMI reporting increased weight loss and diabetes risk reduction than those with lower baseline BMI (85;87;88). A differential intervention effect by baseline BMI was therefore implemented in the model. Again this was taken from the PHE evidence review as shown in Table 41 (85).

Table 46: Weight change results per unit baseline BMI from the PHE Evidence Review (85)

| **Subgroup** | **Weight change** | **Unit** | **Study Median** |
| --- | --- | --- | --- |
| BMI | -0.23 kg (-0.53 to 0.07) | Per unit increase in mean study BMI | 31.5 kg/m^2^ |

Personalised intervention effects for each individual, dependent upon their baseline BMI were calculated using the following equation:

**Personalised Intervention Effect = Mean Intervention Effect**

**+ BMI Effect * (Individual BMI – Median BMI)**

Where: Mean Intervention Effect = -3.24 kg

BMI Effect = -0.23 kg

Individual BMI = the baseline BMI of each individual in the population

Median BMI = 31.5 kg/m^2^ (the median of the mean BMI from each study included in the PHE meta-analysis)

For example, for an individual with baseline BMI of 30, the personalised intervention effect would correspond to a weight loss of 2.895kg (smaller than the mean intervention effect), whereas for an individual with baseline BMI of 35, the personalised intervention effect would correspond to a weight loss of 4.045kg (larger than the mean intervention effect). Note that in individuals with BMI < 17.5, the effect of the intervention would be to actually increase weight. However, there are very few such IGR individuals in the model and an intervention focussing on weight loss may not in any case be the best option for individuals who are already underweight.

From this personalised change in weight due to the intervention, individualised changes in BMI, HbA1c, SBP and cholesterol were derived. Individuals in the intervention arm of the model who take up the intervention were assumed to receive this reduction in their metabolic factors instantaneously at the start of the model.

In practice, some individuals who start the intervention will not complete it. The PHE evidence review contains a mixture of studies that have used either intention to treat or complete case analysis (85). Intention to treat analysis takes non-completion into account, whereas complete case analysis does not. However, it is unclear which studies have been used to derive the estimate of effectiveness for 9-12 NICE guidelines. It is likely therefore that the effectiveness estimate used in the model only partially accounts for non-completion and therefore may be higher than is realistic in practice.

The Whitehall II BMI trajectory model estimates an indirect relationship between BMI change and changes in metabolic risk factors. The changes to HbA1c, systolic blood pressure and cholesterol were adjusted to avoid double counting of the indirect effects through BMI and direct effects of the intervention.

## Intervention Costs

The actual intervention cost of the DPP will be determined through the DPP procurement process in early 2016. As this was still undergoing at the time of this analysis, PHE suggested that the mid average cost from their impact assessment of £270 per participant should be used as the default cost. This incorporates expected retention rates of participants, but does not include any local costs of identifying or referring individuals for intervention.

## Duration of Intervention Effect

There is very little published information about how long the effectiveness of intensive lifestyle interventions is likely to endure in participants before weight is regained. In the model, default intervention effectiveness is assumed to decline linearly from its peak at the start of the model until individuals reach the BMI/SBP/HbA1c/cholesterol level that they would have been without intervention. It has been assumed for the analysis that this process takes five years.

## The CAIDE risk score for dementia

The direct treatment effect on dementia was estimated using the CADIE risk score for dementia. This risk score identified obesity, hypertension, and hypercholestaemia as statistically significant mid-life risk factors for dementia. We have used this risk score to approximate a relative risk reduction for the DPP using the observed changes in BMI, systolic blood pressure and total cholesterol.

The CAIDE risk score describes the impact of several vascular risk factors on the incidence of dementia. The risk score is based on 1409 individuals who were studied in mid-life and re-examined 20 years later for signs of dementia. Age, education, hypertension, obesity, and hypercholesterolemia and physical activity were included in the risk model. The vascular risk model was estimated from a logistic regression.

$$P\left( Dementia \right)= \frac{e^{(\beta_{0}+\beta_{1}+\beta_{2})}}{1+e^{(\beta_{0}+\beta_{1}+{\gamma\beta}_{2})}}$$

Where $\beta_{0}$ describes the intercept of the model, $\beta_{1}$ follow-up time, and $\beta_{2}$the regression coefficients of the risk score. The interventions are expected to modify the risk factors included in the risk score and modify the long-term risk of dementia.

Table 47: Logistic regression coefficients for dementia risk in 20 years

|  |  | $\beta$ Coefficient | Odds Ratio | OR 95% CI |
| --- | --- | --- | --- | --- |
| Age | <47 years | 0 | 1 |  |
|  | 47-53 years | 1.804 | 2.958 | (1.261-6.938) |
|  | >53 years | 1.762 | 5.825 | (2.192-15.476) |
| Education | >=10 years | 0 | 1 |  |
|  | 7-9 years | 0.910 | 2.485 | (0.982-6.291) |
|  | 0-6 years | 1.281 | 3.599 | (1.453-8.913) |
| Sex | Female | 0 | 1 |  |
|  | Male | 0.470 | 1.599 | (0.919-2.784) |
| Systolic Blood Pressure | <=140mm Hg | 0 | 1 |  |
|  | >140mm Hg | 0.791 | 2.206 | (1.169-4.161) |
| BMI | <=30kg/m^2^ | 0 | 1 |  |
|  | >30kg/m^2^ | 0.831 | 2.296 | (1.263-4.161) |
| Total Cholesterol | <=6.5mmol/L | 0 | 1 |  |
|  | >6.5mmol/L | 0.631 | 1.879 | (1.012-3.491) |
| Physical Activity | Active | 0 | 1 |  |
|  | Inactive | 0.527 | 1.693 | (0.953-3.009) |
| Follow-up time |  | 0.093 |  |  |
| Intercept |  | -7.642 |  |  |

### Modifying the CAIDE Risk Score

In order to estimate the impact of an intervention on future risk of Dementia it was necessary to transform the vascular risk factors of the CAIDE score onto a continuous scale. The CAIDE risk model used binary definitions for risk characteristics to estimate a simple risk score. However, it is more likely that risk increases over a continuous scale.

The beta coefficients for systolic blood pressure, body mass index and total cholesterol were transformed onto a continuous scale, using the binary odds ratios and population characteristics from the Health Survey for England 2014. We estimated the number of individuals in each CAIDE category, and the mean risk level for each category from data on adults in the Health Survey for England. Table 5 describes the characteristics of adult participants in the Health Survey for England sub-set by the CAIDE risk definitions.

Table 48: CAIDE risk groups from Health Survey for England

|  |  | Health Survey for England | |
| --- | --- | --- | --- |
|  |  | Number | Mean |
| Systolic Blood Pressure | <=140mm Hg | 887 | 152.50 |
|  | >140mm Hg | 3782 | 120.02 |
| Body Mass Index | <=30kg/m^2^ | 1882 | 34.54 |
|  | >30kg/m^2^ | 5063 | 24.91 |
| Total Cholesterol | <=6.5mmol/L | 445 | 7.16 |
|  | >6.5mmol/L | 3456 | 4.94 |

The hazard ratios for each category of behaviour were weighted by the number of people from the HSE model data falling into that category to generate an “implied hazard ratio” (where the reference point with a hazard ratio of 1 is the HSE model data mean rather than the reference category from the CAIDE model). These implied hazard ratios were then plotted against the mean observed behaviour from the HSE data for each behaviour category.

$$Implied OR=\frac{{OR}_{>30kg/m2}}{\left( \frac{\left( N_{\leq30kg/m2}\times{OR}_{\leq30kg/m2} \right)+\left( N_{>30kg/m2}\times{OR}_{>30kg/m2} \right)}{N} \right)}$$

Where OR is the odds ratio, and N describes the number from the Health Survey for England.

= 2.296 / (((1882 * 2.296) + (5063 * 1.00)) / 6945)

= 1.70

$$Implied OR=\frac{{OR}_{\leq30kg/m2}}{\left( \frac{\left( N_{\leq30kg/m2}\times{OR}_{\leq30kg/m2} \right)+\left( N_{>30kg/m2}\times{OR}_{>30kg/m2} \right)}{N} \right)}$$

Where OR is the odds ratio, and N describes the number from the Health Survey for England.

= 1 / (((1882 * 2.296) + (5063 * 1.00)) / 6945)

= 0.74

The mean BMI in the HSE model data falling in the $\leq30kg/m2$ and >30kg/m2 categories were 24.91 and 34.54, respectively. These were plotted against the implied HRs:


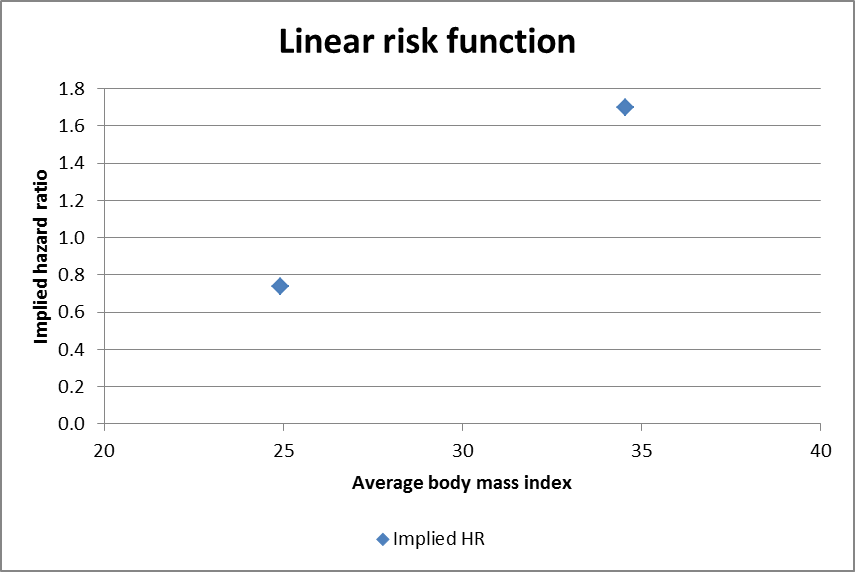


A linear trendline was fitted to these data points and the slope (0.0996) and constant (-1.7413) of this line were estimated using the =LINEST() function in Microsoft Excel 2010 and these values were used to define the continuous dementia risk function for BMI.


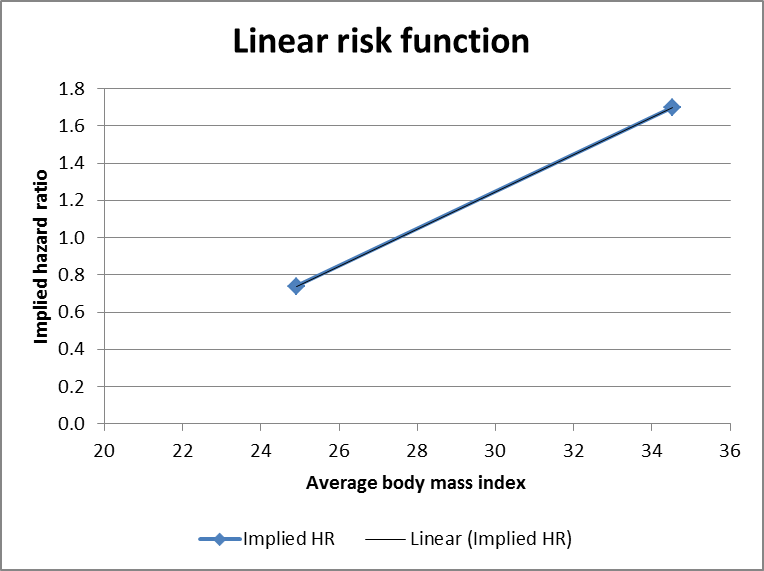


The same approach was taken to estimate continuous risk for systolic blood pressure and Total Cholesterol. Table 46 summarises the estimated odds ratios for unit changes in the three metabolic risk factors.

Table 49: Estimate odds ratios for a unit change in risk factor

|  | Slope ($\beta$) | Odds ratio |
| --- | --- | --- |
| Systolic Blood Pressure | 0.030 | 1.031 |
| Body Mass Index | 0.0996 | 1.105 |
| Total Cholesterol | 0.3604 | 1.434 |

## Estimating the Effect of the Intervention on Dementia

The odds ratio of Dementia with and without the intervention (T) can be estimated from the expected changes ($\Delta$) in systolic blood pressure, BMI and total cholesterol and the slope of the implied log odds ratios $\beta$.

$$OR\left( {Dementia}_{T} \right)=exp(\Delta_{SBP}\beta_{SBP}+\Delta_{BMI}\beta_{BMI}+\Delta_{CHOL}\beta_{CHOL})$$

The expected change in systolic blood pressure, BMI and Total Cholesterol are calculated based on the magnitude and duration of maintenance of benefit. The benefits of interventions are not expected to be permanent. The CAIDE model estimates the risk of dementia in 20 years. The change in risk describes the difference in area under the curve over 20 years between the control and intervention arm. Figure 4 illustrates how the treatment effect on BMI reduces the risk burden with decay over 5 years.

Figure 4: BMI area under the curve for treatment and control describing risk burden


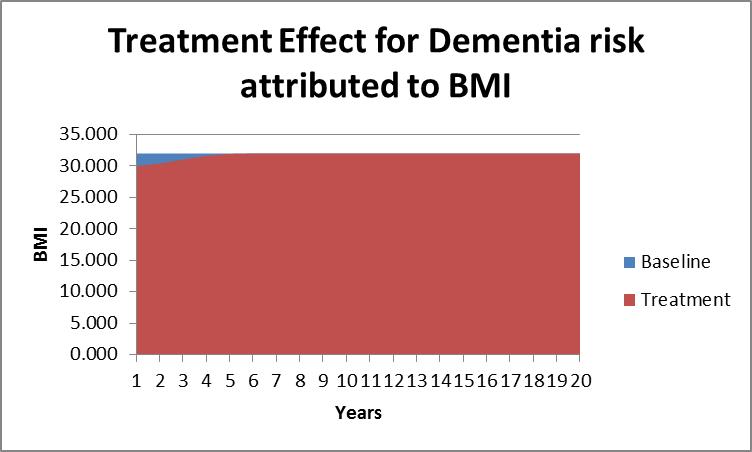


In the model the odds ratio is converted to a risk ratio. If the baseline probability is small the odds ratio approximates the risk ratio, however for some individuals at some timepoints in the model the baseline risk of dementia may be high >10%. Therefore, we convert the treatment Odds Ratio to an individualised risk ratio based on the estimated probability of dementia for the individual at each timepoint.

The risk ratio can be calculated from the odds ratio and baseline probability using the equation below.

$$RR=\frac{OR}{\left( 1+p_{0}+\left( p_{0} \times OR \right) \right)}$$

Where OR is the odds ratio for treatment, and $p_{0}$ is the baseline 20 year probability of dementia from CAIDE.

The 20 year baseline probability of dementia was approximated from the patient characteristics reported from CAIDE. The summary statistics were not perfectly compatible with the variables of the CAIDE risk score (i.e. mean and standard deviation rather than proportion meeting criteria). However, we assumed the data were normally distributed to estimate the proportion of individuals meeting the criteria.

Table 50: Estimated baseline 20 year probability of Dementia in CAIDE

|  | Mean | Standard deviation | Beta coefficient | Proportion |
| --- | --- | --- | --- | --- |
| Age | 50.34 | 5.95 |  |  |
| Age 47-53 |  |  | 1.084 | 0.39 |
| Age >53 |  |  | 1.762 | 0.33 |
| Education | 8.61 | 3.37 |  |  |
| 7-9 years |  |  | 0.910 | 0.23 |
| <7 years |  |  | 1.281 | 0.32 |
| Sex (% male) |  |  | 0.470 | 0.38 |
| SBP | 144.30 | 19.75 |  |  |
| >140mmHg |  |  | 0.791 | 0.59 |
| BMI | 26.56 | 3.71 |  |  |
| >30mg/m2 |  |  | 0.831 | 0.18 |
| Cholesterol | 6.73 | 1.20 |  |  |
| >6.5mmol/L |  |  | 0.631 | 0.60 |
| Physically inactive |  |  | 0.527 | 0.59 |
| Follow-up | 20.9 |  | 0.093 |  |
| Intercept |  |  | -7.642 |  |
| Log Odds |  |  |  | -2.628 |
| 20 year p(dementia) |  |  |  | 0.067 |

The baseline probability of dementia from CAIDE can be used to estimate the relative risk of any proposed dementia reducing intervention using the following formula.

$${RR}_{T}=\frac{{OR}_{T}}{\left( 1+0.067+\left( 0.067 \times{OR}_{T} \right) \right)}$$

The estimated relative risk of dementia for the diabetes prevention programme is specified in Table 48.

Table 51: Estimated relative risk of dementia for the Diabetes Prevention Programme

|  | Mean 1 year treatment effect | Expected 20 year treatment effect | Log Odds Ratio ($\beta$) |
| --- | --- | --- | --- |
| Systolic blood Pressure | -6.57 | -0.8247 | 0.030 |
| Body Mass Index | -1.47 | -0.1845 | 0.0996 |
| Total Cholesterol | -0.28 | -0.0352 | 0.3604 |
| Estimated Odds Ratio | | | 0.9433 |
| Estimated Relative Risk | | | 0.9469 |

The relative risk of dementia was applied to the probability of dementia simulated in the model from the THIN Risk score.

#

# Sensitivity Analysis

One Way Sensitivity Analysis (OWSA) tested the impact groups of pamaters on the overall Net Benefit of the intervention. In this study OWSA tested the key parameters for the new dementia component of the model. OWSA for the other modelling parameters were evaluated in previous modelling studies.

Probabilistic sensitivity analysis (PSA) was enabled in the model to describe the uncertainty in parameter inputs of the model and how this translates into uncertainty in the outcomes of the model. A suitable distribution was selected for each parameter, based upon its mean and standard error. A full list of data inputs into the model and the distribution selected is provided in supplementary file 2. Random sampling simultaneously across all input parameter distributions allowed parameter uncertainty to be quantified. 5000 different random samples of parameter values were selected, and each was applied to a different random cohort of 20,000 individuals. Therefore, the characteristics of patients varied between model runs to ensure that the result was not biased by the baseline characteristics of the individuals. For each PSA sample, the model was run and results compiled. Given the large number of parameters in the model and thus the capacity for error, a thorough process of checking that mean sampling values corresponded to mean parameter values was undertaken to ensure that the results were as accurate as possible.

# Model Validation

The SPHR model has undergone a thorough process of error checking and internal and external validations. Validation of the model to predict metabolic data, diabetes and cardiovascular disease have been reported elsewhere. Here we describe the validation tests used to compared metabolic outcomes with the ELSA data and dementia outcomes with published literature. The validation tests covered four main themes.

1. The evaluate the metabolic trajectory models with inclusion of the ELSA statistical models in individuals aged 61 and over.
2. To evaluate the average rate of decline in MMSE scores.
3. To evaluate the short term incidence of dementia.
4. To evaluate the incidence of long-term outcomes related to older age including coronary heart disease, stroke, dementia and mortality.
5. To evaluate life expectancy following dementia diagnsosis.

Unfortuantely, it was not possible to identify external data sources to validate all four themes. Therefore, summary statistics are presented for 1 and 2, with discussion in relation to some relevant data due to a lack of directly comparable statistics from the published literature. Statistics relating to themes 3 and 4 are directly compared against three published studies summarising data from CFAS and the preDIVA randomised controlled trial (40;89;90).

## Metabolic trajectories

The simulated metabolic trajectories with ELSA statistical models for individuals aged 61 and over are illustrated and compared against the simulated metabolic trajectories with the Whitehall II only for all patients and in 10 year age groups. Figure 4 illustrates the trajectories for all patients. The analysis indicates a shallower trajectory for BMI, HbA1c, Total and HDL cholesterol. The trajectory for systolic blood pressure is mostly unaffected by the inclusion of ELSA data.

Figure 5: Metabolic trajectories for all patients with and without the ELSA statistical models


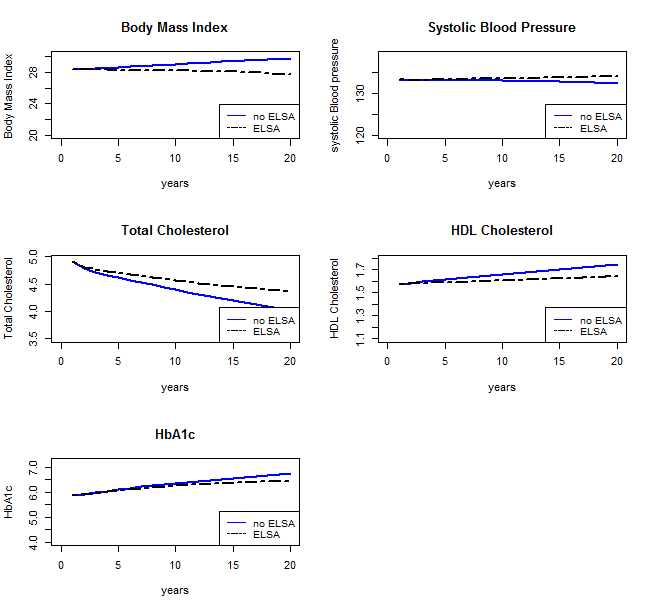


Figure 5 and Figure 6 illustrate the metabolic trajectoriesby 10 year age groups. The trajectories by 10 year age groups follow similar patterns in which the long term trajectories are shallower with the inclusion of the ELSA data. In the 60-70 year old group the rate of decline in BMI is greater with the ELSA statistical models.

Figure 6: Ages 50-60


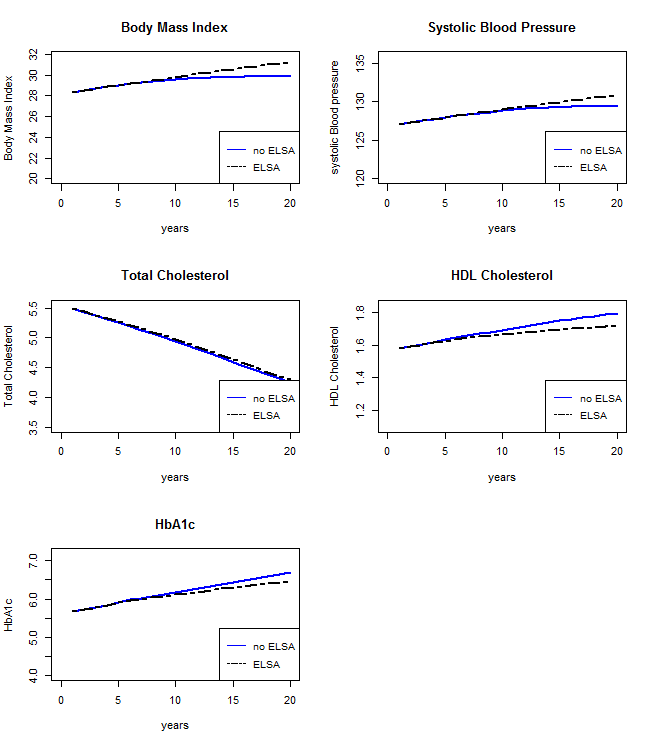


Figure 7: Ages 60-70


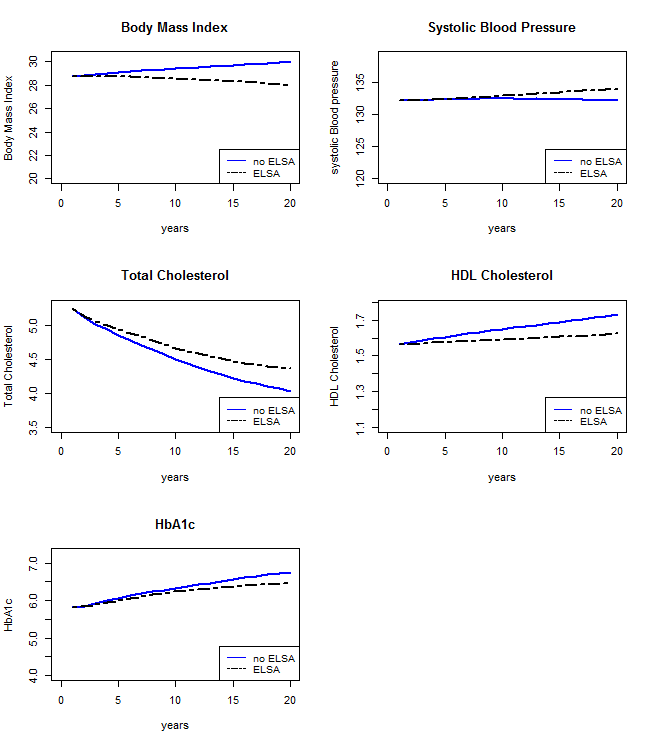


## Short term Dementia Incidence

We compare the modelled dementia incidence with rates reported in the Congitive Function and Ageing Survey (90). The CFAS study reports dementia incidence after 2 years disaggregated by age and gender. Figure 7 and Figure 8 illustrate how the simulation model incidence of dementia compares with the CFAS data. The graphs show that the incidence in men is underestimated at younger ages, but fit relatively well in older age. Whereas the incidence for women fits well at younger age and slightly underestimates in older ages. The model reproduces the substantial increase in risk for women over the age of 80 observed in the data.

Figure 8: Comparison of dementia incidence in men between CFAS and simulation


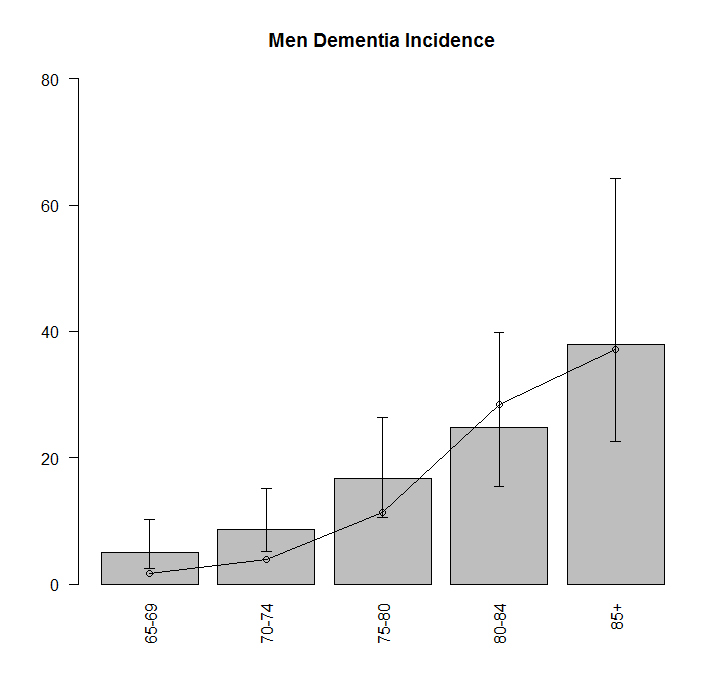


Figure 9: Comparison of dementia incidence in women between CFAS and simulation


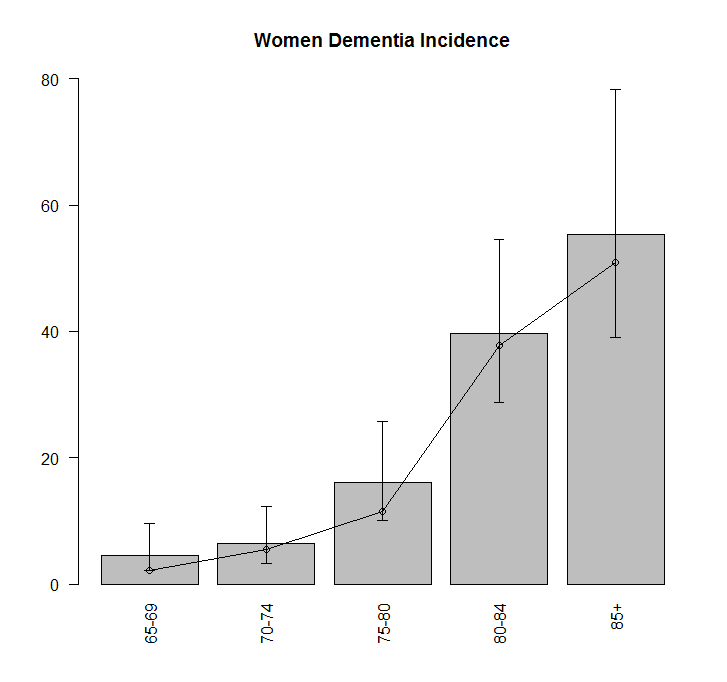


## Long-Term Outcomes

The pre-DIVA trial collected long term data on the incidence of dementia, coronary heart disease, stroke, cardiovascular mortality and all cause mortality (89). The participants were recruited if aged 70-78 and the population included a mean age of 74.5. The participants were followed for 6 years. We simulated a population of 20,000 individuals from the Health Survey for England 2014 aged 70-78 for 6 years to record health outcomes reported in the PreDIVA trial.

|  | Pre-DIVA control arm N (%) | Simulation % |
| --- | --- | --- |
| All cause dementia | 112/1601 (7%) | 5.8% |
| Cardiovascular event | 228/1307 (4%) | 3.3% |
| Myocardial Infarction | 57/1339 (8%) | 7.5% |
| Stroke including TIA | 102/1341 (8%) | 4.7% |
| Cardiovascular death | 60/1425 (4%) | 11.6% |
| All cause mortality | 269/1634 (16%) | 5.8% |

## Life expectancy

Life expectancy for individuals with dementia is estimated from the CFAS cohort to be 4.5 years with a mean age at onset of 84. The simulated life expectancy for individuals following dementia diagnosis in the model was 3.8 years with a mean age at diagnosis of 87. We would expect the age at onset in the CFAS group to be younger than the age of diagnosis in a population diagnosed in usual care, which accouts for the lower life expectancy in the simulation.

## Cost-Effectiveness Outcomes

In order to compare the cost-effectiveness result with previous model specifications we have estimated the incremental outcomes for 20-years with differential discount rates of 6% for costs and 1.5% for QALYs. This enables us to compare the results with the previous cost-effectiveness outcomes generated for the our previous analyses. The results show that the incremental costs and QALYs are slightly higher in the new model specification, with a more up to date baseline population, mortality rate and inclusion of ELSA metabolic trajectories.

Table 52: Comparison of 20-year cost-effectiveness outcomes

|  | **Targeting Strategy (Incremental results vs. Do Nothing)** | |
| --- | --- | --- |
|  | PHE work | DPP no Dementia |
| Net Benefit (£20000 willingness to pay) | £701 | £918 |
| Incremental Health care cost (per person) | -£75 | -22 |
| Incremental Cardiovascular cost (per person) | -£69 | -£83 |
| Incremental QALYs (per person) | 0.0388 | 0.0447 |
| Incremental Life Years (per 1000 people) | 35 | 47 |
| Incremental Diabetes diagnoses (per 1000 people) | -7 | -13 |
| Incremental CVD events (per 1000 people) | -3 | -4 |

# Supplementary Results

## Results by Socioeconomic status

The analysis shows that the grestest incremental QALYs are gained in the third socioeconomic quintile and the most cost savings in the least deprived group. These patterns are not substantially affected by the inclusion of dementia however the cost-savings in the most deprived groups are reduced. Assuming a direct effect of the intervention on dementia incidence increases the incremental QALYs across all socioeconomic quintiles. The inclusion of CAIDE risks has a proportionately smaller impact on incremental costs.

Figure 10: Incremental costs and QALYs by socioeconomic quintile


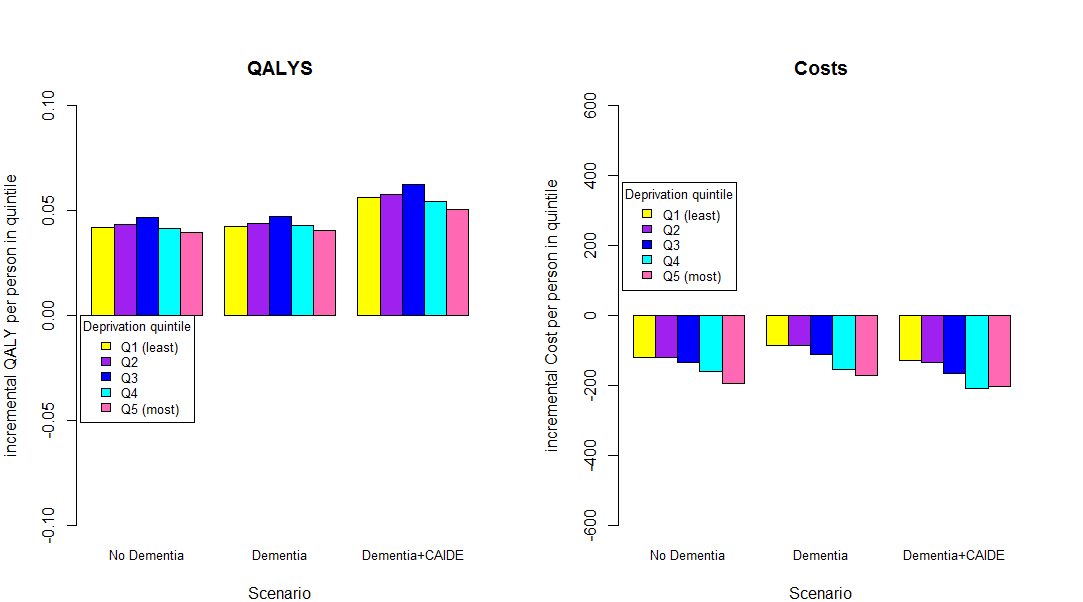


## Sensitivity analyses

The results of the deterministic one-way sensitivity analyses are reported in Table 52.

Table 53: Lifetime Net Benefit for deterministic sensitivity analyses

|  | Dementia | Dementia with CAIDE |
| --- | --- | --- |
| Base Case Analysis | £987 | £1290 |
| Dementia costs low | £987 | £1274 |
| Dementia costs high | £994 | £1321 |
| Dementia utilities low | £984 | £1297 |
| Dementia utilities high | £989 | £1285 |
| Fast MMSE decline | £984 | £1292 |
| Slow MMSE decline | £998 | £1299 |
| Low dementia mortality rate | £983 | £1342 |
| High dementia mortality rate | £998 | £1291 |
| High dementia incidence rate | £956 | £1332 |
| Low dementia incidence rate | £1024 | £1233 |
| Low stroke and diabetes risk association | £976 | £1310 |
| High stroke and diabetes risk association | £995 | £1276 |
| Low MMSE at diagnosis | £981 | £1285 |
| High MMSE at diagnosis | £990 | £1290 |
| CAIDE relative risk low (more effective) | NA | £1404 |
| CAIDE relative risk high (less effective) | NA | £1095 |

# CHEERS Checklist

**CHEERS checklist—Items to include when reporting economic evaluations of health interventions**

| **Section/item** | **Item No** | **Recommendation** | **Reported on page No/ line No** |
| --- | --- | --- | --- |
| **Title and abstract** | | | |
| Title | 1 | Identify the study as an economic evaluation or use more specific terms such as “cost-effectiveness analysis”, and describe the interventions compared. | Page 1 |
| Abstract | 2 | Provide a structured summary of objectives, perspective, setting, methods (including study design and inputs), results (including base case and uncertainty analyses), and conclusions. | Page 2 |
| **Introduction** | | | |
| Background and objectives | 3 | Provide an explicit statement of the broader context for the study. | Page 3 |
|  |  | Present the study question and its relevance for health policy or practice decisions. | Page 4 |
| **Methods** | | | |
| Target population and subgroups | 4 | Describe characteristics of the base case population and subgroups analysed, including why they were chosen. | Page 5  Table 1 |
| Setting and location | 5 | State relevant aspects of the system(s) in which the decision(s) need(s) to be made. | Page 4 |
| Study perspective | 6 | Describe the perspective of the study and relate this to the costs being evaluated. | Page 5  Page 7 |
| Comparators | 7 | Describe the interventions or strategies being compared and state why they were chosen. | Page 6 |
| Time horizon | 8 | State the time horizon(s) over which costs and consequences are being evaluated and say why appropriate. | Page 8 |
| Discount rate | 9 | Report the choice of discount rate(s) used for costs and outcomes and say why appropriate. | Page 8 |
| Choice of health outcomes | 10 | Describe what outcomes were used as the measure(s) of benefit in the evaluation and their relevance for the type of analysis performed. | Page 8 |
| Measurement of effectiveness | 11a | *Single study-based estimates:*Describe fully the design features of the single effectiveness study and why the single study was a sufficient source of clinical effectiveness data. | NA |
|  | 11b | *Synthesis-based estimates*: Describe fully the methods used for identification of included studies and synthesis of clinical effectiveness data. | Page 5 |
| Measurement and valuation of preference based outcomes | 12 | If applicable, describe the population and methods used to elicit preferences for outcomes. | NA |
| Estimating resources and costs | 13a | *Single study-based economic evaluation:*Describe approaches used to estimate resource use associated with the alternative interventions. Describe primary or secondary research methods for valuing each resource item in terms of its unit cost. Describe any adjustments made to approximate to opportunity costs. | NA |
|  | 13b | *Model-based economic evaluation:*Describe approaches and data sources used to estimate resource use associated with model health states. Describe primary or secondary research methods for valuing each resource item in terms of its unit cost. Describe any adjustments made to approximate to opportunity costs. | Page 5  Page 6  Page 7  Table 1 |
| Currency, price date, and conversion | 14 | Report the dates of the estimated resource quantities and unit costs. Describe methods for adjusting estimated unit costs to the year of reported costs if necessary. Describe methods for converting costs into a common currency base and the exchange rate. | Supplementary material Section 7 |
| Choice of model | 15 | Describe and give reasons for the specific type of decision-analytical model used. Providing a figure to show model structure is strongly recommended. | Page 4  Supplementary material: Figure 1 |
| Assumptions | 16 | Describe all structural or other assumptions underpinning the decision-analytical model. | Supplementary material  Page 6  Page 7 |
|  |  |  |  |
| Analytical methods | 17 | Describe all analytical methods supporting the evaluation. This could include methods for dealing with skewed, missing, or censored data; extrapolation methods; methods for pooling data; approaches to validate or make adjustments (such as half cycle corrections) to a model; and methods for handling population heterogeneity and uncertainty. | Page 4  Page 5  Page 6  Page 7  Supplementary Material |
| **Results** | | | |
| Study parameters | 18 | Report the values, ranges, references, and, if used, probability distributions for all parameters. Report reasons or sources for distributions used to represent uncertainty where appropriate. Providing a table to show the input values is strongly recommended. | Table 1  Supplementary Material |
| Incremental costs and outcomes | 19 | For each intervention, report mean values for the main categories of estimated costs and outcomes of interest, as well as mean differences between the comparator groups. If applicable, report incremental cost-effectiveness ratios. | Table 2 |
| Characterising uncertainty | 20a | *Single study-based economic evaluation:*Describe the effects of sampling uncertainty for the estimated incremental cost and incremental effectiveness parameters, together with the impact of methodological assumptions (such as discount rate, study perspective). | NA |
|  | 20b | *Model-based economic evaluation:*Describe the effects on the results of uncertainty for all input parameters, and uncertainty related to the structure of the model and assumptions. | Page 9  Figure 2  Supplementary material |
| Characterising heterogeneity | 21 | If applicable, report differences in costs, outcomes, or cost-effectiveness that can be explained by variations between subgroups of patients with different baseline characteristics or other observed variability in effects that are not reducible by more information. | Table 3 |
| **Discussion** | | | |
| Study findings, limitations, generalisability, and current knowledge | 22 | Summarise key study findings and describe how they support the conclusions reached. Discuss limitations and the generalisability of the findings and how the findings fit with current knowledge. | Page 9  Page 10  Page 11 |
| **Other** | | | |
| Source of funding | 23 | Describe how the study was funded and the role of the funder in the identification, design, conduct, and reporting of the analysis. Describe other non-monetary sources of support. | Page 1 |
| Conflicts of interest | 24 | Describe any potential for conflict of interest of study contributors in accordance with journal policy. In the absence of a journal policy, we recommend authors comply with International Committee of Medical Journal Editors recommendations. | Page 1 |

For consistency, the CHEERS statement checklist format is based on the format of the CONSORT statement checklist

# References

Reference List

(1) Squires H. A methodological framework for developing the structure of Public Health economic models. White Rose ethesis online 2014Available from: URL: <http://etheses.whiterose.ac.uk/5316/>

(2) National Institute for Health and Care Excellence. PH35: Preventing type 2 diabetes: population and community-level interventions. National Institute for Health and Care Excellence 2011NICE public health guidance 35Available from: URL: https://[www.nice.org.uk/guidance/ph35](http://www.nice.org.uk/guidance/ph35)

(3) National Institute for Health and Care Excellence. PH38 Preventing type 2 diabetes - risk identification and interventions for individuals at high risk: guidance. National Institute for Health and Care Excellence 2012NICE public health guidance 38Available from: URL: <http://guidance.nice.org.uk/PH38/Guidance/pdf/English>

(4) NatCen Social Research. Health Survey for England. University College London Department of Epidemiology and Public Health 2014Available from: URL: <http://www.esds.ac.uk/findingData/hseTitles.asp>

(5) Mortality Statistics: Deaths registered in England and Wales (Series DR), 2014. Office of National Statistics 2017Available from: URL: <http://webarchive.nationalarchives.gov.uk/20160105160709/http://www.ons.gov.uk/ons/publications/re-reference-tables.html?edition=tcm%3A77-378961>

(6) Breeze P, Squires H, Chilcott J, Stride C, Diggle PJ, Brunner E, et al. A statistical model to describe longitudinal and correlated metabolic risk factors: the Whitehall II prospective study. J Public Health (Oxf) 2015 Nov 6.

(7) Ward S, Lloyd JM, Pandor A, Holmes M, Ara R, Ryan A, et al. A systematic review and economic evaluation of statins for the prevention of coronary events. Health Technol Assess 2007 Apr;11(14):1-iv.

(8) Green MA, Li J, Relton C, Strong M, Kearns B, Wu M, et al. Cohort Profile: The Yorkshire Health Study. Int J Epidemiol 2014 Jul 9;dyu121.

(9) Rabe-Hesketh S, Skrondal A. Multilevel and Longitudinal Modelling Using Stata. Second edition ed. College Station: StataCorp; 2008.

(10) Arbeev KG, Ukraintseva SV, Akushevich I, Kulminski AM, Arbeeva LS, Akushevich L, et al. Age trajectories of physiological indices in relation to healthy life course. Mech Ageing Dev 2011 Mar;132(3):93-102.

(11) Clarke PM, Gray AM, Briggs A, Farmer AJ, Fenn P, Stevens RJ, et al. A model to estimate the lifetime health outcomes of patients with type 2 diabetes: the United Kingdom Prospective Diabetes Study (UKPDS) Outcomes Model (UKPDS no. 68). Diabetologia 2004 Oct;47(10):1747-59.

(12) Ara R, Pandor A, Stevens J, Rees A, Rafia R. Early high-dose lipid-lowering therapy to avoid cardiac events: a systematic review and economic evaluation. Health Technol Assess 2009 Jul;13(34):1-118.

(13) National Institute for Health and Care Excellence. Hypertension: Clinical management of primary hypertension in adults. 2011. Report No.: CG 127.

(14) Wald DS, Law M, Morris JK, Bestwick JP, Wald NJ. Combination therapy versus monotherapy in reducing blood pressure: meta-analysis on 11,000 participants from 42 trials. Am J Med 2009 Mar;122(3):290-300.

(15) Davies MJ, Heller S, Skinner TC, Campbell MJ, Carey ME, Cradock S, et al. Effectiveness of the diabetes education and self management for ongoing and newly diagnosed (DESMOND) programme for people with newly diagnosed type 2 diabetes: cluster randomised controlled trial. BMJ 2008 Mar 1;336(7642):491-5.

(16) National Institute of Health and Care Excellence. Statins for the prevention of cardiovascular events in patients at increased risk of developing cardiovascular disease or those with established cardiovascular disease. National Institute for Health and Care Excellence; 2006. Report No.: Technology appraisals, TA94.

(17) Watson P, Preston L, Squires H, Chilcott J, Brennan A. Modelling the Economics of Type 2 Diabetes Mellitus Prevention: A Literature Review of Methods. Appl Health Econ Health Policy 2014;12(3):239-53.

(18) Hayes AJ, Leal J, Gray AM, Holman RR, Clarke PM. UKPDS outcomes model 2: a new version of a model to simulate lifetime health outcomes of patients with type 2 diabetes mellitus using data from the 30 year United Kingdom Prospective Diabetes Study: UKPDS 82. Diabetologia 2013 Sep;56(9):1925-33.

(19) D'Agostino RB, Sr., Vasan RS, Pencina MJ, Wolf PA, Cobain M, Massaro JM, et al. General cardiovascular risk profile for use in primary care: the Framingham Heart Study. Circulation 2008 Feb 12;117(6):743-53.

(20) Hippisley-Cox J, Coupland C, Vinogradova Y, Robson J, Minhas R, Sheikh A, et al. Predicting cardiovascular risk in England and Wales: prospective derivation and validation of QRISK2. BMJ 2008 Jun 28;336(7659):1475-82.

(21) McEwan P, Bennett H, Ward T, Bergenheim K. Refitting of the UKPDS 68 risk equations to contemporary routine clinical practice data in the UK. Pharmacoeconomics 2015 Feb;33(2):149-61.

(22) ClinRisk. QResearch 2013Available from: URL: <http://www.qrisk.org/>

(23) Hippisley-Cox J, Coupland C, Robson J, Brindle P. Derivation, validation, and evaluation of a new QRISK model to estimate lifetime risk of cardiovascular disease: cohort study using QResearch database. BMJ 2010 Dec 9;341:c6624. doi: 10.1136/bmj.c6624.:c6624.

(24) Khaw KT, Wareham N, Luben R, Bingham S, Oakes S, Welch A, et al. Glycated haemoglobin, diabetes, and mortality in men in Norfolk cohort of european prospective investigation of cancer and nutrition (EPIC-Norfolk). BMJ 2001 Jan 6;322(7277):15-8.

(25) Kannel WB, D'Agostino RB, Silbershatz H, Belanger AJ, Wilson PW, Levy D. Profile for estimating risk of heart failure. Arch Intern Med 1999 Jun 14;159(11):1197-204.

(26) Kaffashian S, Dugravot A, Brunner EJ, Sabia S, Ankri J, Kivimaki M, et al. Midlife stroke risk and cognitive decline: a 10-year follow-up of the Whitehall II cohort study. Alzheimers Dement 2013 Sep;9(5):572-9.

(27) Johansen NB, Vistisen D, Brunner EJ, Tabak AG, Shipley MJ, Wilkinson IB, et al. Determinants of aortic stiffness: 16-year follow-up of the Whitehall II study. PLoS One 2012;7(5):e37165.

(28) Dadvand P, Rankin J, Shirley MD, Rushton S, Pless-Mulloli T. Descriptive epidemiology of congenital heart disease in Northern England. Paediatr Perinat Epidemiol 2009 Jan;23(1):58-65.

(29) Davies M, Hobbs F, Davis R, Kenkre J, Roalfe AK, Hare R, et al. Prevalence of left-ventricular systolic dysfunction and heart failure in the Echocardiographic Heart of England Screening study: a population based study. Lancet 2001 Aug 11;358(9280):439-44.

(30) Lahmann PH, Hoffmann K, Allen N, van Gils CH, Khaw KT, Tehard B, et al. Body size and breast cancer risk: findings from the European Prospective Investigation into Cancer And Nutrition (EPIC). Int J Cancer 2004 Sep;111(5):762-71.

(31) Renehan AG, Tyson M, Egger M, Heller RF, Zwahlen M. Body-mass index and incidence of cancer: a systematic review and meta-analysis of prospective observational studies. Lancet 2008 Feb 16;371(9612):569-78.

(32) Pischon T, Lahmann PH, Boeing H, Friedenreich C, Norat T, Tjonneland A, et al. Body size and risk of colon and rectal cancer in the European Prospective Investigation Into Cancer and Nutrition (EPIC). J Natl Cancer Inst 2006 Jul 5;98(13):920-31.

(33) Schett G, Kleyer A, Perricone C, Sahinbegovic E, Iagnocco A, Zwerina J, et al. Diabetes is an independent predictor for severe osteoarthritis: results from a longitudinal cohort study. Diabetes Care 2013 Feb;36(2):403-9.

(34) Palmer AJ, Roze S, Valentine WJ, Minshall ME, Foos V, Lurati FM, et al. The CORE Diabetes Model: Projecting long-term clinical outcomes, costs and cost-effectiveness of interventions in diabetes mellitus (types 1 and 2) to support clinical and reimbursement decision-making. Curr Med Res Opin 2004;20(Suppl. 1):S5-S26.

(35) Golden SH, Lazo M, Carnethon M, Bertoni AG, Schreiner PJ, Diez Roux AV, et al. Examining a bidirectional association between depressive symptoms and diabetes. JAMA 2008 Jun 18;299(23):2751-9.

(36) Whyte EM, Mulsant BH, Vanderbilt J, Dodge HH, Ganguli M. Depression after stroke: a prospective epidemiological study. J Am Geriatr Soc 2004 May;52(5):774-8.

(37) Walters K, Hardoon S, Petersen I, Iliffe S, Omar RZ, Nazareth I, et al. Predicting dementia risk in primary care: development and validation of the Dementia Risk Score using routinely collected data. Bmc Medicine 2016;14.

(38) Briggs A, Claxton K, Sculfer M. Decision Modelling for Health Economic Evaluation. 2006.

(39) Wimo A, Religa D, Spangberg K, Edlund AK, Winblad B, Eriksdotter M. Costs of diagnosing dementia: results from SveDem, the Swedish Dementia Registry. Int J Geriatr Psychiatry 2013 Oct;28(10):1039-44.

(40) Xie J, Brayne C, Matthews FE. Survival times in people with dementia: analysis from population based cohort study with 14 year follow-up. BMJ 2008 Feb 2;336(7638):258-62.

(41) Bond M, Rogers G, Peters J, Anderson R, Hoyle M, Miners A, et al. The effectiveness and cost-effectiveness of donepezil, galantamine, rivastigmine and memantine for the treatment of Alzheimer's disease (review of Technology Appraisal No. 111): a systematic review and economic model. Health Technol Assess 2012;16(21):1-470.

(42) Prince M, Knapp M, Guerchet M, McCrone P, Prina P, Comas-Herrera A, et al. Dementia UK Update. Cognitive Function and Ageing Study 2014Available from: URL: <http://www.cfas.ac.uk/files/2015/07/P326_AS_Dementia_Report_WEB2.pdf>

(43) Getsios D, Blume S, Ishak KJ, Maclaine GD. Cost effectiveness of donepezil in the treatment of mild to moderate Alzheimer's disease: a UK evaluation using discrete-event simulation. Pharmacoeconomics 2010;28(5):411-27.

(44) Cancer Survival in England: Patients Diagnosed, 2006–2010 and Followed up to 2011. Office of National Statistics 2012Available from: URL: <http://www.ons.gov.uk/ons/publications/re-reference-tables.html?edition=tcm%3A77-277733>

(45) Seshasai SR, Kaptoge S, Thompson A, Di AE, Gao P, Sarwar N, et al. Diabetes mellitus, fasting glucose, and risk of cause-specific death. N Engl J Med 2011 Mar 3;364(9):829-41.

(46) James BD, Leurgans SE, Hebert LE, Scherr PA, Yaffe K, Bennett DA. Contribution of Alzheimer disease to mortality in the United States. Neurology 2014 Mar 25;82(12):1045-50.

(47) Curtis L. Unit costs of health and social care. 2015.

(48) NHS reference costs 2014-15. Department of Health 2016Available from: URL: https://[www.gov.uk/government/publications/nhs-reference-costs-2014-to-2015](http://www.gov.uk/government/publications/nhs-reference-costs-2014-to-2015)

(49) British National Formulary. <http://www> bnf org/ 2015

(50) CG127 Hypertension: costing template. National Institute for Care and Clinical Excellence 2011Available from: URL: <http://guidance.nice.org.uk/CG127/CostingTemplate/xls/English>

(51) Burr JM, Mowatt G, Hernandez R, Siddiqui MA, Cook J, Lourenco T, et al. The clinical effectiveness and cost-effectiveness of screening for open angle glaucoma: a systematic review and economic evaluation. Health Technol Assess 2007 Oct;11(41):iii-x, 1.

(52) Belsey JD, Pittard JB, Rao S, Urdahl H, Jameson K, Dixon T. Self blood glucose monitoring in type 2 diabetes. A financial impact analysis based on UK primary care. Int J Clin Pract 2009 Mar;63(3):439-48.

(53) Gillett M, Royle P, Snaith A, Scotland G, Poobalan A, Imamura M, et al. Non-pharmacological interventions to reduce the risk of diabetes in people with impaired glucose regulation: a systematic review and economic evaluation. Health Technol Assess 2012 Aug;16(33):1-iv.

(54) Poole C, Tetlow T, McEwan P, Holmes P, Currie C. The prescription cost of managing people with type 1 and type 2 diabetes following initiation of treatment with either insulin glargine or insulin determir in routine general practice in the UK: a retrospective database analysis. Current Medical Research and Opinion 2007;23(1):S41-S48.

(55) Blak BT, Mullins CD, Shaya FT, Simoni-Wastila L, Cooke CE, Weir MR. Prescribing trends and drug budget impact of the ARBs in the UK. Value Health 2009 Mar;12(2):302-8.

(56) Luengo-Fernandez R, Gray AM, Rothwell PM. A population-based study of hospital care costs during 5 years after transient ischemic attack and stroke. Stroke 2012 Dec;43(12):3343-51.

(57) Clarke P, Gray A, Legood R, Briggs A, Holman R. The impact of diabetes-related complications on healthcare costs: results from the United Kingdom Prospective Diabetes Study (UKPDS Study No. 65). Diabet Med 2003 Jun;20(6):442-50.

(58) Palmer S, Sculpher M, Philips Z, Robinsonm M., Ginnelly L, Bakhai A eal. A cost-effectiveness model comparing alternative management strategies for the use of glycoprotein IIb/IIIa antagonists in non-ST-elevation acute coronary syndrome. Report to the National Institute for Clinical Excellence.; 2008.

(59) Youman P, Wilson K, Harraf F, Kalra L. The economic burden of stroke in the United Kingdom. Pharmacoeconomics 2003;21 Suppl 1:43-50.:43-50.

(60) Baboolal K, McEwan P, Sondhi S, Spiewanowski P, Wechowski J, Wilson K. The cost of renal dialysis in a UK setting--a multicentre study. Nephrol Dial Transplant 2008 Jun;23(6):1982-9.

(61) Byrne C, Steenkamp R, Castledine C, Ansell D, Feehally J. UK Renal Registry 12th Annual Report (December 2009): chapter 4: UK ESRD prevalent rates in 2008: national and centre-specific analyses. Nephron Clin Pract 2010;115 Suppl 1:c41-67. doi: 10.1159/000301159. Epub@2010 Mar 31.:c41-c67.

(62) Cost-effectiveness of transplantation. NHS Blood and Transplant . 2013.

Ref Type: Online Source

(63) Gordois A, Scuffham P, Shearer A, Oglesby A, Tobian JA. The health care costs of diabetic peripheral neuropathy in the US. Diabetes Care 2003 Jun;26(6):1790-5.

(64) OECD. Purchasing Power Parities (PPPs) for OECD Countries. <http://stats> oecd org/Index aspx?datasetcode=SNA_TABLE4 2013Available from: URL: <http://www.oecd.org/>

(65) Alva M, Gray A, Mihaylova B, Leal J, Holman R. The impact of diabetes-related complications on healthcare costs: new results from the UKPDS (UKPDS 84). Diabetic Medicine 2014;459-66.

(66) Madan J, Rawdin A, Stevenson M, Tappenden P. A rapid-response economic evaluation of the UK NHS Cancer Reform Strategy breast cancer screening program extension via a plausible bounds approach. Value Health 2010 Mar;13(2):215-21.

(67) Tappenden P, Eggington S, Nixon R, Chilcott J, Sakai H, Karnon J. Colorectal cancer screening options appraisal Report to the English Bowel Cancer Screening Working Group. National Health Service 2004Available from: URL: <http://www.cancerscreening.nhs.uk/bowel/scharr.pdf>

(68) The economic costs of arthritis for the UK economy. Oxford Economics 2014Available from: URL: https://[www.oxfordeconomics.com/publication/open/222531](http://www.oxfordeconomics.com/publication/open/222531)

(69) Chalder M, Wiles NJ, Campbell J, Hollinghurst SP, Searle A, Haase AM, et al. A pragmatic randomised controlled trial to evaluate the cost-effectiveness of a physical activity intervention as a treatment for depression: the treating depression with physical activity (TREAD) trial. Health Technol Assess 2012;16(10):1-iv.

(70) Curtis L. Unit costs of health and social care. 2012.

(71) NHS reference costs 2012-13. Department of Health 2015Available from: URL: https://[www.gov.uk/government/publications/nhs-reference-costs-2012-to-2013](http://www.gov.uk/government/publications/nhs-reference-costs-2012-to-2013)

(72) Grieve R, Porsdal V, Hutton J, Wolfe C. A comparison of the cost-effectiveness of stroke care provided in London and Copenhagen. Int J Technol Assess Health Care 2000;16(2):684-95.

(73) Dolan P, Gudex C, Kind P, Williams A. A social tariff for EuroQoL: Results from a UK general population survey. Discussion Paper No. 138. Centre for Health Economics 1995;University of York(York).

(74) Ara R, Wailoo A. NICE DSU Technical Support Document 12: The use of health state utility values in decision models. 2011.

(75) Alva M, Gray A, Mihaylova B, Clarke P. The Effect of Diabetes Complications on Health-Related Quality of Life: The importance of longitudinal data to address patient heterogeneity. Health Econ 2013 Jul 11;10.

(76) Coffey JT, Brandle M, Zhou H, Marriott D, Burke R, Tabaei BP, et al. Valuing health-related quality of life in diabetes. Diabetes Care 2002 Dec;25(12):2238-43.

(77) Lung TW, Hayes AJ, Hayen A, Farmer A, Clarke PM. A meta-analysis of health state valuations for people with diabetes: explaining the variation across methods and implications for economic evaluation. Qual Life Res 2011 Dec;20(10):1669-78.

(78) Peasgood T, Brazier J. Is Meta-Analysis for Utility Values Appropriate Given the Potential Impact Different Elicitation Methods Have on Values? Pharmacoeconomics 2015 Nov;33(11):1101-5.

(79) Yabroff KR, Lawrence WF, Clauser S, Davis WW, Brown ML. Burden of illness in cancer survivors: findings from a population-based national sample. J Natl Cancer Inst 2004 Sep 1;96(17):1322-30.

(80) Black C, Clar C, Henderson R, MacEachern C, McNamee P, Quayyum Z, et al. The clinical effectiveness of glucosamine and chondroitin supplements in slowing or arresting progression of osteoarthritis of the knee: a systematic review and economic evaluation. Health Technol Assess 2009 Nov;13(52):1-148.

(81) Zimovetz EA, Wolowacz SE, Classi PM, Birt J. Methodologies used in cost-effectiveness models for evaluating treatments in major depressive disorder: a systematic review. Cost Eff Resour Alloc 2012 Feb 1;10(1):1-10.

(82) Benedict A, Arellano J, De CE, Baird J. Economic evaluation of duloxetine versus serotonin selective reuptake inhibitors and venlafaxine XR in treating major depressive disorder in Scotland. J Affect Disord 2010 Jan;120(1-3):94-104.

(83) Jonsson L, Andreasen N, Kilander L, Soininen H, Waldemar G, Nygaard H, et al. Patient- and proxy-reported utility in Alzheimer disease using the EuroQoL. Alzheimer Dis Assoc Disord 2006 Jan;20(1):49-55.

(84) Shearer J, Green C, Ritchie CW, Zajicek JP. Health state values for use in the economic evaluation of treatments for Alzheimer's disease. Drugs Aging 2012 Jan 1;29(1):31-43.

(85) Ashra N, Spong R, Carter P, Davies M, Dunkley A, Gillies Ceal. A systematic review and meta-analysis assessing the effectiveness of pragmatic lifestyle interventions for the prevention of type 2 diabetes mellitus in routine practice. Public Health England; 2015. Report No.: PHE publications gateway number: 2015280.

(86) Dunkley AJ, Bodicoat DH, Greaves CJ, Russell C, Yates T, Davies MJ, et al. Diabetes Prevention in the Real World: Effectiveness of Pragmatic Lifestyle Interventions for the Prevention of Type 2 Diabetes and of the Impact of Adherence to Guideline Recommendations: A Systematic Review and Meta-analysis. Diabetes Care 2014 Apr;37(4):922-33.

(87) Crandall J, Schade D, Ma Y, Fujimoto WY, Barrett-Connor E, Fowler S, et al. The influence of age on the effects of lifestyle modification and metformin in prevention of diabetes. J Gerontol A Biol Sci Med Sci 2006 Oct;61(10):1075-81.

(88) Gillies CL, Abrams KR, Lambert PC, Cooper NJ, Sutton AJ, Hsu RT, et al. Pharmacological and lifestyle interventions to prevent or delay type 2 diabetes in people with impaired glucose tolerance: systematic review and meta-analysis. BMJ 2007;334:229.

(89) Moll van Charante EP, Richard E, Eurelings LS, van Dalen JW, Ligthart SA, van Bussel EF, et al. Effectiveness of a 6-year multidomain vascular care intervention to prevent dementia (preDIVA): a cluster-randomised controlled trial. Lancet 2016 Aug 20;388(10046):797-805.

(90) Matthews FE, Arthur A, Barnes LE, Bond J, Jagger C, Robinson L, et al. A two-decade comparison of prevalence of dementia in individuals aged 65 years and older from three geographical areas of England: results of the Cognitive Function and Ageing Study I and II. Lancet 2013 Oct 26;382(9902):1405-12.

1. The model did not converge when BMI slope was included as a predictor for HDL growth. [↑](#footnote-ref-1)
